# Pan-cancer molecular subtypes of metastasis reveal distinct and evolving transcriptional programs

**DOI:** 10.1016/j.xcrm.2023.100932

**Published:** 2023-02-01

**Authors:** Yiqun Zhang, Fengju Chen, Chad J. Creighton

**Affiliations:** 1 Dan L. Duncan Comprehensive Cancer Center Division of Biostatistics, Baylor College of Medicine, Houston, TX 77030, USA; 2 Human Genome Sequencing Center, Baylor College of Medicine, One Baylor Plaza, MS305, Houston, TX 77030, USA; 3 Department of Medicine, Baylor College of Medicine, Houston, TX 77030, USA

**Keywords:** metastasis, transcriptomics, patient-derived xenografts, PDX, molecular subtypes, pan-cancer, MYC, prostaglandins, EZH2, immune checkpoint

## Abstract

Molecular mechanisms underlying cancer metastasis span diverse tissues of origin. Here, we synthesize and collate the transcriptomes of patient-derived xenografts and patient tumor metastases, and these data collectively represent 38 studies and over 3,000 patients and 4,000 tumors. We identify four expression-based subtypes of metastasis transcending tumor lineage. The first subtype has extensive copy alterations, higher expression of MYC transcriptional targets and DNA repair genes, and bromodomain inhibitor response association. The second subtype has higher expression of genes involving metabolism and prostaglandin synthesis and regulation. The third subtype has evidence of neuronal differentiation, higher expression of DNA and histone methylation genes and EZH2 transcriptional targets, and BCL2 inhibitor response association. The fourth subtype has higher expression of immune checkpoint and Notch pathway genes. The metastasis subtypes reflect expression differences from paired primaries, with subtype switching being common. These subtypes facilitate understanding of the molecular underpinnings of metastases beyond tissue-oriented domains, with therapeutic implications.

## Highlights

• Four cancer cell-intrinsic, pan-cancer molecular subtypes are defined using PDX models
• Subtypes are present in patient metastases, primary tumors, and cancer cell lines
• Subtypes respectively involve MYC, prostaglandins, EZH2, and immune checkpoints
• Subtype switching is common between metastasis and paired primary by patient

## Graphical abstract

**Figure:**
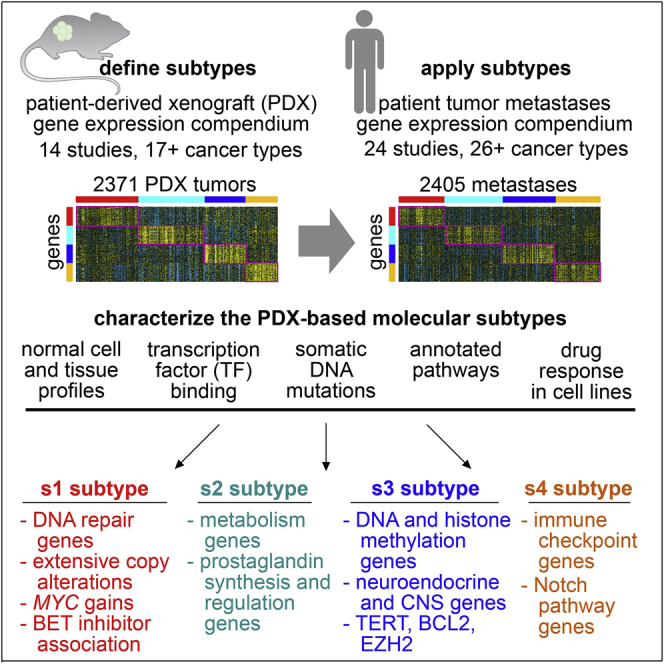

## Teaser

Analyzing gene expression data from patient-derived xenografts and patient metastases, collectively representing 38 studies and more than 3,000 patients and 4,000 tumors, Zhang et al. identify four pan-cancer expression-based subtypes of metastasis transcending tumor lineage. These subtypes have implications for applying existing therapies or developing new therapeutic approaches.

## Introduction

Metastasis is the process by which cancer cells leave the primary tumor site and adapt to a distant tissue microenvironment.^1^ Metastasis causes most cancer deaths.^1^^,^^2^^,^^3^ Invasion and metastasis represent complex processes initiated later in the disease process, with associated molecular mechanisms broadly common across multiple cancer types defined by tissue of origin.^3^^,^^4^ There is a clear need to understand better the processes and pathways underlying metastasis. Global molecular profiling is one approach that can lead to additional insights. For example, transcriptional profiling of metastases and primary tumors taken from the same patient could assess relative changes in gene expression that occur in the metastases. Previous studies have transcriptionally profiled patient tumor metastases, with the associated datasets deposited into the public domain. Most of these studies have focused on a specific cancer type, but other studies involved multiple cancer types and tissues of origin.^5^^,^^6^ There is potential in combining molecular profiling data from individual metastasis studies, with available data to date involving thousands of patients and spanning multiple cancer types.

Cancer is a heterogeneous disease, and molecular subtyping of cancers can help identify pathways and processes underlying specific cancer subsets. With breast cancer being a well-known example, molecular subtypes can point to optimal therapeutic approaches for an individual tumor.^7^ Based on transcriptome data from over 10,000 patient tumors in The Cancer Genome Atlas (TCGA), representing 32 different cancer types, we previously found that these tumors could be grouped into 10 major pan-cancer classes or subtypes.^8^ By virtue of our analytical approaches, these TCGA-based pan-cancer subtypes spanned tissue of origin and tumor histology. The pan-cancer subtypes reflected the results of previous molecular profiling studies of individual cancer types, including subtypes related to cancer cell proliferation, immune cell infiltration, and cancer-associated stroma.^8^ Except for TCGA melanoma cases, all but a small minority of TCGA tumors represent primary tumors and not metastases.^9^ Our pan-cancer molecular subtyping approaches remained to be applied to patient tumor metastases.

Tumors resected from patients represent a mixture of cancer and non-cancer cells, as reflected in their molecular profiles. The tumor microenvironment would include immune cells, fibroblasts, and endothelial cells, all of which may be conscripted by the cancer cells to play a role in tumor biology.^3^ In addition, human metastasis samples would include non-cancer tissues from the biopsy site, representing a major confounder in distinguishing true biology from technical artifact. Effective deconvolution of the contribution of cancer versus normal expression in the tumor expression profile can be challenging.^10^ One approach to address this issue is to profile tumors from patient-derived xenografts (PDXs), whereby a fragment of a patient’s tumor is implanted into a mouse. In PDXs, the stromal components of the original tumor are substituted by their murine counterparts as a result of xenotransplantation.^11^ A gene expression profile of a PDX tumor reflects human gene transcripts from the cancer cells, where the contribution of mouse transcripts from the tumor stroma would be minimal.^11^^,^^12^^,^^13^^,^^14^ PDX models would also represent a type of metastasis, as cancer cells taken from their primary site are made to adapt to a foreign tissue microenvironment.

This study aimed to define pan-cancer molecular-based subtypes of metastasis that would transcend tumor lineage. To this end, we assembled two compendium expression datasets from the public domain, one of PDXs and one of patient tumor metastases, these data collectively representing over 3,000 patients and 38 studies. We removed cancer type- and laboratory-specific differences from each individual published dataset,^8^^,^^15^ allowing for the identification of pan-cancer phenomena that would span data from multiple studies. We followed a previously demonstrated approach,^11^ but applied here to metastasis and greatly expanded to incorporate multiple cancer types and studies. We used the PDX compendium expression dataset to define four expression-based molecular subtypes to minimize the contribution of non-cancer cells. We then applied these molecular subtypes to profiles of patient tumor metastases. We examined the subtype-associated differential expression patterns in the context of metastases versus paired primary differences within the same patient. We could also characterize the metastasis subtypes in terms of associated pathways, copy number alterations, and integration with results of external studies.

## Results

### Compendium expression datasets of PDXs and patient tumor metastases

For our study, we assembled three separate compendium mRNA expression datasets representing metastases, with the data involved being publicly available from 38 individual studies (Tables S1). Our compendium dataset of PDXs represented 2,371 tumors, 973 patients, 14 studies, and over 17 cancer types by tissue of origin (including colorectal, n = 894 tumors; skin, n = 218; sarcoma, n = 214; breast, n = 213; head/neck, n = 165; bladder, n = 150; pancreatic, n = 135; gastric, n = 117; lung, n = 86; kidney, n = 58; uterine, n = 29; medulloblastoma, n = 20; prostate, n = 17; glioblastoma, n = 12; cervical, n = 10; ovarian, n = 9; other, n = 24). Our compendium dataset of patient tumor metastases resected from patients represented 2,405 tumors, 2,158 patients, 24 studies, and over 26 cancer types (including colorectal, n = 695 tumors; breast, n = 413; prostate, n = 349; skin, n = 146; sarcoma, n = 99; ovarian, n = 90; pancreatic, n = 79; lung, n = 75; kidney, n = 65; liver-biliary, n = 52; head/neck, n = 51; thyroid, n = 48; bladder, n = 28; secretory, n = 27; lymphoma, n = 25; esophagus, n = 24; CNS, n = 23; gastric, n = 33; other, n = 83). Of the 2,405 tumors in the patient tumor metastasis compendium dataset, 307 patient tumor metastases (representing 291 patients and 8 cancer types) had a corresponding primary tumor pair from the same patient also profiled, allowing for paired analyses for differences in metastases versus primaries. To the individual datasets involved in the compendiums, we applied previously demonstrated analytical approaches (see STAR Methods)^8^^,^^15^^,^^16^^,^^17^ to effectively erase expression differences according to laboratory, analytical platform, or cancer type. These approaches allowed us to identify global patterns that would cut across multiple datasets and cancer types.

Our study approach was first to define expression subtypes and associated differential genes using our PDX compendium dataset and then to classify each patient tumor metastasis expression profile according to these PDX-based subtypes. We characterized the salient features of each subtype, as described below. For our study, PDX tumors would represent metastases, with cancer cells taken from their original site and made to grow at a different site. The advantage of defining molecular subtypes using PDX models is that the contribution of non-cancer cells to the PDX tumor profile is minimized. RNA from mouse cells either hybridized to a human expression array chip or sequenced and aligned to the human genome yields a much lower signal than RNA from the human cancer cells.^11^^,^^12^^,^^13^^,^^14^ In contrast, the profiles in our patient tumor metastasis compendium dataset would represent mixtures of cancer and stroma cells.^10^ For each molecular subtype, we could determine which of the associated subtype-specific genes, based on analysis of the PDX dataset, were also differentially expressed in metastasis versus primaries by paired analysis, using our compendium of 307 metastases with primary pairs. Within most tissue-based cancer types represented in the compendium dataset, widespread differences between metastasis and primary by paired analysis were identifiable (Figures S1A and S1B and Table S2). However, a likely confounder here would involve differences in non-cancer cells between the primary site and the metastasis biopsy site (Figure S1C).

### Expression-based subtypes of tumor metastases

We set out to identify molecular subtypes in our patient tumor metastasis compendium dataset. As a starting point, we classified PDX and patient tumor metastases expression profiles according to a set of pan-cancer subtypes—labeled c1 through c10—previously defined using TCGA datasets of predominantly primary tumors^8^ (Figure S2A). The TCGA-based subtypes were represented in both PDX and patient tumor metastases, but notably with relatively fewer PDX tumors and weaker patterns for the TCGA “c3” and “c7” subtypes involving immune cells and tumor stroma, respectively. We then used the PDX compendium expression dataset to define molecular subtypes, to minimize the contribution of non-cancer cells. In contrast, subtypes defined using the patient tumor metastasis compendium could be confounded, for example, by the tissue biopsy site, which usually differs from the primary site (Figures S2B and S2C). Using a randomly selected set of 2,000 genes, the 2,371 PDX tumors in our compendium dataset separated into four distinct expression-based pan-cancer subtypes based on unsupervised clustering, labeled s1 through s4 (Figures S2D–S2F). In PDX tumors, the differential expression patterns associated with the subtypes were associated with human cancer cells over mouse stroma cells, as evidenced, for example, by analysis of PDX RNA-sequencing (RNA-seq) data aligned to both human and mouse genomes (Figure S3).

**Figure 1:**
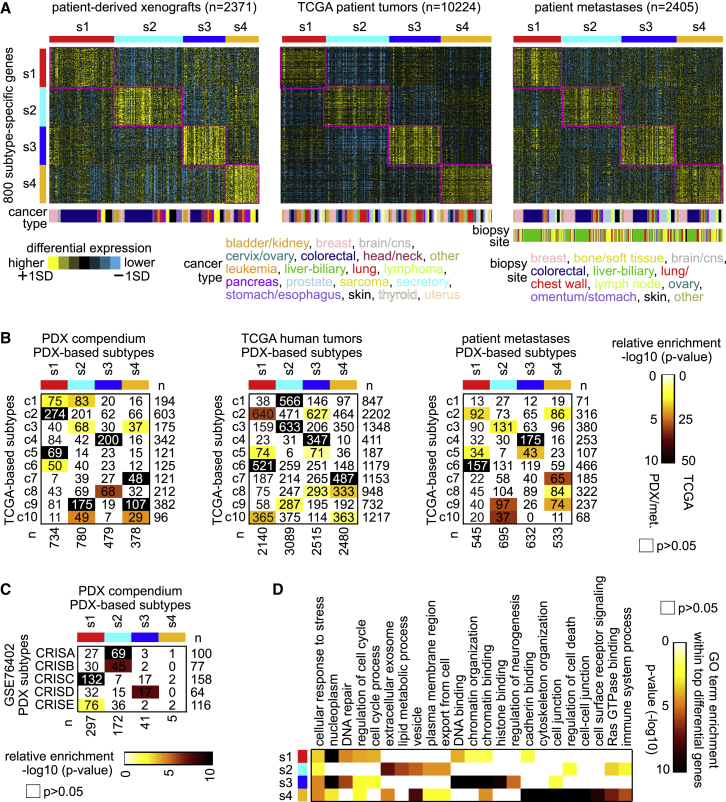
Pan-cancer molecular subtyping of tumor metastases (A) The PDX compendium expression dataset defined four pan-cancer subtypes, s1 through s4 (see STAR Methods and Figure S2). Transcriptomic patterns for the top set of 800 mRNAs distinguishing between the four PDX-based subtypes are shown for PDX, TCGA, and patient tumor metastases datasets, with subtype-specific expression patterns highlighted. SD, standard deviation from the median within a given dataset and within cancer type. (B) For PDX compendium, TCGA, and patient tumor metastases compendium datasets, the significance of overlap between the PDX-based subtype assignments and TCGA-based subtype assignments is indicated. (C) For GSE76402 colorectal (CRC) PDXs in the PDX compendium 11, significance of overlap between our PDX-based subtype assignments and the CRC intrinsic subtypes (CRISs) based on the GSE76402 study. (D) For the top overexpressed genes associated with each PDX-based subtype (from A), represented GO categories were assessed, with selected enriched categories represented here. For (B), (C), and (D), p values are by one-sided Fisher’s exact test.

We classified profiles in the patient tumor metastasis compendium and TCGA datasets by PDX-based molecular subtype, using a gene classifier consisting of the top set of 800 differential mRNAs (Figure 1A, genes defined by comparing each subtype to the rest of the tumors). The PDX-based subtypes were well represented in both these datasets at similar proportions. For TCGA tumors, of which a substantial number have proteomic data by mass spectrometry or by reverse-phase protein array, the PDX-based subtypes, as defined above at the mRNA level, were also reflected at the protein level (Figures S4A–S4D). Among the three datasets (PDX, patient metastasis, TCGA), there were significant patterns of overlap between the s1–s4 PDX-based subtype assignments and the previous TCGA c1–c10 subtype assignments (Figure 1B). Specifically, the current and previous subtyping correspondence included s1 to c2/c5/c6, s2 to c3/c9, s3 to c4, and s4 to c7. The c4 subtype was previously associated with neuroendocrine-like tumors. In addition, GSE76402 colorectal (CRC) PDX sample profiles in our compendium showed significant overlaps between our PDX-based subtype assignments and previously identified CRC intrinsic subtypes (CRISs),^11^ with three of our four subtypes overlapping with four of the five CRIS subtypes (Figure 1C). Within the top differentially expressed genes underscoring each PDX-based subtype, specific gene categories (by Gene Ontology [GO] annotation) were overrepresented (Figure 1D and Table S3). Subtype s1 involved “DNA repair” and “cell-cycle process” genes; subtype s2 involved “extracellular exosome” genes; subtype s3 involved “DNA repair,” “chromatin organization,” and “histone binding” genes; and subtype s4 involved “cell junction,” “Ras GTPase binding,” and “immune system process” genes. Overall, the PDX-based subtypes did not strongly associate with patient metastasis biopsy site or tissue of origin. However, the s1 and s4 subtypes were enriched and antienriched, respectively, for CRC cases (Figures S2E and S2F).

### Metastasis subtypes reflect expression differences from paired primaries

**Figure 2:**
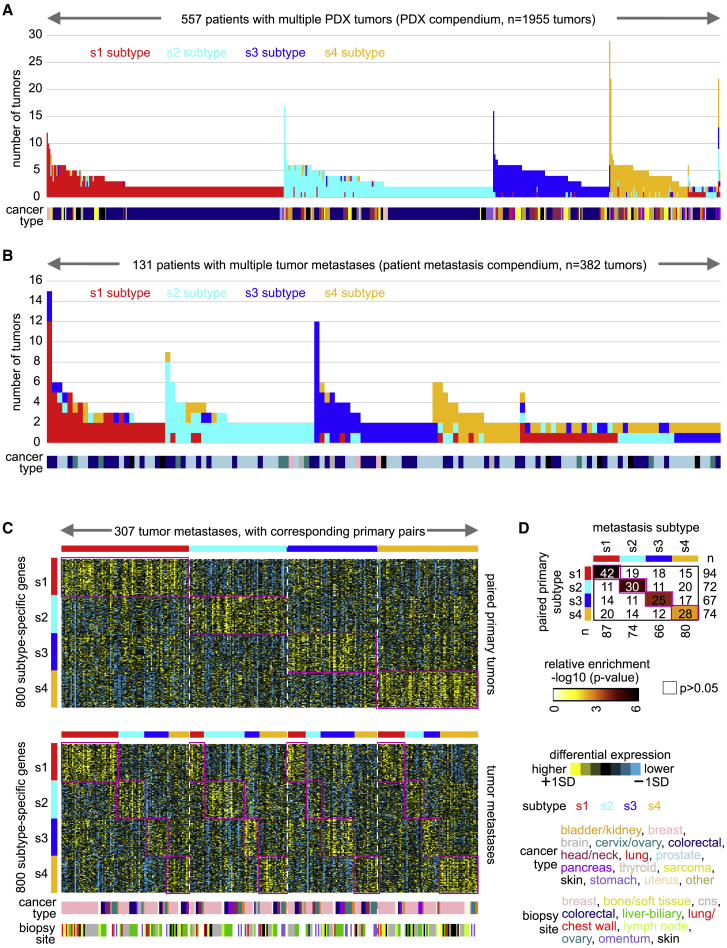
Molecular subtype assignments involving multiple tumors from the same patient (A) By patient, molecular subtype assignments for 1,955 PDX tumors involving 557 patients with multiple PDX tumors represented in the PDX compendium expression dataset. Patients are sorted according to those for whom a plurality of tumors were of the same subtype (left) and those for whom no single subtype was represented in a plurality of tumors (right). (B) By patient, molecular subtype assignments for 382 patient tumor metastases involving 131 patients with multiple tumor metastases represented in the patient metastasis compendium dataset. Patients are sorted according to those for whom a plurality of tumors were of the same subtype (left) and those for whom no single subtype was represented in a plurality of tumors (right). (C) For the 307 patient tumor metastasis expression profiles for which expression profiles for the paired primary were available, both the primary and the metastasis were classified for the PDX-based subtypes. The expression heatmap represents the subtype-associated expression patterns of the tumor metastases in relation to the patterns for the corresponding paired primary tumors. (D) For the 307 tumor metastases represented in (C), the overlaps between the metastasis-based and the paired primary-based subtype assignments are shown. The p values were found by one-sided Fisher’s exact test. For 125 of the 307 tumor metastases (41%), the metastasis-based and primary-based subtype assignments were the same.

Multiple tumors from the same patient tended to share the same molecular subtype assignments. However, the subtype assignments would differ in a substantial fraction of cases, representing molecular subtype switching.^18^ Of the 2,371 tumors in our PDX compendium expression dataset, 1,955—representing 557 patients—involved multiple tumors (two or more) originating from the same patient. Of the 557 patients, 530 (95%) had a plurality of tumors with the same subtype (Figure 2A). In our patient metastasis compendium expression dataset, 381 tumors—representing 131 patients—involved multiple tumors from the same patient, with 92 patients (70%) having a plurality of tumors with the same subtype (Figure 2B). For the 307 patient tumor metastases in our compendium for which paired primary data were available, we classified both primary and metastasis by PDX-based subtype. In most cases, the assigned subtype and associated expression patterns differed between the metastasis and the paired primary (Figure 2C). However, for 125 of the 307 tumor metastases (41%), the metastasis-based and primary-based subtype assignments were the same, these overlapping assignments being statistically significant (Figure 2D). In many instances, subtype switching events in patient metastases could be associated with expression changes in immune cell markers involving the s4 subtype (Figures S5A and S5B).

**Figure 3:**
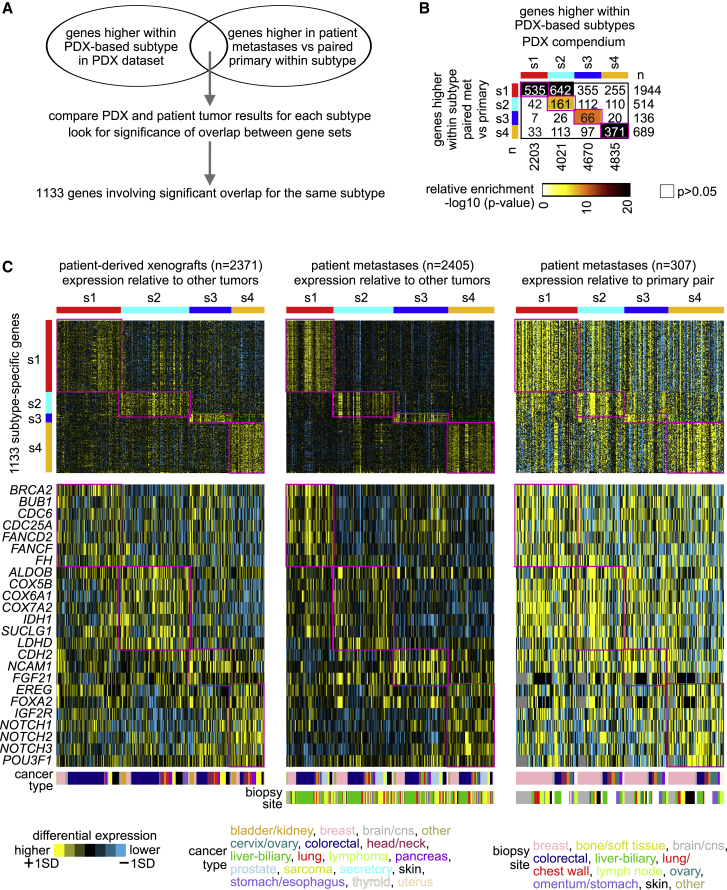
Subtype-specific gene expression differences overlap highly with patient metastasis versus paired primary differences (A) Schematic of gene set comparisons. For each PDX-based subtype, the set of genes high within that subtype versus the rest of the PDX tumors was overlapped with the set of genes high in patient metastases of the same PDX-based subtype versus the corresponding paired primaries. A top set of 1,133 genes involves significant gene set overlaps between PDX comparisons and paired patient metastasis comparisons for the same subtype, involving each of the four subtypes. (B) Significance of overlap between the genes high within each of the PDX-based subtypes (using t test, p < 0.01, based on analysis of PDX compendium) and the genes high within paired patient metastasis versus primary within each subtype (p < 0.01, paired t test, based on analysis of the patient tumor metastasis compendium). Overlap p values were found by one-sided Fisher’s exact test or chi-square test. From these results, a set of 1,133 genes involves significant gene set overlap (p < 1E−6) for the same subtypes (e.g., 535 overlapping s1–s1 genes, 161 s2–s2 genes, etc.). (C) Differential expression patterns for the top set of 1,133 genes involving significant gene set overlaps for any of the four PDX-based subtypes are shown across the PDX compendium dataset (differential expression relative to other tumors), patient tumor metastases compendium dataset (relative to other tumor metastases), and patient tumor metastasis versus paired primary compendium dataset (relative to primary pair). Subtype-specific expression patterns are highlighted. Selected genes of interest from the 1,133 genes are also represented individually by differential patterns.

We wanted to explore expression differences between our molecular subtypes of metastasis in the context of metastasis versus paired primary comparisons involving metastasis-related changes within the patient. Our PDX compendium dataset defined differential expression patterns among the subtypes, while our paired metastasis and primary dataset could evaluate expression changes in metastasis using the primary as a baseline. Different gene sets from the respective datasets would represent orthogonal results, with significant correspondence between gene sets for the same molecular subtype of particular interest (Figure 3A). We identified highly significant gene set overlaps between PDX and paired patient metastasis comparisons for the same subtype, involving all four subtypes and 1,133 genes (Figure 3B and Tables S2 and S3). Differential subtype-specific expression patterns for these 1,133 genes appeared consistent across our PDX compendium dataset and patient tumor metastases compendium dataset, where we assessed differential expression for the latter relative to other metastases and relative to available primary pairs (Figure 3C). We also observed similar correspondence patterns between PDX and paired metastasis comparisons for genes with lower expression by subtype, involving 1,459 genes (Figures S5C–S5E). Subtype-specific genes involving the above included DNA repair-related genes for s1 subtype (*BRCA2*, *FANCD2*, *FANCF*), metabolism-related genes for s2 subtype (*ALDOB*, *COX5B*, *COX6A1*, *COX7A2*, *IDH1*, *SUCLG1*, *LDHD*), neuroendocrine marker genes for s2 subtype (*CDH2*, *NCAM1*), and Notch pathway genes for s4 subtype (*NOTCH1*, *NOTCH2*, *NOTCH3*; Figure 3C). Given that some 60% of metastases may have a different subtype from their corresponding primary tumor (Figure 3D), many observed differences between paired metastases and primaries may also involve subtype switching.

### Central nervous system (CNS) and transcription factor (TF) associations by subtype

**Figure 4:**
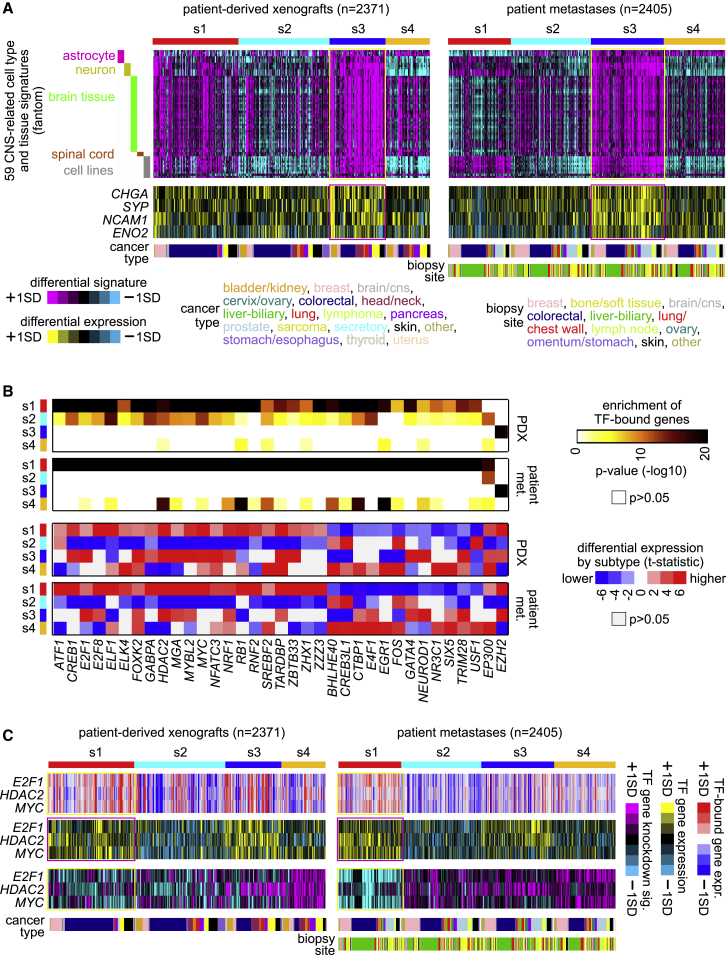
Central nervous system (CNS) and transcription factor (TF) associations by subtype (A) CNS associations by subtype. Heatmaps showing intersample correlations (purple, positive; cyan, negative) between mRNA profiles of tumors in PDX and patient metastasis compendium datasets (columns) and mRNA profiles of Fantom^20^ cell types or tissues related to the CNS (rows). (B) Top TF associations by subtype. Encode^21^ data on TF binding was extracted for 158 TFs, with gene associations defined by binding within 2 kb upstream of the gene start. For the set of 35 TFs represented, there was both significant overlap (p < 1E−6, one-sided Fisher’s exact test or chi-square test) between the TF-bound genes and the genes overexpressed in the expression subtype and significantly higher or lower levels of the TF gene in that same subtype (p < 0.05 by t test), for both PDX and patient metastasis compendium datasets. (C) Of the 35 TFs represented in (B), 10 were represented in an expression profiling dataset of siRNA knockdown of specific genes.^22^ For three TFs—E2F1, HDAC2, and MYC—the genes were highly expressed in the associated s1 subtype, with the corresponding siRNA knockdown signature scoring significantly negative (p < 1E−6, t test) for both PDX and patient tumor metastases datasets. For these three TFs, the associated patterns are represented across PDX and patient tumor metastasis compendium datasets: average differential expression of the TF-bound genes (red-blue heat maps), differential expression of the TF genes (yellow-blue heat maps), and differential levels of the TF gene siRNA signature (purple-cyan heat maps). A negative siRNA signature association indicates that knocking down the TF gene would result in a global pattern opposite TF gene overexpression.

Our s3 metastasis subtype was strongly associated with the previously identified “c4” pan-cancer molecular subtype, which expressed markers of neuroendocrine tumors.^8^ Neuronal differentiation occurring in epithelial cancer cells has been observed elsewhere.^19^ To explore this association further, we examined the expression dataset from the Fantom consortium of 889 profiles representing various normal human cell and tissue specimens,^20^ including 59 CNS-related cell types and tissues. Gene signatures of several CNS profiles—including those from neurons, astrocytes, whole brain, and spinal cord—were strongly manifested in s3, relative to the other subtypes, across PDX and patient tumor metastasis compendium datasets (Figure 4A). Furthermore, genes encoding canonical markers of neuroendocrine tumors—including *CHGA* (chromogranin A), *SYP* (synaptophysin), *NCAM1* (CD56), and *ENO2* (neuron-specific enolase)—were all differentially higher in s3 tumors (Figures 4A and S6 and Tables S3 and S4).

We surveyed TF binding upstream of each gene for the genes associated with higher subtype-specific expression (Table S5). Of 158 TFs with available data,^21^ 87 were significantly enriched (p < 1E−6, one-sided Fisher’s exact or chi-square test) with one or more of the subtype-specific gene sets, for both PDX and patient tumor metastases compendium datasets. Of the 87 TFs, 35 involved differential expression of the TF gene in the same subtype in both compendium datasets (Figure 4B). Most of these significant TF associations involved the s1 subtype, although notably, the s3 subtype showed both higher expression of the EZH2 gene and a strong enrichment for EZH2 transcriptional targets within genes higher in s3 versus other tumors. When integrating the above results with a limited set of gene expression signatures of TF knockdown by siRNA,^22^ three TFs—E2F1, HDAC2, and MYC— had siRNA knockdown signature scoring significantly negative for the s1 subtype, along with the corresponding higher TF gene expression and TF gene target enrichment patterns (Figure 4C). The above MYC association is consistent with previous observations in primary tumors for the s1 subtype analogs.^8^^,^^16^^,^^17^ As the compendium expression datasets involved our first removing cancer type-specific differences to arrive at our pan-cancer subtypes, this removed lineage-specific TF expression patterns, although such patterns could be observed in the original datasets before normalization (Figure S7). In addition to *trans*-acting TFs, *cis*-regulatory alterations, e.g., involving enhancer hijacking, would also be at work within metastatic tumors.^23^

### Somatic mutation and copy gain events underlie metastasis subtypes

**Figure 5:**
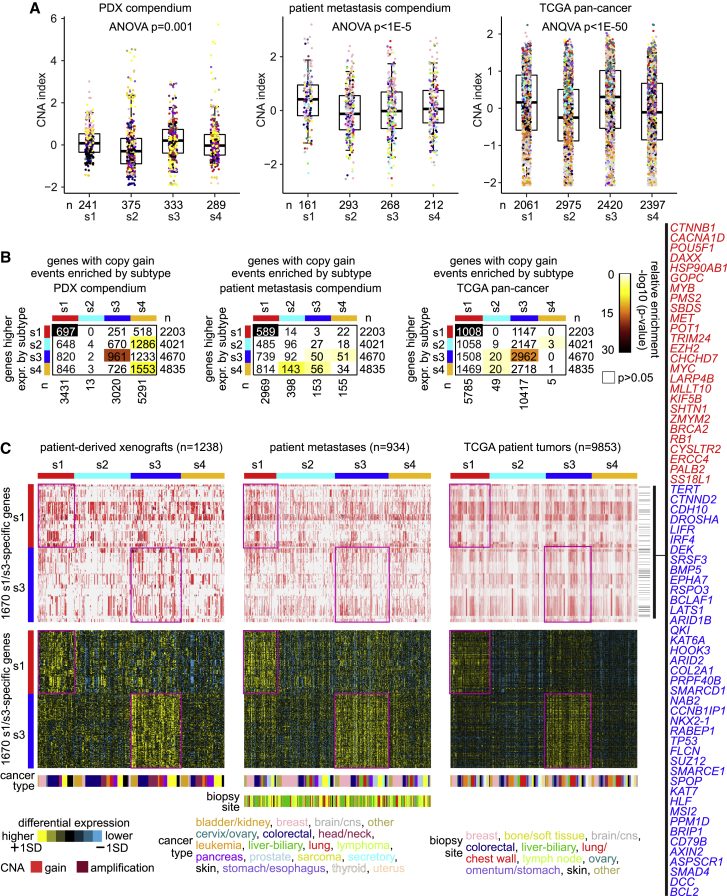
Copy number alteration (CNA) events underlying the molecular subtypes of metastasis (A) For PDX compendium, patient metastasis compendium, and TCGA datasets, overall CNA burden index (standard deviation of CNA values across all genes, centered within each dataset to standard deviations from the median across samples) by PDX-based molecular subtype. Boxplots represent 5% (lower whisker), 25% (lower box), 50% (median), 75% (upper box), and 95% (upper whisker). (B) For PDX compendium, patient metastasis compendium, and TCGA datasets, significance of overlap between the genes high within each of the PDX-based subtypes (using t test, p < 0.01, based on analysis of PDX compendium) and the genes with copy gain events more frequent within each subtype (p < 0.01, one-sided Fisher’s exact test, PDX and patient metastasis datasets; p < 0.001, chi-square test, TCGA dataset). Overlap p values were found by chi-square test. A set of 1,670 genes involves significant gene set overlaps between expression differences and copy gain enrichment patterns for the same subtype, for at least two of the three copy number datasets examined, these significant overlaps involving s1 and s3 subtypes. (C) Taking the set of 1,670 genes noted in (B), involving significant gene set overlaps between expression differences and copy gain enrichment patterns for s1 and s3 subtypes, copy gain patterns and differential expression patterns are represented in PDX compendium, patient metastasis compendium, and TCGA datasets. CNA, copy number alteration; gain, estimated gene copy number between 3 and 5; amplification, estimated gene copy number >5. Example genes listed (red, s1 genes; blue, s3 genes) are well-established cancer-associated genes by COSMIC.^24^

We explored subtype-specific expression differences involving gene copy number alteration (CNA) patterns. Of the 2,405 tumors in our patient metastasis compendium expression dataset, 934 had corresponding gene copy and somatic mutation information, as did 1,238 of the 2,371 tumors in the PDX expression compendium dataset. Consistent with previous observations involving s1 subtype analogs,^16^ s1 tumors showed higher levels of CNA burden relative to s2 and s4 subtypes, as observed across the PDX compendium, the patient tumor metastasis compendium, and TCGA pan-cancer datasets (Figure 5A). In PDX compendium and TCGA datasets, s3 tumors also showed higher overall CNA burden. We also examined small somatic mutation events (single-nucleotide variants and insertions/deletions) for 102 cancer-associated genes in core oncogenic and tumor-suppressive pathways (Table S6).^8^^,^^16^ For all three datasets surveyed (PDX compendium, patient tumor metastasis compendium, TCGA), one gene, *APC*, was consistently enriched for mutation events (p < 0.01, one-sided Fisher’s exact test) in the s1 subtype, due in part to the relative enrichment of s1 for colorectal cancers (Figure S2F). *TP53* was also enriched for mutation events in the s1 subtype (p < 0.01) but for only the patient tumor metastasis and TCGA datasets. For the s2 subtype, six genes—*MTOR*, *PIK3CA*, *PTEN*, *BRAF*, *HRAS*, *KRAS*—were enriched for mutation events (p < 0.01) for exactly two of the three datasets surveyed.

For each gene represented in our compendium datasets, we assessed the significance of the enrichment of copy gain events within each molecular subtype. For the s1 and s3 subtypes, we identified significant gene set overlaps between genes with enriched copy gain events within a given subtype and genes highly expressed within the same subtype (based on the PDX compendium dataset). This pattern was consistent for all three datasets surveyed (PDX compendium, patient tumor metastasis compendium, TCGA; Figure 5B and Table S6). A set of 1,670 genes involved significant gene overlap between expression differences and copy gain enrichment patterns for either s1 or s3 subtypes for at least two of the three copy number datasets examined (Figure 5C). These 1,670 genes involved well-established cancer-related genes,^24^ including *MYC*, *MYB*, *BRCA2*, *ERCC4*, and *MET* for s1 subtype and *TERT*, *BCL2*, and *SUZ12* for s3 subtype. As observed elsewhere,^7^ copy alteration patterns may involve single-copy gains for known oncogenes and single-copy losses for tumor suppressor genes. Here, the above enrichment patterns mostly involve gene copy gain as opposed to gene amplification, and none of the genes in the patterns of gene set overlap exhibited high-level amplification akin to the HER2 gene in breast cancer.

### Pathways represented by metastasis subtypes

**Figure 6:**
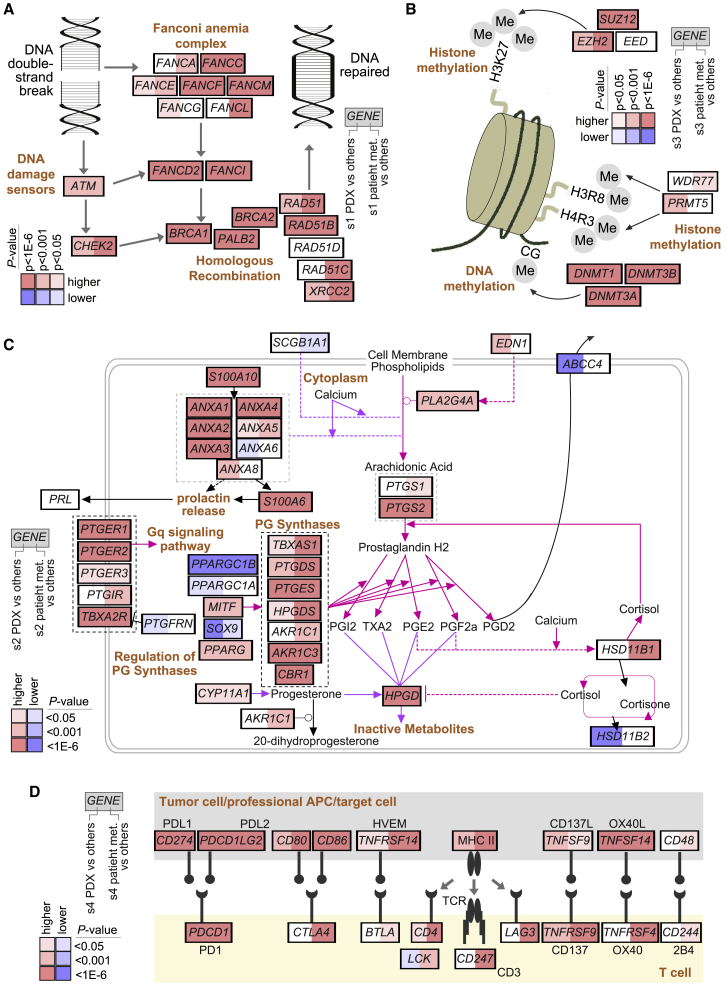
Pathways associated with the molecular subtypes of metastasis (A) Diagram of key genes involved in the DNA double-strand-break repair pathway,^25^ with differential expression patterns represented in both PDX and patient tumor metastasis compendiums, comparing s1 with the rest of the tumors (red, significantly higher in s1). (B) Diagram of key genes involved in methylation of DNA and histones,^25^ with differential expression patterns represented in both PDX and patient tumor metastasis compendiums, comparing s3 with the rest of the tumors (red, significantly higher in s3). (C) Diagram of prostaglandin synthesis and regulation pathway,^26^ with differential expression patterns represented in both PDX and patient tumor metastasis compendiums, comparing s2 with the rest of the tumors (red, significantly higher in s2). (D) Diagram of immune checkpoint pathway (featuring interactions between T cells and antigen-presenting cells, including tumor cells),^8^ with differential expression patterns represented in both PDX and patient tumor metastasis compendiums, comparing s4 with the rest of the tumors (red, significantly higher in s4). The p values in (A)–(D) were found by t test.

Some of the above findings suggested the involvement of key pathways of interest underlying each metastasis subtype (Figures 6 and S6). The enrichment for DNA repair genes within genes higher in s1 tumors (Figure 1D and Table S3) and the higher CNA burden in s1 tumors (Figure 5A) suggested associations involving the DNA double-strand-break repair pathway and Fanconi anemia. These associations were evident when examining key individual genes, including *BRCA1*, *BRCA2*, *FANCD2*, *FANCI*, and *RAD51* (Figure 6A). Many of the genes in this pathway were also higher in s3 tumors, which subtype similarly involved enrichment patterns for DNA repair genes (Figures 1D and S6). The observed association of EZH2 transcription targets with s3 tumors (Figure 4B) and of *SUZ12* copy gain events in s3 tumors (Figure 5C) suggested processes of histone methylation and DNA methylation, with genes higher in s3 also including *DNMT1*, *DNMT3B*, and *DNMT3A* (Figure 6B).

Regarding s2 tumors, when taking the set of genes higher (p < 0.01, t test) in s2 versus other tumors for both PDX and patient metastases compendium expression datasets, enriched wikiPathways^26^ (p < 0.00005, one-sided Fisher’s exact test) included the prostaglandin synthesis and regulation pathway (Figure 6C). This association included annexin genes, *S100A10*, *S100A6*, *PTGER1*, *PTGER2*, *PTGS2*, *TBXA2R*, *AKR1C3*, and *HPGD*. Prostaglandin E2 (PGE2) can promote tumor growth by binding to its receptors and activating signaling pathways that control cell proliferation, migration, apoptosis, or angiogenesis.^27^ Regarding s4 tumors, immune system-related genes were enriched in genes higher in s4 versus other tumors (Figure 1D). Along these lines, the s4 subtype had higher expression of several genes in the immune checkpoint pathway, representing potential targets for immunotherapy,^15^^,^^28^ including *PDCD1* (PD1), *CD274* (PDL1), and *PDCD1LG2* (PDL2) (Figure 6D). Consistent with our understanding of the PDX model, genes in the immune checkpoint pathway with specific roles in the T cells interacting with the antigen-presenting cells were significantly higher in s4 tumors from patient metastases but not from PDXs (Figures 6D and S6). Several genes—including *CD247* (CD3), *CTLA4* (CD152), *TNFRSF4* (CD134), *LAG3*, and T cell marker *LCK*—were significantly higher (p < 0.001, t test) in s4 tumors from patient metastases but not significantly (p > 0.05) in s4 PDX tumors. These differential expression patterns would be consistent with the scenario of tumor cells with antigen presentation interacting with T cells in human tumors, where PDX tumors would not represent the T cell component.

### Associations of metastasis subtypes with drug responses in cancer cell lines

**Figure 7:**
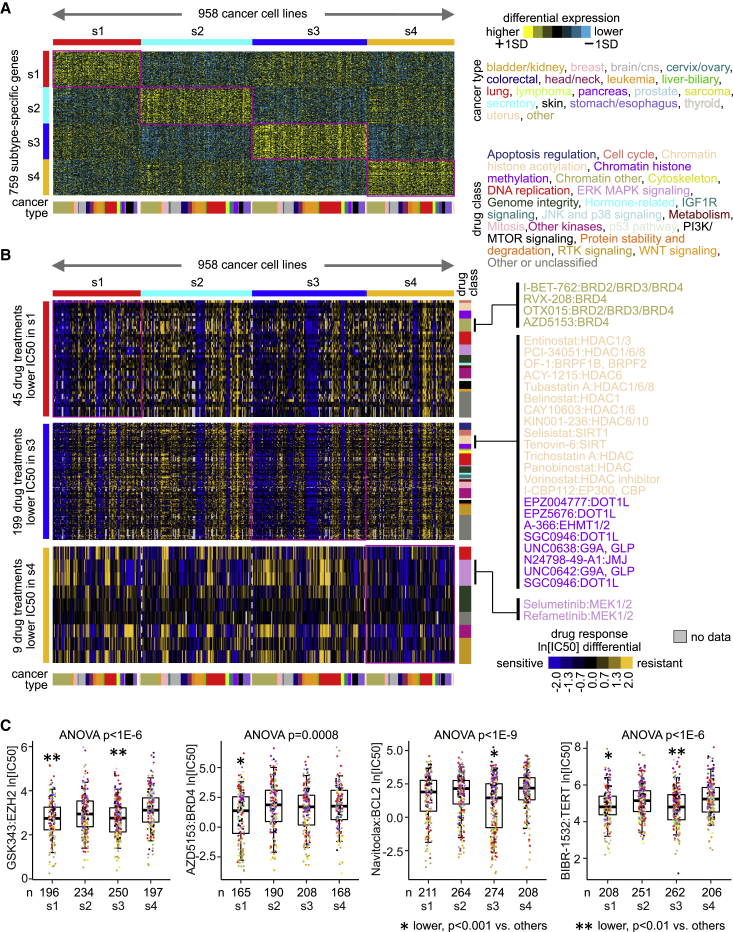
Molecular subtype associations with drug response in cancer cell lines (A) Transcriptional profiles of 958 cancer cell lines represented in the Genomics of Drug Sensitivity in Cancer (GDSC)^29^ dataset (profiles being normalized within their respective cancer type) were classified according to PDX-based molecular subtype. Expression patterns in cell lines for the top set of 800 mRNAs distinguishing between the four PDX-based subtypes (from Figure 1A) are shown (759 of the 800 genes being represented in GDSC). (B) From the GDSC cell lines classified according to metastasis subtype, drug compound treatments with decreases in half-maximal inhibitory concentration (IC_50_) associated with s1, s3, or s4 subtypes (p < 0.001, comparing cell lines of the given subtype with the rest of the cell lines, t test on natural log-transformed IC_50_ values). Selected drug compounds are listed by name. (C) For selected drug compounds, natural log IC_50_s by molecular subtype. Boxplots represent 5% (lower whisker), 25% (lower box), 50% (median), 75% (upper box), and 95% (upper whisker). Data points are colored according to cancer type, according to color coding in (A).

Integrating molecular data on cancer cell lines with their responses to anticancer drugs can identify therapeutic options for cancer subsets.^29^ Similar to the above external expression datasets, we assigned transcriptomic profiles for each of 958 cell lines in the Genomics of Drug Sensitivity in Cancer (GDSC) dataset to a metastasis subtype (Figure 7A). PDX-based subtypes were reflected in cancer cell lines at both the mRNA and the protein levels (Figures S4E and S4F). For each of 544 drug compound treatments with half-maximal inhibitory concentration (IC_50_) measurements, we compared IC_50_ values for cell lines of a given subtype with the rest of the cell lines. Widespread associations of molecular subtype with drug response, well exceeding chance expected, were found for s1, s3, and s4 subtypes (Figure 7B and Table S7). At p < 0.001 significance level (t test on natural log values), 45, 199, and 9 drug treatments showed greater sensitivity levels in s1, s3, and s4 cell lines, respectively. These drug response associations aligned with the above molecular observations involving the subtypes. For example, s1 subtype associated with response to several bromodomain inhibitors (Figures 7B and 7C), conceivably related to this subtype’s association with MYC.^30^ Several drugs targeting chromatin histone acetylation or methylation associated with response in s3 cell lines (Figure 7B). The one drug in GDSC targeting EZH2 showed sensitivity in s3 as well as s1 cell lines (Figure 7C). With s3 involving both *BCL2* overexpression and copy gain (Figure 5C), s3 also associated with greater sensitivity (p < 0.01) to all six BCL2 inhibitors represented in GDSC (Figure 7C and Table S7). With s3 also involving both *TERT* overexpression and copy gain (Figure 5C), s3 associated here with greater sensitivity to TERT inhibition (Figure 5C). Previous studies involving tumor xenografts demonstrate how GDSC IC_50_ values would translate into substantial anticancer effects *in vivo*. For example, BRD4 inhibitors impact tumor growth of s1 cell lines MDA-MB-231 and MDA-MB-468,^31^ BCL2 inhibitors impact tumor growth of s3 cell line OVCAR8,^32^ and telomerase inhibitor impacts tumor growth of s3 cell line HeLa.^33^

## Discussion

By transcriptomics, our study uncovered four major pan-cancer molecular subtypes of metastases. The s1 subtype had extensive copy alterations, higher expression of genes involved in DNA double-strand-break repair, higher expression of TF genes such as *MYC* with corresponding higher expression of their transcriptional target genes, and associations with bromodomain inhibitor response. The s2 subtype had higher expression of genes involved in metabolism and prostaglandin synthesis and regulation. The s3 subtype had higher expression of DNA and histone methylation genes, higher expression of EZH2 and associated transcriptional targets, higher expression of neuroendocrine marker genes and evidence of a type of neuronal differentiation, and higher expression and copy gain of *BCL2* coupled with BCL2 inhibitor response associations. The s4 subtype had higher expression of immune checkpoint and Notch pathway genes. These subtypes were manifested in primary and metastatic tumors, consistent with the notion that metastasis-associated transcriptional programs may be encoded within primary tumors.^34^ By our analytical approach, the molecular subtypes spanned tumors of diverse lineages and tissues of origin and multiple datasets from independent laboratories. The idea that cancer metastases can be categorized into a handful of distinct groups would have important implications for understanding the biology of metastasis. For example, different processes and pathways that appear coordinately manifested in a cancer subtype might suggest a degree of cooperation between these that could be explored further.

The metastasis subtypes reflected expression differences from paired primaries, with subtype switching being common. Metastatic cells that escape from the primary tumor may develop into tumors of a different molecular subtype from that of the primary, while still falling within one of a discrete set of subtypes. When considering the subset of patient tumor metastasis according to a particular subtype, we could observe widespread paired differences between metastases and paired primary, these differences spanning multiple cancer types. Many previous studies (e.g., those from which we incorporated data into the present study) have sought to define global expression differences between metastases and paired primaries for a given cancer type. These previous studies may not have considered the site of metastasis biopsy as a confounding variable, with expression differences largely reflecting differences between tissues of the primary site versus other tissues. Our PDX-based approaches to molecular subtyping circumvented this issue. In addition to global expression differences observed across all metastases versus primary tumors, consistent differences involving only a subset of patients may be considered, as done in our study. For molecular subtypes associated with higher overall levels of CNA, gene copy gain events that increase expression of genes underlying the subtype may be evolutionarily favored. These events might involve a gain of just one or two copies versus the high-level amplification events favoring strong cancer driver genes.

Our molecular subtypes could have important implications for applying existing therapies or developing alternate therapeutic approaches. Therapies potentially targeting subtypes would be represented in our results utilizing cell-line drug responses. MYC oncogene represents a candidate driver of the s1 subtype. While MYC had been traditionally regarded as undruggable, in recent years compounds directly or indirectly inhibiting MYC have shown anticancer activity preclinically, with some of these being developed for clinical trial evaluation.^35^ Consistent with our study’s drug response associations, therapeutic approaches for bromodomain inhibition in cancers characterized by MYC activation are being explored.^30^ Our s2 subtype showed coordinate expression of several genes in the prostaglandin synthesis pathway. COX-2-derived PGE2 supports epithelial tumor aggressiveness by several mechanisms,^36^ and COX-2 selective inhibitors have been explored as a drug for cancer prevention and treatment and found to decrease the incidence of certain malignancies.^27^ Regarding the s3 subtype, there is high interest in targeting EZH2 for cancer therapy. Different types of EZH2 inhibitors are under evaluation in ongoing clinical trials involving different cancer types.^37^ Based on our results, BCL2 inhibitors may also target the s3 subtype. Regarding the s4 subtype, the recent clinical success of immune checkpoint inhibitors created a class of anticancer drugs to treat various malignancies.^38^ One of the current challenges in cancer immunotherapy is developing biomarker panels that distinguish likely responders from non-responders, as markers such as PD-L1 represent continuous rather than discrete variables.^39^ Our findings suggest that no single therapeutic approach would be effective for all cancers but that the gene expression profile and associated molecular subtype of the tumor could help maximize precision medicine approaches.

### Limitations of the study

The number of discoverable pan-cancer subtypes would depend in part on the datasets examined and the analytical approaches used. Future studies examining additional datasets and cancer types might uncover additional subtypes. Subtypes of metastases within a given tissue-based cancer type could also be explored, where subtypes uniquely applicable to a given tissue of origin might be found. This study does not establish our pan-cancer subtypes as directly related to the actual processes of metastasis. The metastatic cascade represents a multistep process, with each step being explored in other studies using suitably tailored experimental model systems. In isolation, our PDX-based pan-cancer subtypes would not fully capture the influence of the tumor microenvironment on cancer metastases. The results of our study represent subtype-specific associations made across different datasets, and reported associations could be considered robust when spanning multiple molecular modalities and datasets. Still, additional directed functional experiments might be needed to establish a particular association of interest more firmly or to gain more insight.

## STAR★Methods

### Key resources table

**Table:** 

REAGENT or RESOURCE	SOURCE	IDENTIFIER
**Deposited data**		

POG570 mutation and expression datasets	Canada’s Michael Smith Genome Sciences Center (GSC) at BC Cancer	http://bcgsc.ca/downloads/POG570/
MET500 mutation and expression datasets	University of Michigan	https://met500.med.umich.edu/datasets
Count Me In (CMI): The Metastatic Breast Cancer (MBC) Project expression datasets	Genome Data Commons (GDC)	https://portal.gdc.cancer.gov/projects/CMI-MBC
GEO patient metastases expression datasets	Gene Expression Omnibus (GEO)	GEO: GSE12630, GEO: GSE14378, GEO: GSE18549, GEO: GSE23629, GEO: GSE29650, GEO: GSE50493, GEO: GSE50760, GEO: GSE60464, GEO: GSE60542, GEO: GSE63668, GEO: GSE74685, GEO: GSE125989, GEO: GSE126078, GEO: GSE131418, GEO: GSE133296, GEO: GSE136037, GEO: GSE137237, GEO: GSE147322, GEO: GSE151580, GEO: GSE159216, GEO: GSE184869
NIH-NCI PDX Development and Trial Centers Research Network (PDXNet)/NCI Patient-Derived Models Repository (PDMR) mutation and expression datasets	National Cancer Institute (NCI)	https://doi.org/10.6084/m9.figshare.14390408
GEO patient-derived xenograft (PDX) expression datasets	Gene Expression Omnibus (GEO)	GEO: GSE76402, GEO: GSE98708, GEO: GSE103340, GEO: GSE118942, GEO: GSE128459, GEO: GSE129127, GEO: GSE130160, GEO: GSE146661, GEO: GSE151343, GEO: GSE157494, GEO: GSE159702, GEO: GSE180790, GEO: GSE181374, GEO: GSE193500
The Cancer Genome Atlas (TCGA) mutation and expression datasets	Broad Institute	https://gdac.broadinstitute.org/
FANTOM5 cell and tissue expression dataset	RIKEN	http://fantom.gsc.riken.jp/5/data/
ENCODE Transcription Factor (TF) binding	Ensembl Biomart (GRCh37/hg19 build)	https://grch37.ensembl.org/info/data/biomart/index.html
Gene expression profiles of 400 siRNA knocked down on HUVEC	Gene Expression Omnibus (GEO)	GEO: GSE27869
Genomics of Drug Sensitivity in Cancer (GDSC) cell line datasets	Wellcome Sanger Institute	https://www.cancerrxgene.org/

**Software and algorithms**		

ConsensusClusterPlus (v3.16)	Bioconductor	https://www.bioconductor.org/packages/release/bioc/html/ConsensusClusterPlus.html
SigTerms (v1.0)	Baylor College of Medicine	https://sigterms.sourceforge.net/

### Resource availability

#### Lead contact

Further information and requests for resources and reagents should be directed to and will be fulfilled by the lead contact, Chad J. Creighton (creighto@bcm.edu).

#### Materials availability

This study did not generate new, unique reagents.

### Experimental model and subject details

#### Human data

Regarding human subjects, cancer molecular profiling data were generated through informed consent as part of previously published studies and analyzed in accordance with each original study’s data use guidelines and restrictions.

#### Cell line data

Molecular and phenotypic data on cell lines was accessed from public repositories, as described below. Details on cell maintenance and care are described in the original studies generating these data.

#### PDX data

Molecular data on PDX models was accessed from previously published studies, as described below. Details on PDX tumor generation and molecular characterization are described in the original studies generating these data.

### Method details

#### Patient tumor metastasis compendium datasets

We assembled a compendium dataset of gene expression profiling data of metastatic tumors from 26 major cancer types (based on tissue of origin) and 24 individual studies (Table S1).^5^^,^^6^^,^^40^^,^^41^^,^^42^^,^^43^^,^^44^^,^^45^^,^^46^^,^^47^^,^^48^^,^^49^^,^^50^^,^^51^^,^^52^^,^^53^^,^^54^^,^^55^^,^^56^^,^^57^^,^^58^^,^^59^ The 2405 tumors in the compendium represented 2158 patients. The above studies analyzed the tumors using global mRNA profiling by RNA-sequencing (RNA-seq) or array platform. We obtained processed expression data tables from the Gene Expression Omnibus (GEO, accession numbers included in Table S1), from websites associated with the study publication (for MET500 and POG570 datasets), or from the Genome Data Commons (in the case of the Count Me In, or CMI dataset). Where multiple studies and associated datasets came from the same research team,^49^^,^^51^ we ensured that tumors were not represented more than once in the compendium, removing duplicate profiles. For RNA-seq data with raw counts data provided, we converted these to Transcripts per million (TPM) expression values. Where we observed considerable variability in the total expression values across genes among profiles, we applied quantile normalization to the individual dataset.^60^ For a given dataset, in instances where genes were represented by more than one feature, the feature with the highest variability across profiles (by standard deviation applied to log2-transformed expression values) was selected to represent the gene in the compendium dataset. Without correction, widespread differences in relative gene levels observed between any two datasets would represent a combination of technical batch effects (e.g., stemming from different mRNA profiling platforms and different laboratories) and of biological differences involving tumor tissue of origin or metastasis biopsy site. To correct for both of the above, we normalized the genes within each dataset and within each cancer type (for those datasets with more than one cancer type represented) to standard deviations from the median, using log2-transformed values, similar to what we have done in previous studies.^8^^,^^15^^,^^16^^,^^17^ This data transformation to unitless standard deviations from the median allowed for the values for a given gene to be comparable among the various datasets. As the set of genes represented in the patient tumor metastasis compendium datasets, we took the 18319 genes represented by Entrez identifier in both the MET500 and POG570 datasets (representing 934 of the 2405 tumor metastases).

Of the 2405 tumors in the above metastasis compendium expression dataset, 307 patient tumor metastases had a corresponding primary tumor pair from the same patient also being profiled. These 307 tumor metastases represented 291 patients, eight major cancer types (based on tissue of origin), and 13 studies. To carry out paired metastasis versus primary tumor comparisons, we compiled a separate metastasis compendium of the above 307 tumor metastases. To normalize the metastases profiles relative to the paired primary, we first centered log2-transformed expression values for each metastasis expression profile on its primary pair, setting the values for the primary pair to zero. Then, within each study dataset, the centered expression values were divided by the standard deviation across the centered metastasis and primary profiles. This normalization step rendered the differential expression values unitless, thereby correcting for inter-dataset differences.

Of the 2405 tumors in our patient tumor metastasis compendium dataset, 934—involving the MET500^5^ and POG570^6^ datasets—had DNA sequencing data yielding gene copy and small somatic mutation information. For the POG570 dataset, gene-level copy values were generated as integers representing the predicted copy number state,^23^ from 0 to 5, 3–5 representing gene copy gain or amplification, correcting for tumor ploidy. For the MET500 dataset, gene-level copy values were generated as integers from 0 to 20, not correcting for tumor ploidy. We first applied a correction for ploidy to each metastatic tumor profile in the MET500 dataset, whereby each gene copy value was divided by the median gene copy value across genes (2 for most tumors) and multiplied by 2. Gene copy values greater than or equal to three for each tumor profile in the MET500 and POG570 datasets were called copy gain, while gene copy values less than two were called as copy loss.

#### PDX expression datasets

Analogous to the above involving the patient metastasis expression compendium dataset, we assembled a compendium dataset of PDX tumors representing over 18 major cancer types (based on tissue of origin) and 14 individual studies (Table S1).^11^^,^^12^^,^^61^^,^^62^^,^^63^^,^^64^^,^^65^^,^^66^^,^^67^^,^^68^^,^^69^^,^^70^^,^^71^ We identified the above studies and associated expression datasets by searching the GEO database. In addition, we incorporated an expression profiling dataset from Sun et al.^72^ representing 1551 PDX tumors and 536 patients and over 16 major cancer types by tissue of origin, involving the NIH-NCI PDX Development and Trial Centers Research Network (PDXNet) and the NIH-NCI Patient-Derived Models Repository (PDMR) repositories. The above studies analyzed the tumors using global mRNA profiling by RNA-sequencing (RNA-seq) or array platform. For RNA-seq data with raw counts data provided, we converted these to Transcripts per million (TPM) expression values. Where we observed considerable variability in the total expression values across genes among profiles, we applied quantile normalization to the individual dataset.^60^ For a given dataset, in instances where genes were represented by more than one feature, the feature with the highest variability across profiles (by standard deviation applied to log2-transformed expression values) was selected to represent the gene in the compendium dataset. As carried out above for the 2405-patient tumor metastases compendium, we normalized the genes within each dataset and within each cancer type (for those datasets with more than one cancer type represented) to standard deviations from the median, using log2-transformed values. We obtained the PDXNet/PDMR expression data matrix (TPM values) from the Sun et al. publication. We transformed log2-transformed expression values within each cancer type to standard deviations from the median to remove tissue-dominant differences. The set of 18319 genes represented in the patient tumor compendium dataset was the set represented in the PDX compendium dataset.

PDXs may be vulnerable to lymphomagenesis.^73^ Therefore, we took conservative measures to remove PDX tumor profiles from our compendium that manifested strong patterns associated with lymphocytes. In the original study involving the GSE76402 dataset,^11^ 14 samples were found to be contaminated by murine or human lymphomas and were not further considered in the analysis. Based on our analysis of these 14 sample profiles, we removed sample profiles with either B cell marker CD19 elevated at three standard deviations from the dataset median or a gene signature of B cells from Bindea et al.^74^ elevated at three standard deviations from the dataset median. Our present study does not make any definitive conclusions regarding the samples not included in the study. The final PDX compendium expression dataset consisted of 2371 tumors representing 1000 patients.

#### Pan-cancer molecular subtype discovery

We used the PDX compendium expression dataset to identify molecular subtypes, which we then examined in other expression datasets. ConsensusClusterPlus R-package^75^ (using R version 4.1.1) was used to identify the structure and relationship of the samples. For unsupervised clustering analysis, we randomly selected 2000 genes represented in at least 2300 of the 2371 tumor profiles of the PDX compendium dataset. Consensus ward linkage hierarchical clustering identified k = 2 to k = 15 subtypes, with the stability of the clustering increasing with increasing k. We considered multiple subtype solutions, as described in Figure S2. Beyond a 7-subtype solution, additional subtypes identified involved relatively fewer samples and were not well represented in both GEO and PDXNet/PDMR compendium subsets. In exploring the 7-subtype solution further, three of these subtypes had samples represented almost entirely in either GEO datasets or the PDXNet/PDMR subsets but not both, where we sought robust subtype associations involving multiple datasets. Therefore, we reclassified the profiles in k subtypes 5–7 according to the best fit among subtypes 1–4 to arrive at the final 4-subtype solution (s1 through s4). For this reclassification, we determined the top 200 gene correlates for each of the four subtypes. For each subtype, we assigned either "1" if the gene was a top 200 gene for the given subtype and "0" if otherwise. We then computed the Pearson correlation between each PDX subtype classifier and the sample profiles to be reclassified. We assigned each reclassified tumor profile to one of the four subtypes, based on which subtype classifier showed the highest correlation with the given external dataset profile.

Based on the set of subtypes derived from our PDX compendium expression dataset, we examined expression profiling datasets external to the PDX compendium, classifying each external tumor profile by PDX-based subtype. We classified tumors in the patient tumor metastasis compendium and TCGA pan-cancer datasets by PDX-based subtype. Within each cancer type of TCGA dataset (by TCGA project), we normalized log-transformed mRNAs to standard deviations from the median. As a classifier, we used the top set of 800 genes distinguishing between the PDX-based subtypes based on analysis of the PDX compendium (200 genes for each of the four subtypes, based on all 2371 PDX tumors). To define the top over-expressed genes for each subtype, we first compared PDX tumors of the given subtype with the rest of the tumors by t-test. For a given subtype, a top gene had the highest differential expression by t-statistic compared to the other subtypes and a higher t-statistic than the other genes that did not make the top list. As the classifier for each subtype, we assigned the 800 genes "1" if gene was a top 200 gene for the given subtype and "0" if otherwise. We then computed the Pearson correlation between each external profile and each PDX subtype classifier. We assigned each external tumor profile to a PDX-based subtype, based on which subtype classifier showed the highest correlation with the given external dataset profile. We similarly classified tumor profiles according to our previously identified TCGA-based pan-cancer subtypes8. Taking the previously defined 854 mRNAs distinguishing between TCGA-based subtypes as the subtype classifier, we assigned to each gene "1" or "-1" for up versus down, respectively, if the gene was a top 100 gene for the given subtype and "0" if otherwise. Tumor profiles that did not significantly align with TCGA-based c1 or c3-c10 subtypes with a significance of p < 0.05 (Pearson’s correlation) were assigned to the nondescript "c2" subtype.

For TCGA and cell line datasets, proteomic data by mass spectrometry-based platform or by reverse-phase protein array (RPPA) platform were available. To compare with the results of the mRNA-based classification (these mRNA-based classifications being used for downstream analyses), we classified tumors and cell lines based on available proteomic data, as presented in Figure S4. Log2-transformed protein expression values (by either mass spectrometry-based or RPPA platform) were centered to standard deviations from the median within each cancer type. For the mass spectrometry-based datasets, we used as the classifier the top 800 subtype-specific genes from the PDX dataset (Figure 1A). For the TCGA RPPA dataset, we used as a classifier the set of represented total protein features from which a significant association with a particular subtype was observable in the PDX compendium dataset (p < 0.001 by t-test, based on logged and centered protein expression values).

#### Differential expression analyses

We assessed differential expression between comparison groups using t-tests on expression values log2-transformed and normalized within each dataset and cancer type as described above. Differential gene sets greatly exceeded the estimated chance expected by multiple testing of 18319 genes, using the method of Storey and Tibshirani.^76^ We applied a nominal p-value cutoff to each gene when comparing subtype-specific patterns based on multiple criteria. We used this rather than a stringent false discovery rate cutoff to lower false negative results (while the multiple criteria would keep the false positive rate due to multiple gene testing low). In defining the gene expression signature of metastasis versus paired primary within a given cancer type (Figure S1), we used the compendium of 307 metastasis samples, representing eight cancer types.

#### Gene signature analyses

We surveyed global expression patterns associated with cells and tissues of the CNS, using the public fantom datasets.^20^ We obtained gene expression profiles from various normal human cells and tissues from the FANTOM5 data repository (http://fantom.gsc.riken.jp/5/data/). We removed profiles from fetal or embryonic human specimens from the analysis for our study. We centered log2 expression values for each gene in the fantom dataset on the median of sample profiles. For each fantom differential expression profile (genes centered within the fantom dataset), we took the inter-profile correlation (Pearson’s) with that of the differential expression profile for each PDX and patient tumor metastasis (with the genes in each compendium centered and normalized as described above).

For gene signatures of gene knockdown, we referred to the GSE27869 expression profile dataset of human umbilical vein endothelial cells (HUVECs) transfected with siRNAs for 400 different genes.^22^ We normalized log2 gene expression values in GSE27869 to standard deviations from the median across the 400 profiles. Of the 400 genes represented in GSE27869, 44 involved the 158 TFs surveyed using Encode data (see below). For each siRNA differential expression profile, we took the inter-profile correlation (Pearson’s) with that of the differential expression profile for each PDX and patient tumor metastasis. We then compared the siRNA signature scoring levels among the molecular subtypes.

#### Enrichment analyses for TF bound genes

We obtained TF binding site locations, based on ENCODE consortium data,^21^ from Ensembl (GRCh37/hg19 build). We used TF sites as identified in the HeLa-S3, HepG2, and K562 cell lines (accessed April 2022), involving 158 TFs. We defined associations between TFs and genes as a TF binding site falling within 2kb upstream of the gene start. For each TF and each PDX-based subtype, we identified patterns of significant gene set overlap (by one-sided Fisher’s exact test or chi-square test) between the TF-bound genes and the genes with higher relative expression in the PDX-based subtype relative to other tumors. We separately evaluated the top genes for PDX and patient tumor metastases dataset comparisons (p < 0.01 unpaired t-test, with levels also highest in the given subtype compared to all other subtypes).

#### Comparisons of orthogonal subtype-associated gene sets

We compared subtype-associated gene sets obtained from orthogonal comparisons to identify patterns of significant gene overlap of interest. For each PDX-based subtype, we overlapped the set of genes high within that subtype versus the rest of the PDX tumors with the set of genes high in patient metastases of the same PDX-based subtype versus the corresponding paired primaries. We used a statistic cutoff of p < 0.01 for each gene set (PDX dataset comparisons, unpaired t-test, with levels also being highest in the given subtype compared to all other subtypes; paired patient comparisons, paired t-test). One-sided Fisher’s exact or chi-square tests evaluated the significance of the overlap between the orthogonal gene sets. Analogous comparisons were carried out for the sets of genes low in each PDX-based subtype and the sets of genes lower in patient metastasis of the same subtype versus the corresponding paired primaries.

We also compared differentially expressed genes associated with a given PDX-based subtype with genes with copy gain more frequent in that subtype. Of the 2405 tumors in our patient tumor metastasis compendium dataset, 934—involving the MET500^5^ and POG570^6^ datasets—had gene copy information. Of the 2371 tumors in the PDX expression compendium dataset, 1238—involving the PDXNet/PDMR dataset—had gene copy information. In addition, 9853 tumors in TCGA had both gene copy and expression information. For each dataset, we assessed the frequency of gene copy gain events (i.e., three or more copies) for each gene by subtype, with significance of enrichment by one-sided Fisher’s exact or chi-square test (the latter in instances where large numbers were involved). Differentially expressed genes by subtype were based on comparisons of PDX-based subtype versus the rest of the tumors. For each of the three datasets with gene copy information, chi-square tests evaluated the significance of the overlap between genes with higher expression within a given PDX-based subtype (with levels also being highest in the given subtype compared to all other subtypes) and the genes with copy gain events more frequent within each subtype.

#### Drug response associations

Using the Genomics of Drug Sensitivity in Cancer (GDSC)^29^ resource, we classified 962 cancer cell lines according to PDX-based subtype. Of the 962 cell lines, 214 were annotated as metastatic (Table S1). GDSC expression data and drug compound half maximal inhibitory concentration (IC50) data were downloaded in February 2020 (GDSC1-dataset) and in October 2022 (GDSC2-dataset). We merged the two GDSC IC50 datasets into one. if a drug treatment and cell line were represented in both datasets, we averaged the two values; otherwise, we used whichever IC50 dataset had available data. GDSC IC50 data represented 623 drug treatments involving 544 compounds. Within each cancer type of the GDSC expression array dataset, log base 2-transformed genes were normalized to standard deviations from the median. Using the top 200 genes for each subtype as defined using the PDX compendium expression dataset, we classified the cell lines as described above (based on mRNA data). We further evaluated the cell lines for differences in IC50 drug responses according to molecular subtypes, using t-test on natural log-transformed IC50 values, comparing cell lines of the given subtypes with the rest of the cell lines.

### Quantification and statistical analysis

All p values were two-sided unless otherwise specified. Enrichment of GO annotation terms^77^ within sets of differentially expressed genes was evaluated using SigTerms software^78^ and one-sided Fisher’s exact tests. Visualization using heat maps was performed using both JavaTreeview (version 1.1.6r4)^79^ and matrix2png (version 1.2.1).^80^ Figures indicate exact value of n (number of tumors or cell lines), and the statistical tests used are noted in the Figure legends and next to reported p-values in the results section. Boxplots represent 5%, 25%, 50%, 75%, and 95%. Figures represent biological and not technical replicates.

## Data Availability

• This paper analyzes existing, publicly available data. Details on accessing the datasets are listed in the key resources table. The compendium datasets of gene expression profiles for PDX, patient metastases, and paired patient metastasis with primary—compiled as part of our study—are available through GitHub [https://github.com/chadcreighton/metastasis-expression-compendium]. Each expression dataset is uploaded on GitHub as a series of separate files by individual study, using a common gene feature set with the same ordering across files. One can concatenate the individual matrices together to assemble the compendium datasets using in this study. • This paper does not report original code. No custom computer code was used for data collection, which was performed using open-source software. Additional processing involved in-house scripts that are available upon request. All analyses used previously published software or methods. • Any additional information required to reanalyze the data reported in this paper is available from the lead contact upon request.

## Supplemental information

Document S1. Figures S1–S7

Table S1. Tumor-level sample annotation table, related to Figure 1 Provided as an Excel file. By tumor, assigned subtypes and other information are provided for the PDX compendium dataset, the patient tumor metastases compendium dataset, TCGA dataset, and GDSC cell line dataset. Also included is a list of datasets used for the PDX and patient metastasis compendiums.

Table S2. Gene-level correlations in the paired tumor metastases versus paired primary compendium dataset, related to Figure 2 Provided as an Excel file. For 18,319 genes represented in the compendium datasets, paired metastasis versus primary tumor statistics are provided across all 307 tumors in the compendium, across samples within each cancer type (breast, brain, colorectal, head/neck, cervix/ovary, pancreas, thyroid, kidney), and across samples within each PDX-based molecular subtype.

Table S3. Gene-level correlations in PDX datasets, related to Figure 1 Provided as an Excel file. For 18,319 genes represented in the compendium datasets, subtype-level correlations are provided for the PDX compendium dataset (n = 2,371 tumors). Gene membership in the top 800 genes distinguishing between the subtypes is also provided. Also included are Gene Ontology enrichment results and associated genes, for the top 200 genes most highly expressed within each PDX-based subtype (from Figure 1).

Table S4. Gene-level correlations in patient tumor metastases compendium dataset, related to Figure 1 Provided as an Excel file. For 18,319 genes represented in the compendium datasets, subtype-level correlations are provided.

Table S5. Gene-level associations with transcription factor (TF) binding, related to Figure 3 Provided as an Excel file. For 18,319 genes represented in the compendium datasets, TF binding association (within 2 kb of the gene start) is provided, for each of 158 TFs. TF binding associations were obtained from Ensembl. Also provided are enrichment associations of TF-bound genes within the genes high in each PDX-based subtype, across both PDX compendium and patient tumor metastasis compendium datasets.

Table S6. Gene-level associations with somatic copy number alterations and small mutation, related to Figure 5 Provided as an Excel file. For 18,319 genes represented in the compendium datasets, the numbers of copy amplification/gain events per subtype and the numbers of copy loss/deletion events per subtype are provided, along with the associated enrichment patterns according to subtype. Also provided is gene membership within the top overexpression and copy gain enrichment associations featured in Figure 5. A separate tab provides mutation events by subtype, with associated significance of enrichment for a set of 102 cancer-associated genes in core oncogenic and tumor-suppressive pathways.

Table S7. Cancer cell line associations, related to Figure 7 Provided as an Excel file. For 18,319 genes represented in the compendium datasets, subtype-level correlations according to the Genomics of Drug Sensitivity in Cancer (GDSC) expression dataset are provided. Also provided are the differences by natural log IC_50_ by subtype (t statistics comparing cell lines of the given subtypes with the rest of the cell lines, by t test on natural log-transformed IC_50_ values).

Document S2. Article plus supplemental information
