# Supplementary material for: Pan-cancer molecular subtypes of metastasis reveal distinct and evolving transcriptional programs
Source: Cell Rep Med. 2023 Feb 1;4(2):100932. doi: 10.1016/j.xcrm.2023.100932 (PMC9975284; doi:10.1016/j.xcrm.2023.100932)
Supplement: Document S1. Figures S1–S7 [file mmc1.pdf]

Cell Reports Medicine, Volume *4*

Supplemental information

Pan-cancer molecular subtypes of metastasis reveal distinct and evolving transcriptional programs

Yiqun Zhang, Fengju Chen, and Chad J. Creighton

Supplementary Figures

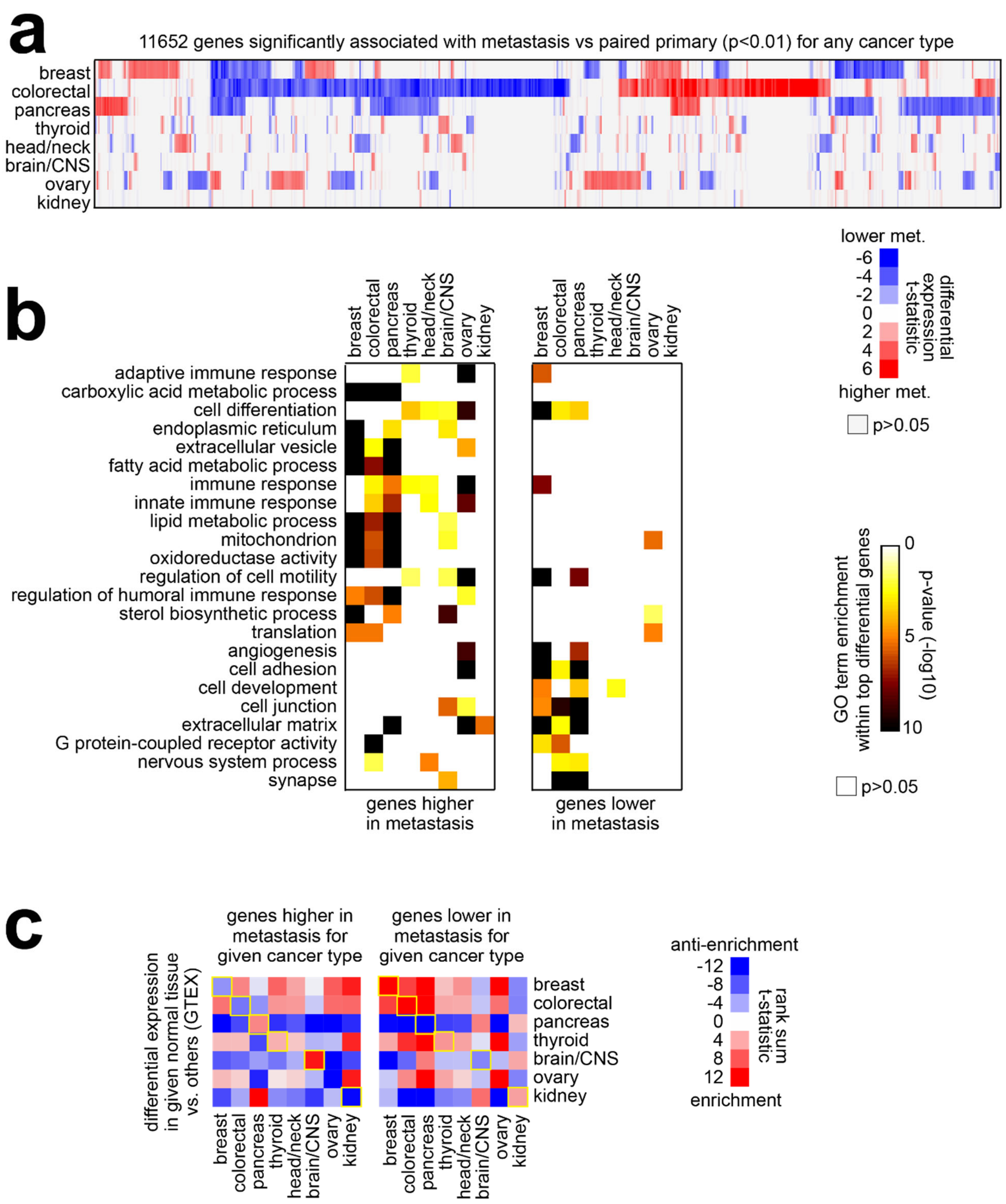

Figure S1. Gene expression signatures of metastasis versus paired primary, by cancer type. Related to STAR Methods. (a) Across eight cancer types represented in our paired metastasis and primary compendium dataset, heat map of differential t-statistics (paired t-test on log-transformed data), by cancer type, comparing metastasis versus paired primary (red, higher expression in metastasis; white, not significant with $p > 0.05$), for 11652 genes significant for any cancer type ($p<0.01$). **(b)** Significance of enrichment (by one-sided Fisher's exact test) for selected GO terms with the respective sets of genes higher or lower in metastasis versus paired primary ($p<0.01$, paired t-test) for each cancer type represented. **(c)** A likely confounder with the above metastasis versus primary expression patterns would involve differences in non-cancer cells between the primary site and the metastasis biopsy site (e.g., comparing breast versus non-breast tissues within the breast cancer dataset). Here, we used the GTEX dataset[1] to compare normal tissues of a given type with the rest of the normal tissue profiles, based on analysis of 11688 tissues. We then evaluated the overall enrichment pattern of each metastasis-associated gene set (from part a, using $p<0.05$) within each GTEX-based normal tissue differential profile (by rank sum statistic). Entries in the correlation matrix corresponding to the same tissue type between GTEX and patient metastasis compendium are highlighted in yellow. For three cancer types (breast, colorectal, and kidney), genes up in metastasis are anti-enriched within the corresponding normal tissues and genes down in metastasis are enriched within normal tissues. With breast cancer, for example, this can reflect that the metastasis sample biopsy has more non-breast cells and less breast cells as compared to the primary sample biopsy. The genes arising from the above metastasis versus primary paired comparisons were not used as the basis for defining our PDX-based molecular subtypes.

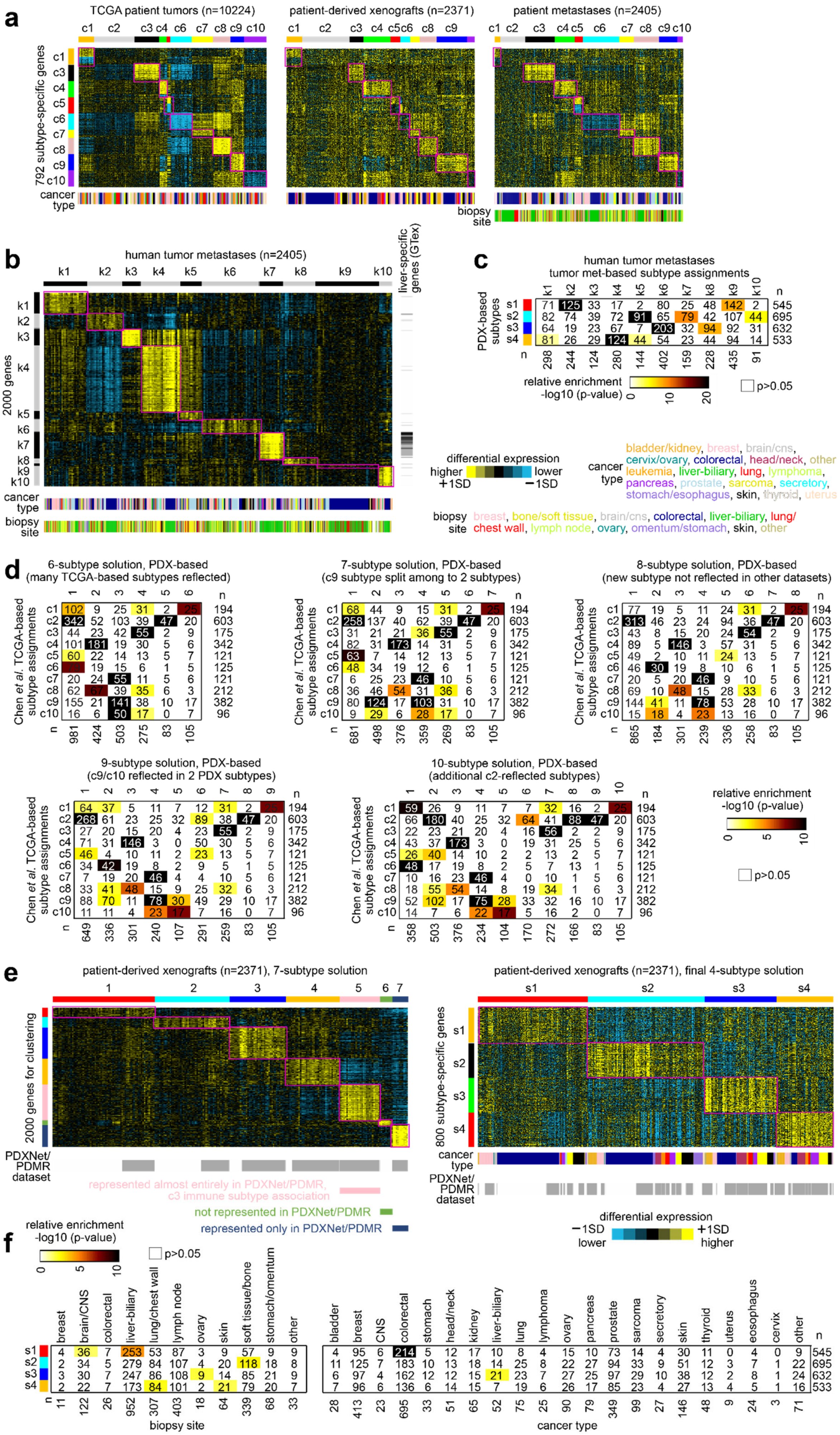
a
TCGA patient tumors (n=10224)
patient-derived xenografts (n=2371)
patient metastases (n=2405)
792 subtype-specific genes
cancer type
biopsy site
b
human tumor metastases (n=2405)
2000 genes
liver-specific genes (GTex)
cancer type
biopsy site
c
human tumor metastases
tumor met-based subtype assignments
PDX-based subtypes
relative enrichment
-log10 (p-value)
p>0.05
differential expression
higher
lower
+1SD
−1SD
cancer type
bladder/kidney, breast, brain/cns, cervix/ovary, colorectal, head/neck, other, leukemia, liver-biliary, lung, lymphoma, pancreas, prostate, sarcoma, secretory, stomach/esophagus, skin, thyroid, uterus
biopsy site
breast, bone/soft tissue, brain/cns, colorectal, liver-biliary, lung/chest wall, lymph node, ovary, omentum/stomach, skin, other
d
6-subtype solution, PDX-based
(many TCGA-based subtypes reflected)
7-subtype solution, PDX-based
(c9 subtype split among to 2 subtypes)
8-subtype solution, PDX-based
(new subtype not reflected in other datasets)
9-subtype solution, PDX-based
(c9/c10 reflected in 2 PDX subtypes)
10-subtype solution, PDX-based
(additional c2-reflected subtypes)
Chen et al. TCGA-based subtype assignments
relative enrichment
-log10 (p-value)
p>0.05
e
patient-derived xenografts (n=2371), 7-subtype solution
2000 genes for clustering
PDXNet/PDMR dataset
represented almost entirely in PDXNet/PDMR, c3 immune subtype association
not represented in PDXNet/PDMR
represented only in PDXNet/PDMR
patient-derived xenografts (n=2371), final 4-subtype solution
800 subtype-specific genes
cancer type
PDXNet/PDMR dataset
differential expression
−1SD
lower
+1SD
higher
f
relative enrichment
-log10 (p-value)
p>0.05
breast
brain/CNS
colorectal
liver-biliary
lung/chest wall
lymph node
ovary
skin
soft tissue/bone
stomach/omentum
other
biopsy site
bladder
breast
CNS
colorectal
stomach
head/neck
kidney
liver-biliary
lung
lymphoma
ovary
pancreas
prostate
sarcoma
secretory
skin
thyroid
uterus
eosophagus
cervix
other
cancer type

Figure S2. Determining the number of pan-cancer metastasis subtypes. (a) Pan-cancer molecular subtyping of tumor metastases based on TCGA-based subtypes. Pan-cancer molecular subtypes were previously defined using The Cancer Genome Atlas (TCGA) datasets[2]. Gene expression profiles of PDX tumors and patient tumor metastases in our compendium datasets were each classified according to TCGA-based subtype c1 through c10. Expression patterns for the previously-defined top set of 854 mRNAs distinguishing between the ten TCGA-based subtypes[2] are shown for TCGA, PDX, and patient tumor metastases datasets. TCGA subtype-specific expression patterns across the datasets are highlighted. SD, standard deviations from the median within a given dataset and within cancer type. See part b for color coding. **(b)** Expression-based subtyping of patient tumor metastases resulted in subtypes that would be confounded with tissue biopsy site. Consensus ward linkage hierarchical clustering identified k = 10 subtypes represented in our patient tumor metastasis compendium of 2405 metastasis samples. Differential expression patterns for the top 2000 most variable genes used in the clustering are represented. Also represented are genes higher ($p<0.05$, t-test on log2-transformed expression data) in normal liver versus other tissues, based on analysis of the GTex dataset[1]. As seen here, the k7 subtype was highly enriched for metastases sampled from the liver and was strongly associated with liver-specific genes. Also, the k9 subtype was enriched ($p<1E-6$, one-sided Fisher's exact test) for samples from lung, and the k3 subtype was enriched for samples from the lymph node. Problematic results such as the above led us to utilize expression data from PDX models in our final analysis, where the contribution of non-cancer cells would be much less of a factor. For expression heat map, SD represents standard deviations from the median within a given cancer type and within a given dataset. **(c)** For the patient tumor metastasis compendium dataset, the significance of overlap between the patient tumor metastasis-based subtype assignments (from part a) and the final PDX-based subtype assignments used in the final study (from main Figure 1) is indicated. P-values by one-sided Fisher's exact test. Some overlap between the respective assignments is observed, though the final PDX-based subtyping results could be considered the cleaner solution that avoided subtypes strongly associated with biopsy site. **(d)** Determination of pan-cancer subtypes using the PDX compendium dataset. Consensus ward linkage hierarchical clustering identified k = 2 to k = 15 subtypes, based on the 2371 tumors in our PDX compendium dataset. The significance of overlap of previously identified pan-cancer subtypes based on TCGA cohort predominantly representing primary cancer[2] (c1-c10, rows) with subtypes obtained from the PDX compendium dataset. Subtype solutions from 6 subtypes to 10 subtypes are represented here. P-values by one-sided Fisher's exact test. We expected perhaps ten or fewer subtypes, based on previous studies[2-4] (given that subtypes representing immune or stroma infiltration would not be represented in the PDX-based subtypes). Beyond a 7-subtype solution, additional subtypes identified did not encapsulate the previous subtypes and were not well represented in both GEO and PDXNet/PDMR compendium subsets. The 7-subtype solution was therefore selected

and explored further below. **(e)** Across the 2371 PDX tumor expression profiles, differential expression patterns for the set of 2000 genes used to define the original subtypes (left) and for another set of 800 genes (right) found to best distinguish between the respective subtypes in the final 4-subtype solution (top ~200 over-expressed mRNAs for each subtype). Expression values are normalized within each given cancer type and each given dataset (SD, standard deviation from the median within a given cancer type within a given dataset). On the left, the 7-subtype solution from consensus ward linkage hierarchical clustering (part c) is considered, where subtypes 5, 6, and 7 are almost entirely represented in either GEO datasets or the PDXNet/PDMR subsets but not both. In contrast, we believed that robust subtype associations should involve multiple datasets. Therefore, we reclassified the profiles in k subtypes 5-7 according to the best fit among subtypes 1-4 to arrive at the final 4-subtype solution (s1 through s4), represented on the right. **(f)** Left, association of molecular subtype (s1-s4) with metastasis biopsy site; right, association of molecular subtype with cancer type by tissue of origin, based on the patient metastasis compendium dataset. Overall, strong associations are not observed here, except for s1 subtype significantly associating with colorectal tumors (though not exclusive to this cancer type). Of the 695 colorectal metastasis samples in the patient metastasis compendium, 586 were biopsied from the liver (Data File S1). The enrichment of s1 for colorectal tumors would therefore largely explain the more moderate association of s1 with liver biopsy site. Related to Figure 1.

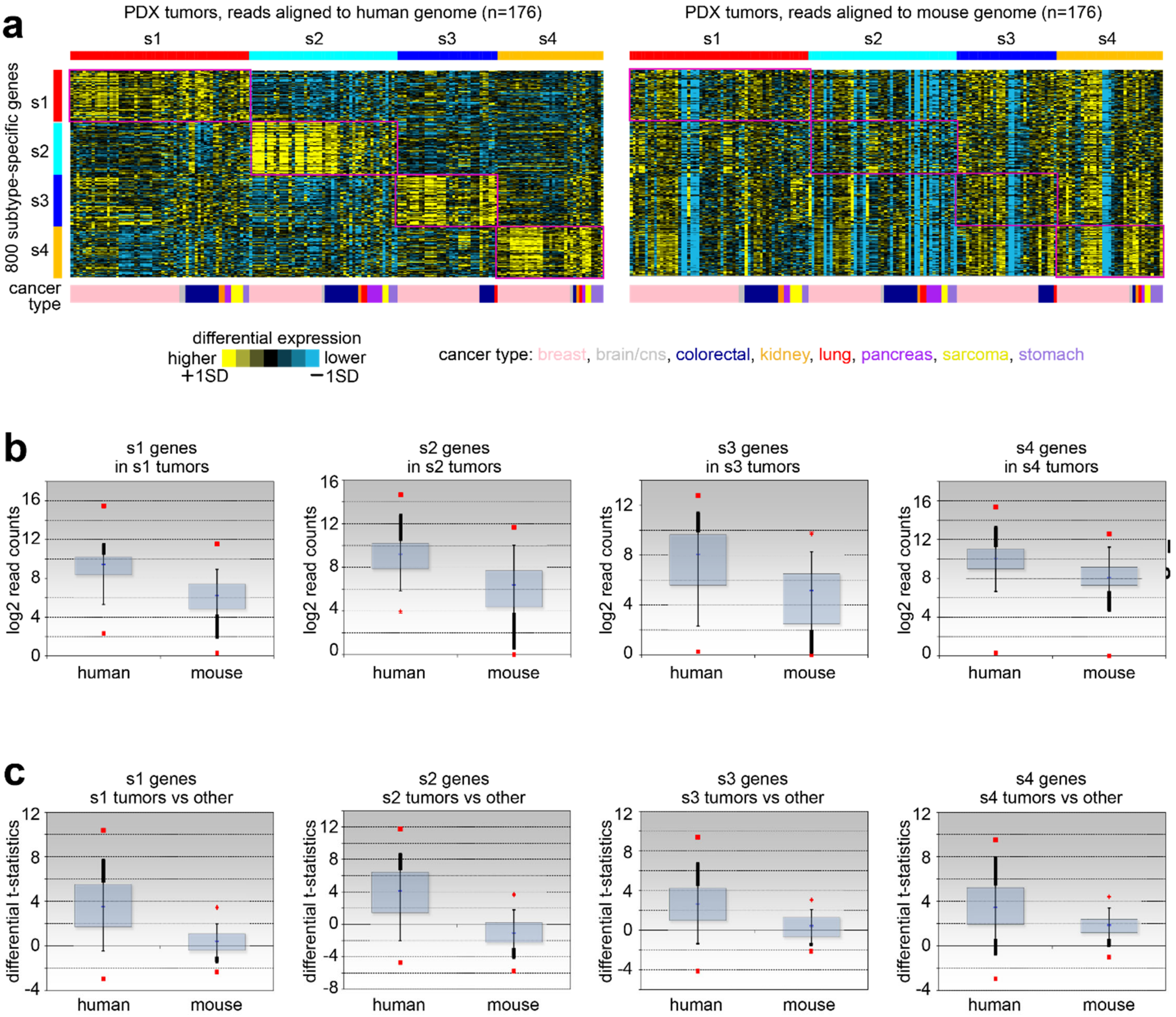

Figure S3. Differential expression patterns associated with PDX-based molecular subtypes are attributable to the cancer cells, not the host. (a) Sequencing reads were aligned to both human and mouse genomes for two of the RNA-seq datasets represented in our PDX compendium (GSE118942[5] and GSE159702[6]). For these two datasets, expression patterns for the top set of 800 subtype-specific mRNAs distinguishing between the four PDX-based subtypes (from Figure 1a) are shown for both the expression data from human genome alignments (left) and the expression data from mouse genome alignments (right). The subtype-specific differential expression patterns are observed here in the version of the dataset based on human genome alignments but not in the version based on mouse genome alignments, indicating that the patterns are specific to the human cells of the tumor and not the mouse cells of the host.

(b) For each of the four subtypes, boxplots of the log2 read counts for the top 100 associated genes (from Figure 1a) based on human versus mouse alignments. On average, the log2 read counts are much higher for human than for mouse. **(c)** For each of the four subtypes, boxplots of the differential t-statistics (comparing for each subtype the average expression levels versus the rest of the tumors by t-test) for the top 100 associated genes (from Figure 1a) based on human versus mouse alignments. Consistent with the heat map representation in part a, the results from the human alignments but not the results from the mouse alignments show statistical significance within the GSE118942/GSE159702 combined dataset. Box plots represent 5% (lower whisker), 25% (lower box), 50% (median), 75% (upper box), and 95% (upper whisker). The above results based on RNA-seq alignments are consistent with analogous experiments previously carried out using expression arrays[7,8], which would be relevant for the expression datasets in the PDX compendium based on array platform. Related to Figure 1.

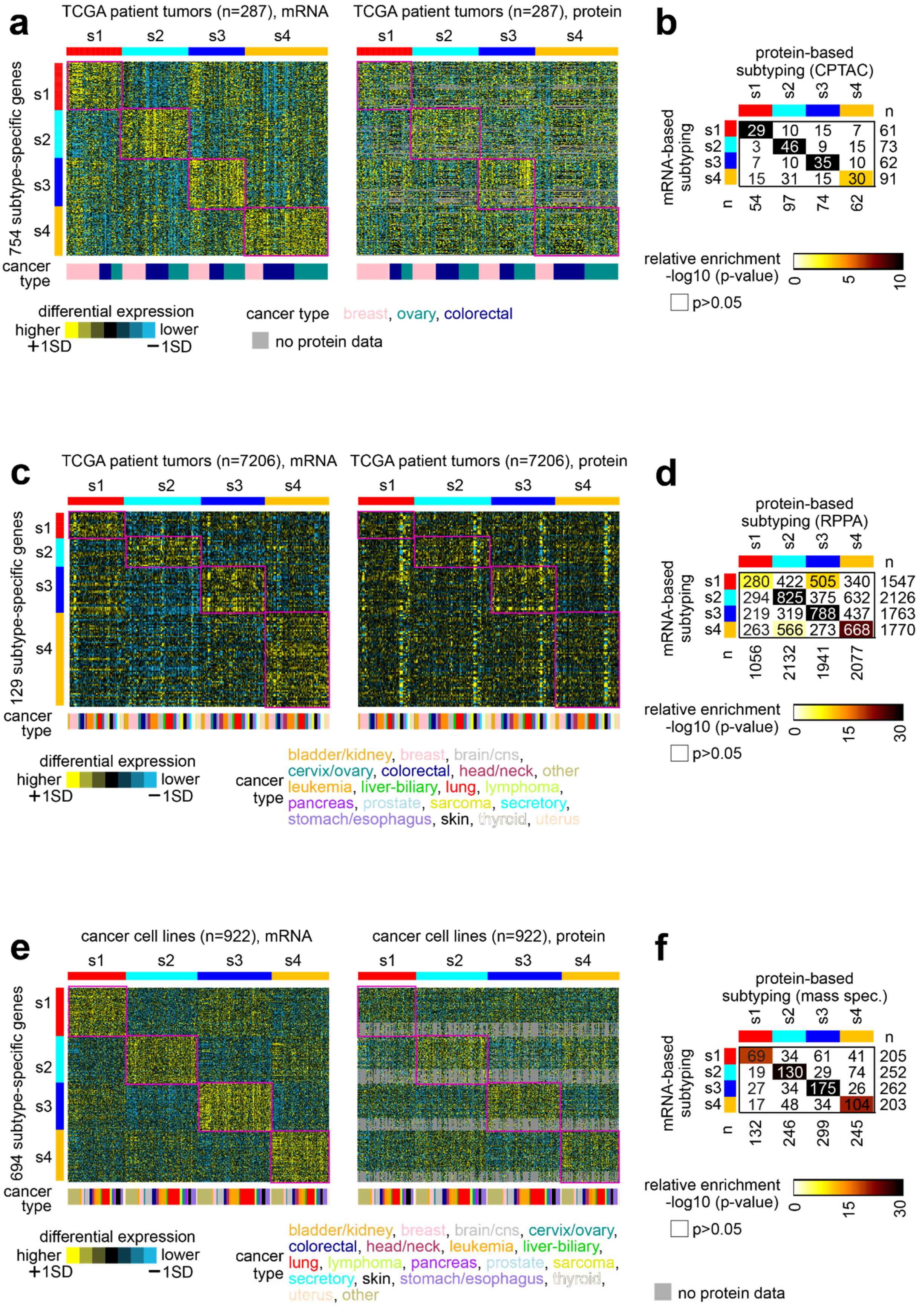
a
TCGA patient tumors (n=287), mRNA
TCGA patient tumors (n=287), protein
s1 s2 s3 s4
754 subtype-specific genes
cancer type
differential expression
higher +1SD
lower −1SD
cancer type breast, ovary, colorectal
no protein data
b
protein-based subtyping (CPTAC)
mRNA-based subtyping
s1 29 10 15 7 61
s2 3 46 9 15 73
s3 7 10 35 10 62
s4 15 31 15 30 91
n 54 97 74 62
relative enrichment -log10 (p-value)
0 5 10
p>0.05
c
TCGA patient tumors (n=7206), mRNA
TCGA patient tumors (n=7206), protein
s1 s2 s3 s4
129 subtype-specific genes
cancer type
differential expression
higher +1SD
lower −1SD
cancer type bladder/kidney, breast, brain/cns, cervix/ovary, colorectal, head/neck, other leukemia, liver-biliary, lung, lymphoma, pancreas, prostate, sarcoma, secretory, stomach/esophagus, skin, thyroid, uterus
d
protein-based subtyping (RPPA)
mRNA-based subtyping
s1 280 422 505 340 1547
s2 294 825 375 632 2126
s3 219 319 788 437 1763
s4 263 566 273 668 1770
n 1056 2132 1941 2077
relative enrichment -log10 (p-value)
0 15 30
p>0.05
e
cancer cell lines (n=922), mRNA
cancer cell lines (n=922), protein
s1 s2 s3 s4
694 subtype-specific genes
cancer type
differential expression
higher +1SD
lower −1SD
cancer type bladder/kidney, breast, brain/cns, cervix/ovary, colorectal, head/neck, leukemia, liver-biliary, lung, lymphoma, pancreas, prostate, sarcoma, secretory, skin, stomach/esophagus, thyroid, uterus, other
f
protein-based subtyping (mass spec.)
mRNA-based subtyping
s1 69 34 61 41 205
s2 19 130 29 74 252
s3 27 34 175 26 262
s4 17 48 34 104 203
n 132 246 299 245
relative enrichment -log10 (p-value)
0 15 30
p>0.05
no protein data

Figure S4. Observation of PDX-based molecular subtypes at the proteomic level. (a) The 287 TCGA tumors with mass spectrometry-based proteomic data from the Clinical Proteomic Tumor Analysis Consortium (CPTAC)[3] were classified according to PDX-based molecular subtype (Figure 1a). Expression patterns for a top set of 754 genes distinguishing between the four molecular subtypes based on the PDX transcriptomic compendium dataset (based on available proteomic data from the top 800 mRNAs from Figure 1a) are shown for both the TCGA mRNA dataset (left) and the TCGA proteomic dataset (right). Tumor profiles in both datasets are ordered by PDX-based subtype as assigned using mRNA data. Subtype-specific signature patterns high in each subtype by mRNA data are highlighted. **(b)** For the TCGA tumors with mass spectrometry-based proteomic data, the significance of overlap of the s1-s4 PDX-based subtypes as assigned based on mRNA data (rows) and on mass spectrometry-based proteomic data (columns). Enrichment p-values by one-sided Fisher's exact test. **(c)** The 7206 TCGA tumors with reverse-phase protein array (RPPA) data were classified according to PDX-based molecular subtype (from Figure 1a). Expression patterns for a top set of 129 genes distinguishing between the four molecular subtypes based on the PDX transcriptomic compendium dataset (see the section "Methods", based on available data) are shown for both the TCGA mRNA dataset (left) and the TCGA RPPA proteomic dataset (right). Tumor profiles in both datasets are ordered by PDX-based subtype as assigned using mRNA data. Subtype-specific signature patterns high in each subtype by mRNA data are highlighted. **(d)** For the TCGA tumors with RPPA data, the significance of overlap of the s1-s4 PDX-based subtypes as assigned based on mRNA data (rows) and on RPPA data (columns). Enrichment p-values by one-sided Fisher's exact test. The limitations of the proteomics-based analyses would include more limited proteomic data availability on tumors compared to mRNA and the fact that mRNA and protein levels do not highly correlate across tumors[3]. Despite these limitations, significant levels of concordance are observed here between the mRNA-based subtyping and the protein-based subtyping (parts b and d). **(e)** Observation of PDX-based molecular subtypes at the proteomic level in cancer cell lines. The 922 cell lines with both mRNA data and mass spectrometry-based proteomic data [9] were classified according to PDX-based molecular subtype (from Figure 1a). Expression patterns for a top set of 694 mRNA distinguishing between the four molecular subtypes based on the PDX transcriptomic compendium dataset (based on available proteomic data from the top 800 mRNAs from Figure 1a) are shown for both the mRNA dataset (left) and the proteomic dataset (right). Cell line profiles in both datasets are ordered by PDX-based subtype as assigned using mRNA data. Subtype-specific signature patterns high in each subtype by mRNA data are highlighted. **(f)** For the 922 cell lines, the significance of overlap of the s1-s4 PDX-based subtypes as assigned based on mRNA data (rows) and on mass spectrometry-based proteomic data (columns). Enrichment p-values by one-sided Fisher's exact test. Related to Figure 1.

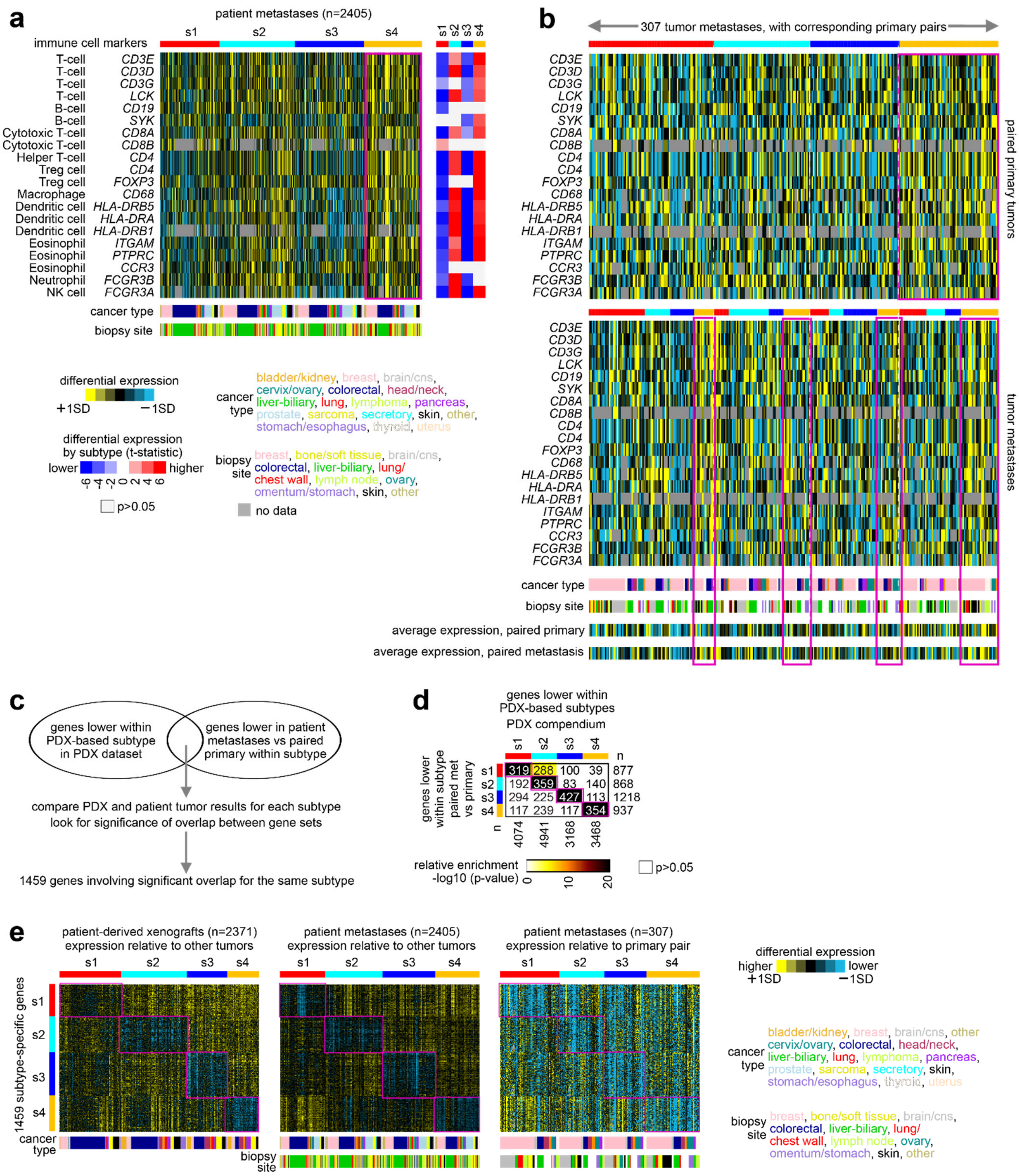
a
patient metastases (n=2405)
immune cell markers
s1
s2
s3
s4
T-cell CD3E
T-cell CD3D
T-cell CD3G
T-cell LCK
B-cell CD19
B-cell SYK
Cytotoxic T-cell CD8A
Cytotoxic T-cell CD8B
Helper T-cell CD4
Treg cell CD4
Treg cell FOXP3
Macrophage CD68
Dendritic cell HLA-DRB5
Dendritic cell HLA-DRA
Dendritic cell HLA-DRB1
Eosinophil ITGAM
Eosinophil PTPRC
Eosinophil CCR3
Neutrophil FCGR3B
NK cell FCGR3A
cancer type
biopsy site
differential expression
+1SD
−1SD
differential expression by subtype (t-statistic)
lower
higher
-6 -4 -2 0 2 4 6
p>0.05
cancer type
bladder/kidney, breast, brain/cns, cervix/ovary, colorectal, head/neck, liver-biliary, lung, lymphoma, pancreas, prostate, sarcoma, secretory, skin, other, stomach/esophagus, thyroid, uterus
biopsy site
breast, bone/soft tissue, brain/cns, colorectal, liver-biliary, lung/chest wall, lymph node, ovary, omentum/stomach, skin, other
no data
b
307 tumor metastases, with corresponding primary pairs
paired primary tumors
tumor metastases
cancer type
biopsy site
average expression, paired primary
average expression, paired metastasis
c
genes lower within PDX-based subtype in PDX dataset
genes lower in patient metastases vs paired primary within subtype
compare PDX and patient tumor results for each subtype
look for significance of overlap between gene sets
1459 genes involving significant overlap for the same subtype
d
genes lower within PDX-based subtypes
PDX compendium
genes lower within subtype paired met vs primary
s1 319 288 100 39 877
s2 192 359 83 140 868
s3 294 225 427 113 1218
s4 117 239 117 354 937
n 4074 4941 3168 3468
relative enrichment -log10 (p-value)
0 10 20
p>0.05
e
patient-derived xenografts (n=2371) expression relative to other tumors
patient metastases (n=2405) expression relative to other tumors
patient metastases (n=307) expression relative to primary pair
1459 subtype-specific genes
cancer type
biopsy site
differential expression
higher
lower
+1SD
−1SD

Figure S5. Additional information involving paired metastasis-primary comparisons (a) Expression changes in immune cell markers involving the s4 subtype. For the patient tumor metastases compendium dataset, the heat map shows differential patterns for key genes representing immune cell markers[4]. On the right are the corresponding gene-level t-statistics (by t-test), comparing tumors in the given subtype with the rest of the tumors. Most of these markers tend to be highest in the s4 subtype. **(b)** For the 307 patient tumor metastasis expression profiles for which expression profiles for the paired primary were available, both the primary and the metastasis were classified for the PDX-based subtypes (Figure 2c). The expression heat map represents the immune cell marker expression patterns of the tumor metastases in relation to the patterns for the corresponding paired primary tumors. In many instances, subtype switching events in patient metastases could be associated with expression changes in immune cell markers involving the s4 subtype. These patterns are highlighted, for example, in the heat maps in part b showing the average differential expression patterns in the paired primary versus paired metastasis samples. **(c)** Subtype-specific gene expression differences overlap highly with metastasis versus paired primary differences, focusing on the genes under-expressed by subtype. Schematic of gene set comparisons. For each PDX-based subtype, the set of genes low within that subtype versus the rest of the PDX tumors were overlapped with the set of genes low in patient metastases of the same PDX-based subtype versus the corresponding paired primaries. A set of 1459 genes involve significant gene set overlaps ($p<1E-10$, one-sided Fisher's exact test) between PDX comparisons and paired patient metastasis comparisons for the same subtype, involving all four subtypes. **(d)** Significance of overlap between the genes low within each of the PDX-based subtypes (using t-test $p<0.01$, based on analysis of PDX compendium) and the genes low within paired patient metastasis versus primary within each subtype ($p<0.01$, paired t-test, based on analysis of the patient tumor metastasis compendium). P-values by one-sided Fisher's exact test. From these results, a set of 1459 genes involve significant gene set overlap ($p<0.0001$) for the same subtypes (e.g., 319 overlapping s1-s1 genes, 359 s2-s2 genes, etc.). **(e)** Differential expression patterns for the top set of 1459 genes involving significant gene set overlaps for any of the four PDX-based subtypes are shown across the PDX compendium dataset (differential expression relative to other tumors), patient tumor metastases compendium dataset (relative to other tumor metastases), and patient tumor metastasis versus paired primary compendium dataset (relative to primary pair). Subtype-specific expression patterns are highlighted. Related to Figure 3.

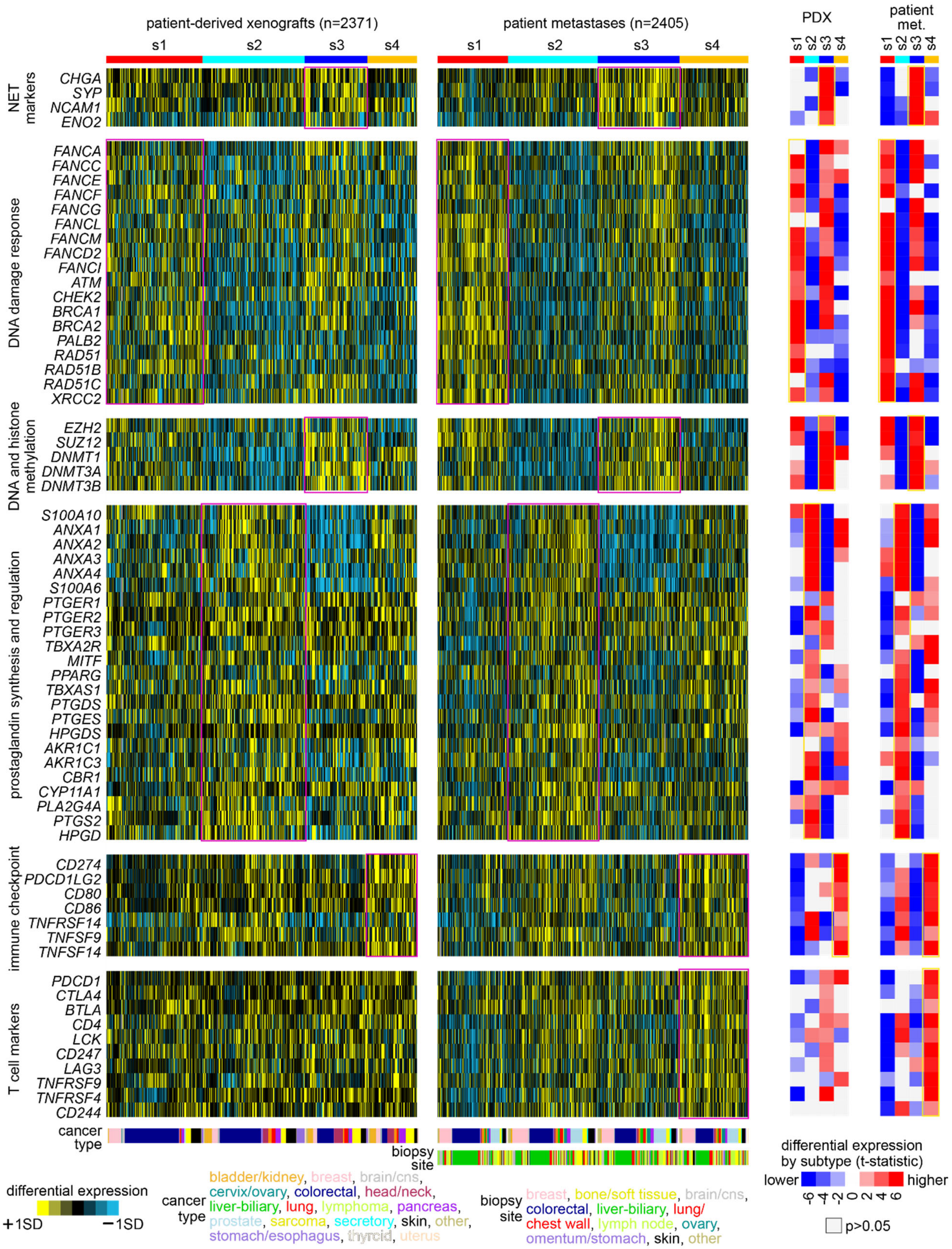

patient-derived xenografts (n=2371)
patient metastases (n=2405)
s1
s2
s3
s4
PDX
patient met.
NET markers
CHGA
SYP
NCAM1
ENO2
DNA damage response
FANCA
FANCC
FANCE
FANCF
FANCG
FANCL
FANCM
FANCD2
FANCI
ATM
CHEK2
BRCA1
BRCA2
PALB2
RAD51
RAD51B
RAD51C
XRCC2
DNA and histone methylation
EZH2
SUZ12
DNMT1
DNMT3A
DNMT3B
prostaglandin synthesis and regulation
S100A10
ANXA1
ANXA2
ANXA3
ANXA4
S100A6
PTGER1
PTGER2
PTGER3
TBXA2R
MITF
PPARG
TBXAS1
PTGDS
PTGES
HPGDS
AKR1C1
AKR1C3
CBR1
CYP11A1
PLA2G4A
PTGS2
HPGD
immune checkpoint
CD274
PDCD1LG2
CD80
CD86
TNFRSF14
TNFSF9
TNFSF14
T cell markers
PDCD1
CTLA4
BTLA
CD4
LCK
CD247
LAG3
TNFRSF9
TNFRSF4
CD244
cancer type
biopsy site
differential expression
+1SD
−1SD
cancer type
bladder/kidney, breast, brain/cns, cervix/ovary, colorectal, head/neck, liver-biliary, lung, lymphoma, pancreas, prostate, sarcoma, secretory, skin, other, stomach/esophagus, thyroid, uterus
biopsy site
breast, bone/soft tissue, brain/cns, colorectal, liver-biliary, lung/ chest wall, lymph node, ovary, omentum/stomach, skin, other
differential expression by subtype (t-statistic)
lower
higher
-6 -4 -2 0 2 4 6
p>0.05

Figure S6. Differential expression patterns involving key genes in specific pathways. For PDX and patient tumor metastases compendium datasets, heat maps show differential patterns for key genes of interest highlighted elsewhere (e.g., genes in pathways highlighted in Figure 6). On the right are the corresponding gene-level t-statistics (by t-test), comparing tumors in the given subtype with the rest of the tumors. Related to Figure 6.

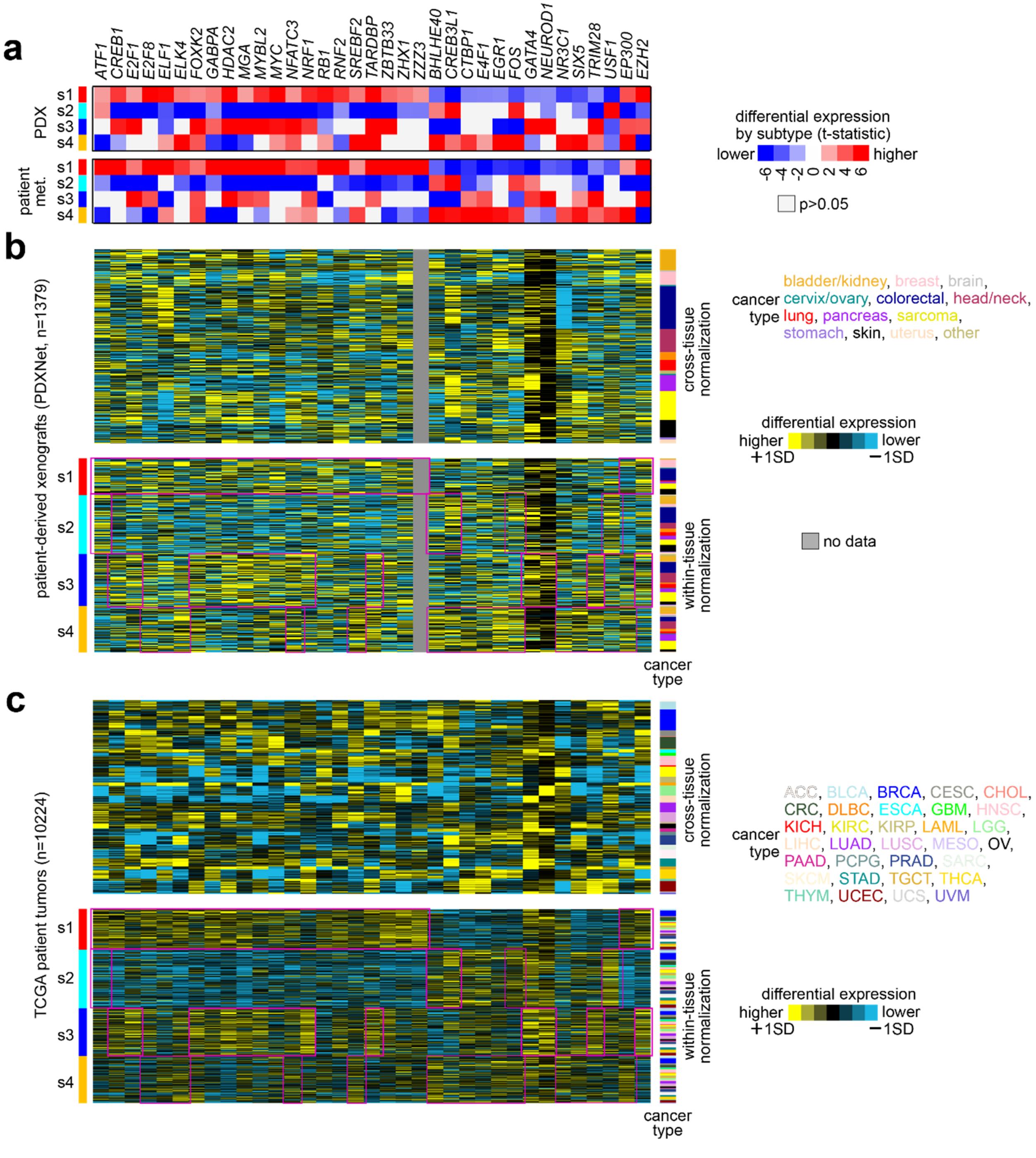

Figure S7. Lineage-specific gene expression patterns involving TFs with associations by subtype. (a) Top TF associations by subtype, taken from Figure 4b. The differential expression statistics by PDX-based subtype are represented for the top set of 35 TFs with both significant overlap between the TF-bound genes and genes over-expressed in the expression subtype and significantly higher or lower levels of the TF gene in that same subtype (both PDX and patient metastasis compendium datasets). **(b)** For the TF genes in part a, the corresponding differential expression patterns in the PDXNet dataset. The PDXNet dataset is normalized in two

ways: by cross-tissue normalization (top, values normalized to standard deviations from the median across all tumors) and within-tissue normalization (bottom, values normalized within each cancer type to standard deviations from the median). **(c)** Similar to part b, representing the TCGA pan-cancer dataset as normalized in the two different ways. We used the within-tissue normalization approach to compile our compendium expression datasets and the downstream analyses. By design, tissue-specific or lineage-specific expression differences are removed by within-tissue normalization, allowing us to identify pan-cancer subtypes that would transcend tissue- or cell-of-origin. The PDXNet and TCGA datasets feature cancers of various types profiled uniformly on a common platform, allowing us to examine expression patterns by cross-tissue normalization. With cross-tissue normalization, lineage-specific TF gene expression patterns can be observed. As intended, within-tissue normalization allows us to identify uniform differential patterns within a given pan-cancer subtype. Related to Figure 4.

References

1. Lek, M., Karczewski, K.J., Minikel, E.V., Samocha, K.E., Banks, E., Fennell, T., O'Donnell-Luria, A.H., Ware, J.S., Hill, A.J., Cummings, B.B., et al. (2016). Analysis of protein-coding genetic variation in 60,706 humans. Nature *536*, 285-291. 10.1038/nature19057.
2. Chen, F., Zhang, Y., Gibbons, D., Deneen, B., Kwiatkowski, D., Ittmann, M., and Creighton, C. (2018). Pan-cancer molecular classes transcending tumor lineage across 32 cancer types, multiple data platforms, and over 10,000 cases. Clin Cancer Res. *24*, 2182-2193.
3. Zhang, Y., Chen, F., Chandrashekar, D., Varambally, S., and Creighton, C. (2022). Proteogenomic characterization of 2002 human cancers reveals pan-cancer molecular subtypes and associated pathways. Nat Commun *13*, 2669.
4. Chen, F., Chandrashekar, D., Varambally, S., and Creighton, C. (2019). Pan-cancer molecular subtypes revealed by mass-spectrometry-based proteomic characterization of more than 500 human cancers. Nat Commun *10*, 5679.
5. Alzubi, M., Turner, T., Olex, A., Sohal, S., Tobin, N., Recio, S., Bergh, J., Hatschek, T., Parker, J., Sartorius, C., et al. (2019). Separation of breast cancer and organ microenvironment transcriptomes in metastases. Breast Cancer Res *21*, 36.
6. Sueyoshi, K., Komura, D., Katoh, H., Yamamoto, A., Onoyama, T., Chijiwa, T., Isagawa, T., Tanaka, M., Suemizu, H., Nakamura, M., et al. (2021). Multi-tumor analysis of cancer-stroma interactomes of patient-derived xenografts unveils the unique homeostatic process in renal cell carcinomas. iScience *24*, 10332.
7. Creighton, C.J., Bromberg-White, J.L., Misek, D.E., Monsma, D.J., Brichory, F., Kuick, R., Giordano, T.J., Gao, W., Omenn, G.S., Webb, C.P., and Hanash, S.M. (2005). Analysis of tumor-host interactions by gene expression profiling of lung adenocarcinoma xenografts identifies genes involved in tumor formation. Mol Cancer Res *3*, 119-129.
8. Creighton, C., Kuick, R., Misek, D., Rickman, D., Brichory, F., Rouillard, J.-M., Omenn, G., and Hanash, S. (2003). Profiling of pathway-specific changes in gene expression following growth of human cancer cell lines transplanted into mice. Genome biology *4*, R46.
9. Gonçalves, E., Poulos, R., Cai, Z., Barthorpe, S., Manda, S., Lucas, N., Beck, A., Bucio-Noble, D., Dausmann, M., Hall, C., et al. (2022). Pan-cancer proteomic map of 949 human cell lines. Cancer Cell *40*, 835-849.
