# Supplementary material for: Pan-cancer molecular subtypes of metastasis reveal distinct and evolving transcriptional programs
Source: Cell Rep Med. 2023 Feb 1;4(2):100932. doi: 10.1016/j.xcrm.2023.100932 (PMC9975284; doi:10.1016/j.xcrm.2023.100932)
Supplement: Document S2. Article plus supplemental information [file mmc9.pdf]

Cell Reports Medicine

Article

Pan-cancer molecular subtypes of metastasis reveal distinct and evolving transcriptional programs

Graphical abstract

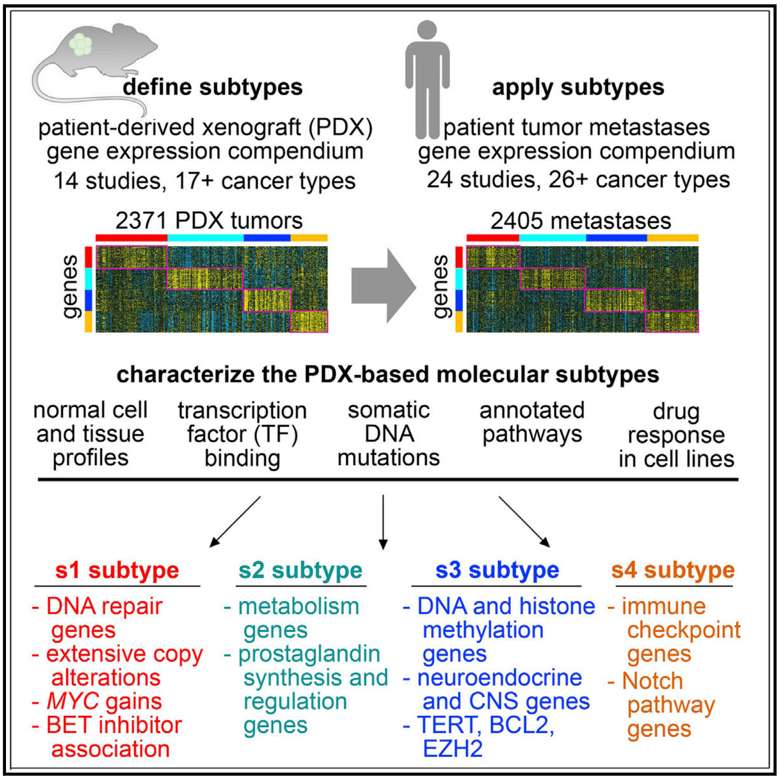

Highlights

- Four cancer cell-intrinsic, pan-cancer molecular subtypes are defined using PDX models
- Subtypes are present in patient metastases, primary tumors, and cancer cell lines
- Subtypes respectively involve MYC, prostaglandins, EZH2, and immune checkpoints
- Subtype switching is common between metastasis and paired primary by patient

Authors

Yiqun Zhang, Fengju Chen, Chad J. Creighton

Correspondence

creighto@bcm.edu

In brief

Analyzing gene expression data from patient-derived xenografts and patient metastases, collectively representing 38 studies and more than 3,000 patients and 4,000 tumors, Zhang et al. identify four pan-cancer expression-based subtypes of metastasis transcending tumor lineage. These subtypes have implications for applying existing therapies or developing new therapeutic approaches.

Zhang et al., 2023, Cell Reports Medicine *4*, 100932
February 21, 2023 © 2023 The Author(s).
https://doi.org/10.1016/j.xcrm.2023.100932

CellPress

Cell Reports Medicine

CellPress
OPEN ACCESS

Article

Pan-cancer molecular subtypes of metastasis reveal distinct and evolving transcriptional programs

Yiqun Zhang,[1,4] Fengju Chen,[1,4] and Chad J. Creighton[1,2,3,5,*]
[1]Dan L. Duncan Comprehensive Cancer Center Division of Biostatistics, Baylor College of Medicine, Houston, TX 77030, USA
[2]Human Genome Sequencing Center, Baylor College of Medicine, One Baylor Plaza, MS305, Houston, TX 77030, USA
[3]Department of Medicine, Baylor College of Medicine, Houston, TX 77030, USA
[4]These authors contributed equally
[5]Lead contact
*Correspondence: creighto@bcm.edu
https://doi.org/10.1016/j.xcrm.2023.100932

SUMMARY

Molecular mechanisms underlying cancer metastasis span diverse tissues of origin. Here, we synthesize and collate the transcriptomes of patient-derived xenografts and patient tumor metastases, and these data collectively represent 38 studies and over 3,000 patients and 4,000 tumors. We identify four expression-based subtypes of metastasis transcending tumor lineage. The first subtype has extensive copy alterations, higher expression of MYC transcriptional targets and DNA repair genes, and bromodomain inhibitor response association. The second subtype has higher expression of genes involving metabolism and prostaglandin synthesis and regulation. The third subtype has evidence of neuronal differentiation, higher expression of DNA and histone methylation genes and EZH2 transcriptional targets, and BCL2 inhibitor response association. The fourth subtype has higher expression of immune checkpoint and Notch pathway genes. The metastasis subtypes reflect expression differences from paired primaries, with subtype switching being common. These subtypes facilitate understanding of the molecular underpinnings of metastases beyond tissue-oriented domains, with therapeutic implications.

INTRODUCTION

Metastasis is the process by which cancer cells leave the primary tumor site and adapt to a distant tissue microenvironment.[1] Metastasis causes most cancer deaths.[1–3] Invasion and metastasis represent complex processes initiated later in the disease process, with associated molecular mechanisms broadly common across multiple cancer types defined by tissue of origin.[3,4] There is a clear need to understand better the processes and pathways underlying metastasis. Global molecular profiling is one approach that can lead to additional insights. For example, transcriptional profiling of metastases and primary tumors taken from the same patient could assess relative changes in gene expression that occur in the metastases. Previous studies have transcriptionally profiled patient tumor metastases, with the associated datasets deposited into the public domain. Most of these studies have focused on a specific cancer type, but other studies involved multiple cancer types and tissues of origin.[5,6] There is potential in combining molecular profiling data from individual metastasis studies, with available data to date involving thousands of patients and spanning multiple cancer types.

Cancer is a heterogeneous disease, and molecular subtyping of cancers can help identify pathways and processes underlying specific cancer subsets. With breast cancer being a well-known example, molecular subtypes can point to optimal therapeutic approaches for an individual tumor.[7] Based on transcriptome data from over 10,000 patient tumors in The Cancer Genome Atlas (TCGA), representing 32 different cancer types, we previously found that these tumors could be grouped into 10 major pan-cancer classes or subtypes.[8] By virtue of our analytical approaches, these TCGA-based pan-cancer subtypes spanned tissue of origin and tumor histology. The pan-cancer subtypes reflected the results of previous molecular profiling studies of individual cancer types, including subtypes related to cancer cell proliferation, immune cell infiltration, and cancer-associated stroma.[8] Except for TCGA melanoma cases, all but a small minority of TCGA tumors represent primary tumors and not metastases.[9] Our pan-cancer molecular subtyping approaches remained to be applied to patient tumor metastases.

Tumors resected from patients represent a mixture of cancer and non-cancer cells, as reflected in their molecular profiles. The tumor microenvironment would include immune cells, fibroblasts, and endothelial cells, all of which may be conscripted by the cancer cells to play a role in tumor biology.[3] In addition, human metastasis samples would include non-cancer tissues from the biopsy site, representing a major confounder in distinguishing true biology from technical artifact. Effective deconvolution of the contribution of cancer versus normal expression in the

© 2023 The Author(s).
This is an open access article under the CC BY license (http://creativecommons.org/licenses/by/4.0/).

tumor expression profile can be challenging.[10] One approach to address this issue is to profile tumors from patient-derived xenografts (PDXs), whereby a fragment of a patient's tumor is implanted into a mouse. In PDXs, the stromal components of the original tumor are substituted by their murine counterparts as a result of xenotransplantation.[11] A gene expression profile of a PDX tumor reflects human gene transcripts from the cancer cells, where the contribution of mouse transcripts from the tumor stroma would be minimal.[11–14] PDX models would also represent a type of metastasis, as cancer cells taken from their primary site are made to adapt to a foreign tissue microenvironment.

This study aimed to define pan-cancer molecular-based subtypes of metastasis that would transcend tumor lineage. To this end, we assembled two compendium expression datasets from the public domain, one of PDXs and one of patient tumor metastases, these data collectively representing over 3,000 patients and 38 studies. We removed cancer type- and laboratory-specific differences from each individual published dataset,[8,15] allowing for the identification of pan-cancer phenomena that would span data from multiple studies. We followed a previously demonstrated approach,[11] but applied here to metastasis and greatly expanded to incorporate multiple cancer types and studies. We used the PDX compendium expression dataset to define four expression-based molecular subtypes to minimize the contribution of non-cancer cells. We then applied these molecular subtypes to profiles of patient tumor metastases. We examined the subtype-associated differential expression patterns in the context of metastases versus paired primary differences within the same patient. We could also characterize the metastasis subtypes in terms of associated pathways, copy number alterations, and integration with results of external studies.

RESULTS

Compendium expression datasets of PDXs and patient tumor metastases

For our study, we assembled three separate compendium mRNA expression datasets representing metastases, with the data involved being publicly available from 38 individual studies (Tables S1). Our compendium dataset of PDXs represented 2,371 tumors, 973 patients, 14 studies, and over 17 cancer types by tissue of origin (including colorectal, n = 894 tumors; skin, n = 218; sarcoma, n = 214; breast, n = 213; head/neck, n = 165; bladder, n = 150; pancreatic, n = 135; gastric, n = 117; lung, n = 86; kidney, n = 58; uterine, n = 29; medulloblastoma, n = 20; prostate, n = 17; glioblastoma, n = 12; cervical, n = 10; ovarian, n = 9; other, n = 24). Our compendium dataset of patient tumor metastases resected from patients represented 2,405 tumors, 2,158 patients, 24 studies, and over 26 cancer types (including colorectal, n = 695 tumors; breast, n = 413; prostate, n = 349; skin, n = 146; sarcoma, n = 99; ovarian, n = 90; pancreatic, n = 79; lung, n = 75; kidney, n = 65; liver-biliary, n = 52; head/neck, n = 51; thyroid, n = 48; bladder, n = 28; secretory, n = 27; lymphoma, n = 25; esophagus, n = 24; CNS, n = 23; gastric, n = 33; other, n = 83). Of the 2,405 tumors in the patient tumor metastasis compendium dataset, 307 patient tumor metastases (representing 291 patients and 8 cancer types) had a corresponding primary tumor pair from the same patient also profiled, allowing for paired analyses for differences in metastases versus primaries. To the individual datasets involved in the compendiums, we applied previously demonstrated analytical approaches (see STAR Methods)[8,15–17] to effectively erase expression differences according to laboratory, analytical platform, or cancer type. These approaches allowed us to identify global patterns that would cut across multiple datasets and cancer types.

Our study approach was first to define expression subtypes and associated differential genes using our PDX compendium dataset and then to classify each patient tumor metastasis expression profile according to these PDX-based subtypes. We characterized the salient features of each subtype, as described below. For our study, PDX tumors would represent metastases, with cancer cells taken from their original site and made to grow at a different site. The advantage of defining molecular subtypes using PDX models is that the contribution of non-cancer cells to the PDX tumor profile is minimized. RNA from mouse cells either hybridized to a human expression array chip or sequenced and aligned to the human genome yields a much lower signal than RNA from the human cancer cells.[11–14] In contrast, the profiles in our patient tumor metastasis compendium dataset would represent mixtures of cancer and stroma cells.[10] For each molecular subtype, we could determine which of the associated subtype-specific genes, based on analysis of the PDX dataset, were also differentially expressed in metastasis versus primaries by paired analysis, using our compendium of 307 metastases with primary pairs. Within most tissue-based cancer types represented in the compendium dataset, widespread differences between metastasis and primary by paired analysis were identifiable (Figures S1A and S1B and Table S2). However, a likely confounder here would involve differences in non-cancer cells between the primary site and the metastasis biopsy site (Figure S1C).

Expression-based subtypes of tumor metastases

We set out to identify molecular subtypes in our patient tumor metastasis compendium dataset. As a starting point, we classified PDX and patient tumor metastases expression profiles according to a set of pan-cancer subtypes—labeled c1 through c10—previously defined using TCGA datasets of predominantly primary tumors[8] (Figure S2A). The TCGA-based subtypes were represented in both PDX and patient tumor metastases, but notably with relatively fewer PDX tumors and weaker patterns for the TCGA "c3" and "c7" subtypes involving immune cells and tumor stroma, respectively. We then used the PDX compendium expression dataset to define molecular subtypes, to minimize the contribution of non-cancer cells. In contrast, subtypes defined using the patient tumor metastasis compendium could be confounded, for example, by the tissue biopsy site, which usually differs from the primary site (Figures S2B and S2C). Using a randomly selected set of 2,000 genes, the 2,371 PDX tumors in our compendium dataset separated into four distinct expression-based pan-cancer subtypes based on unsupervised clustering, labeled s1 through s4 (Figures S2D–S2F). In PDX tumors, the differential expression patterns associated with the subtypes were associated with human cancer cells over mouse stroma cells, as evidenced, for example, by analysis of PDX

CellPress
OPEN ACCESS

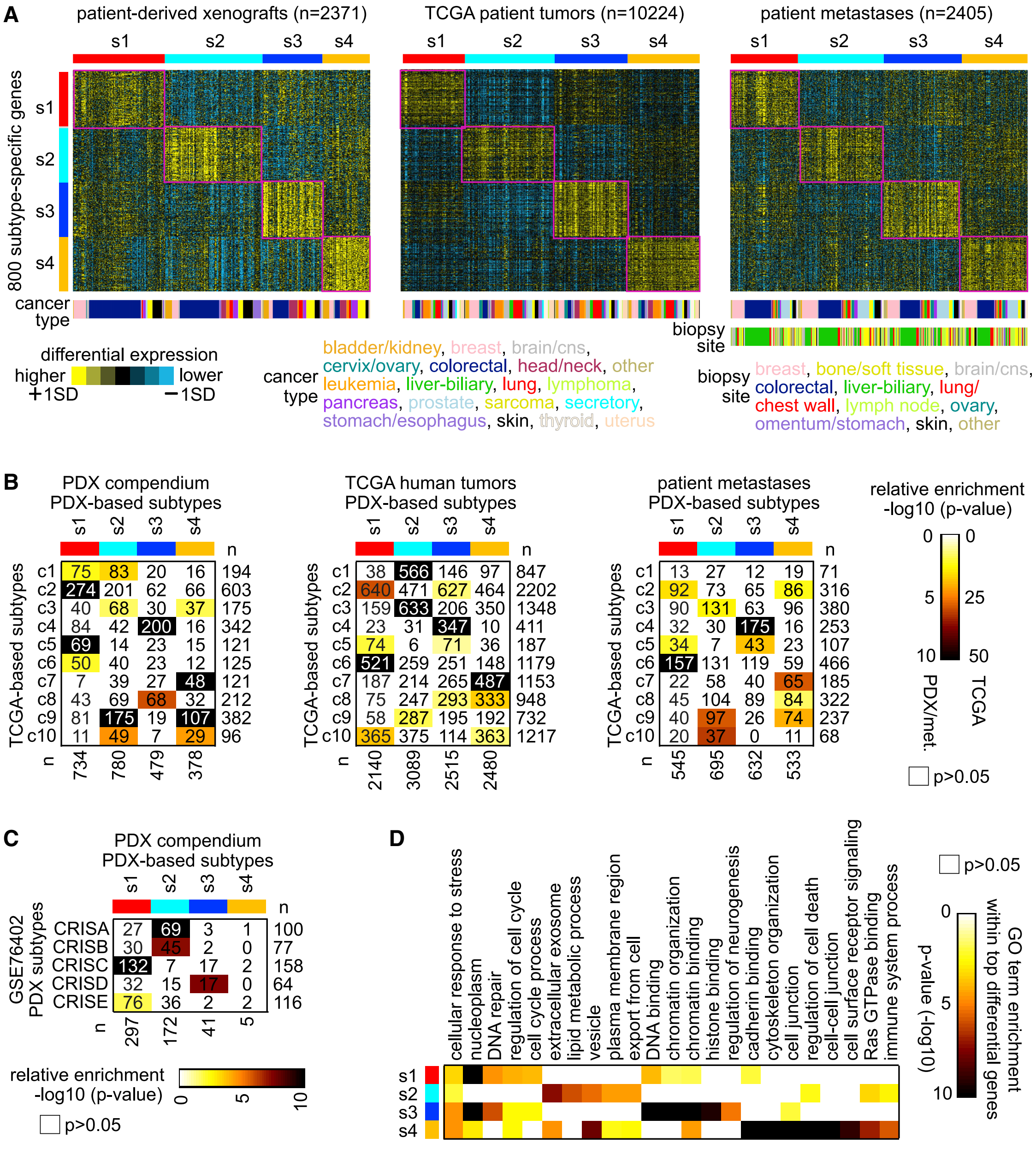

Figure 1. Pan-cancer molecular subtyping of tumor metastases

(A) The PDX compendium expression dataset defined four pan-cancer subtypes, s1 through s4 (see STAR Methods and Figure S2). Transcriptomic patterns for the top set of 800 mRNAs distinguishing between the four PDX-based subtypes are shown for PDX, TCGA, and patient tumor metastases datasets, with subtype-specific expression patterns highlighted. SD, standard deviation from the median within a given dataset and within cancer type.

(B) For PDX compendium, TCGA, and patient tumor metastases compendium datasets, the significance of overlap between the PDX-based subtype assignments and TCGA-based subtype assignments is indicated.

(C) For GSE76402 colorectal (CRC) PDXs in the PDX compendium 11, significance of overlap between our PDX-based subtype assignments and the CRC intrinsic subtypes (CRISs) based on the GSE76402 study.

(D) For the top overexpressed genes associated with each PDX-based subtype (from A), represented GO categories were assessed, with selected enriched categories represented here. For (B), (C), and (D), p values are by one-sided Fisher's exact test.

RNA-sequencing (RNA-seq) data aligned to both human and mouse genomes (Figure S3).

We classified profiles in the patient tumor metastasis compendium and TCGA datasets by PDX-based molecular subtype, using a gene classifier consisting of the top set of 800 differential mRNAs (Figure 1A, genes defined by comparing each subtype to the rest of the tumors). The PDX-based subtypes were well represented in both these datasets at similar proportions. For

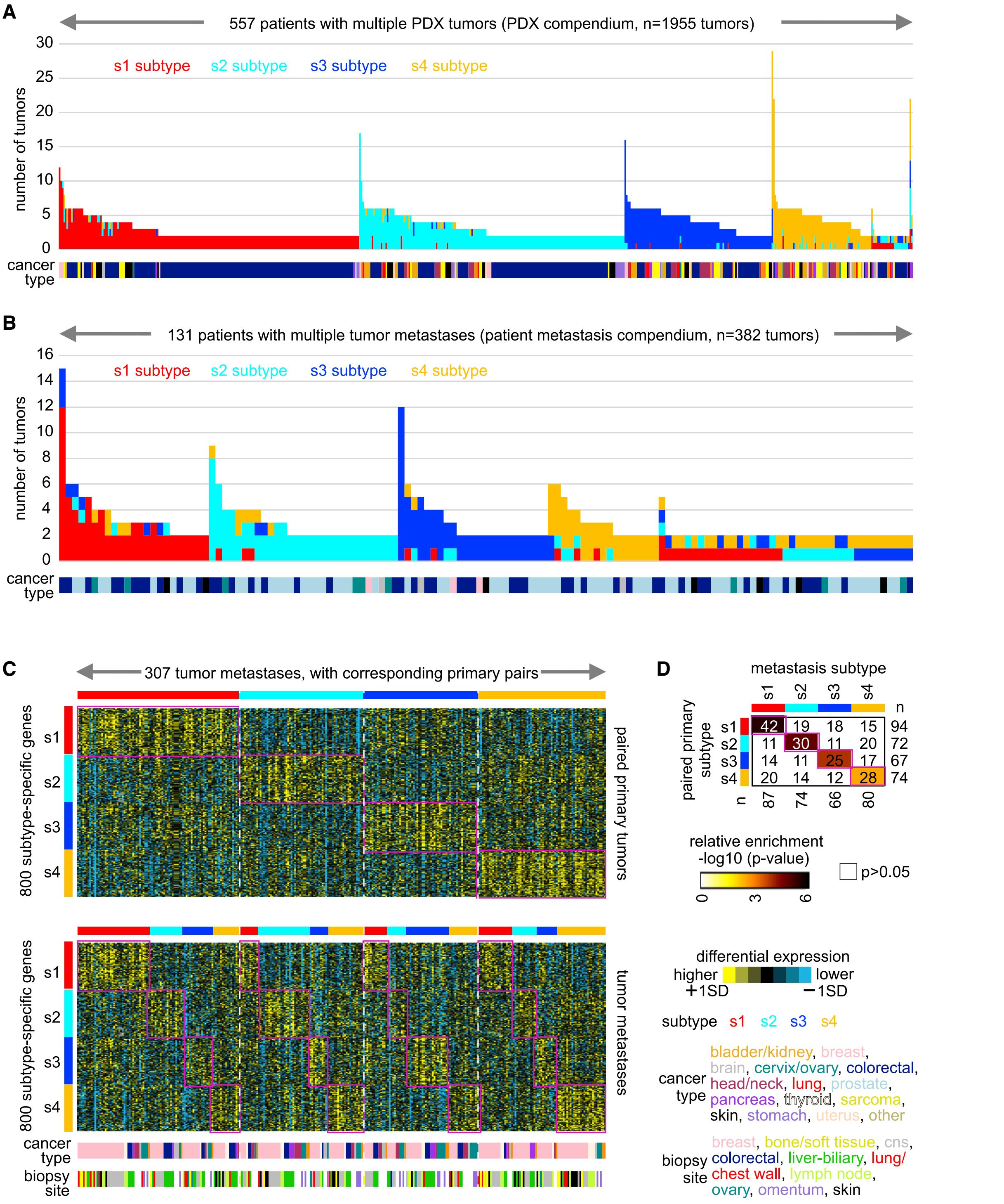

Figure 2. Molecular subtype assignments involving multiple tumors from the same patient

(A) By patient, molecular subtype assignments for 1,955 PDX tumors involving 557 patients with multiple PDX tumors represented in the PDX compendium expression dataset. Patients are sorted according to those for whom a plurality of tumors were of the same subtype (left) and those for whom no single subtype was represented in a plurality of tumors (right).

(B) By patient, molecular subtype assignments for 382 patient tumor metastases involving 131 patients with multiple tumor metastases represented in the patient metastasis compendium dataset. Patients are sorted according to those for whom a plurality of tumors were of the same subtype (left) and those for whom no single subtype was represented in a plurality of tumors (right).

(legend continued on next page)

TCGA tumors, of which a substantial number have proteomic data by mass spectrometry or by reverse-phase protein array, the PDX-based subtypes, as defined above at the mRNA level, were also reflected at the protein level (Figures S4A–S4D). Among the three datasets (PDX, patient metastasis, TCGA), there were significant patterns of overlap between the s1–s4 PDX-based subtype assignments and the previous TCGA c1–c10 subtype assignments (Figure 1B). Specifically, the current and previous subtyping correspondence included s1 to c2/c5/c6, s2 to c3/c9, s3 to c4, and s4 to c7. The c4 subtype was previously associated with neuroendocrine-like tumors. In addition, GSE76402 colorectal (CRC) PDX sample profiles in our compendium showed significant overlaps between our PDX-based subtype assignments and previously identified CRC intrinsic subtypes (CRISs),[11] with three of our four subtypes overlapping with four of the five CRIS subtypes (Figure 1C). Within the top differentially expressed genes underscoring each PDX-based subtype, specific gene categories (by Gene Ontology [GO] annotation) were overrepresented (Figure 1D and Table S3). Subtype s1 involved "DNA repair" and "cell-cycle process" genes; subtype s2 involved "extracellular exosome" genes; subtype s3 involved "DNA repair," "chromatin organization," and "histone binding" genes; and subtype s4 involved "cell junction," "Ras GTPase binding," and "immune system process" genes. Overall, the PDX-based subtypes did not strongly associate with patient metastasis biopsy site or tissue of origin. However, the s1 and s4 subtypes were enriched and antienriched, respectively, for CRC cases (Figures S2E and S2F).

Metastasis subtypes reflect expression differences from paired primaries

Multiple tumors from the same patient tended to share the same molecular subtype assignments. However, the subtype assignments would differ in a substantial fraction of cases, representing molecular subtype switching.[18] Of the 2,371 tumors in our PDX compendium expression dataset, 1,955—representing 557 patients—involved multiple tumors (two or more) originating from the same patient. Of the 557 patients, 530 (95%) had a plurality of tumors with the same subtype (Figure 2A). In our patient metastasis compendium expression dataset, 381 tumors—representing 131 patients—involved multiple tumors from the same patient, with 92 patients (70%) having a plurality of tumors with the same subtype (Figure 2B). For the 307 patient tumor metastases in our compendium for which paired primary data were available, we classified both primary and metastasis by PDX-based subtype. In most cases, the assigned subtype and associated expression patterns differed between the metastasis and the paired primary (Figure 2C). However, for 125 of the 307 tumor metastases (41%), the metastasis-based and primary-based subtype assignments were the same, these overlapping assignments being statistically significant (Figure 2D). In many instances, subtype switching events in patient metastases could be associated with expression changes in immune cell markers involving the s4 subtype (Figures S5A and S5B).

We wanted to explore expression differences between our molecular subtypes of metastasis in the context of metastasis versus paired primary comparisons involving metastasis-related changes within the patient. Our PDX compendium dataset defined differential expression patterns among the subtypes, while our paired metastasis and primary dataset could evaluate expression changes in metastasis using the primary as a baseline. Different gene sets from the respective datasets would represent orthogonal results, with significant correspondence between gene sets for the same molecular subtype of particular interest (Figure 3A). We identified highly significant gene set overlaps between PDX and paired patient metastasis comparisons for the same subtype, involving all four subtypes and 1,133 genes (Figure 3B and Tables S2 and S3). Differential subtype-specific expression patterns for these 1,133 genes appeared consistent across our PDX compendium dataset and patient tumor metastases compendium dataset, where we assessed differential expression for the latter relative to other metastases and relative to available primary pairs (Figure 3C). We also observed similar correspondence patterns between PDX and paired metastasis comparisons for genes with lower expression by subtype, involving 1,459 genes (Figures S5C–S5E). Subtype-specific genes involving the above included DNA repair-related genes for s1 subtype (*BRCA2*, *FANCD2*, *FANCF*), metabolism-related genes for s2 subtype (*ALDOB*, *COX5B*, *COX6A1*, *COX7A2*, *IDH1*, *SUCLG1*, *LDHD*), neuroendocrine marker genes for s2 subtype (*CDH2*, *NCAM1*), and Notch pathway genes for s4 subtype (*NOTCH1*, *NOTCH2*, *NOTCH3*; Figure 3C). Given that some 60% of metastases may have a different subtype from their corresponding primary tumor (Figure 3D), many observed differences between paired metastases and primaries may also involve subtype switching.

Central nervous system (CNS) and transcription factor (TF) associations by subtype

Our s3 metastasis subtype was strongly associated with the previously identified "c4" pan-cancer molecular subtype, which expressed markers of neuroendocrine tumors.[8] Neuronal differentiation occurring in epithelial cancer cells has been observed elsewhere.[19] To explore this association further, we examined the expression dataset from the Fantom consortium of 889 profiles representing various normal human cell and tissue specimens,[20] including 59 CNS-related cell types and tissues. Gene signatures of several CNS profiles—including those from neurons, astrocytes, whole brain, and spinal cord—were strongly manifested in s3, relative to the other subtypes, across PDX and patient tumor metastasis compendium datasets (Figure 4A). Furthermore, genes encoding canonical markers of

(C) For the 307 patient tumor metastasis expression profiles for which expression profiles for the paired primary were available, both the primary and the metastasis were classified for the PDX-based subtypes. The expression heatmap represents the subtype-associated expression patterns of the tumor metastases in relation to the patterns for the corresponding paired primary tumors.

(D) For the 307 tumor metastases represented in (C), the overlaps between the metastasis-based and the paired primary-based subtype assignments are shown. The p values were found by one-sided Fisher's exact test. For 125 of the 307 tumor metastases (41%), the metastasis-based and primary-based subtype assignments were the same.

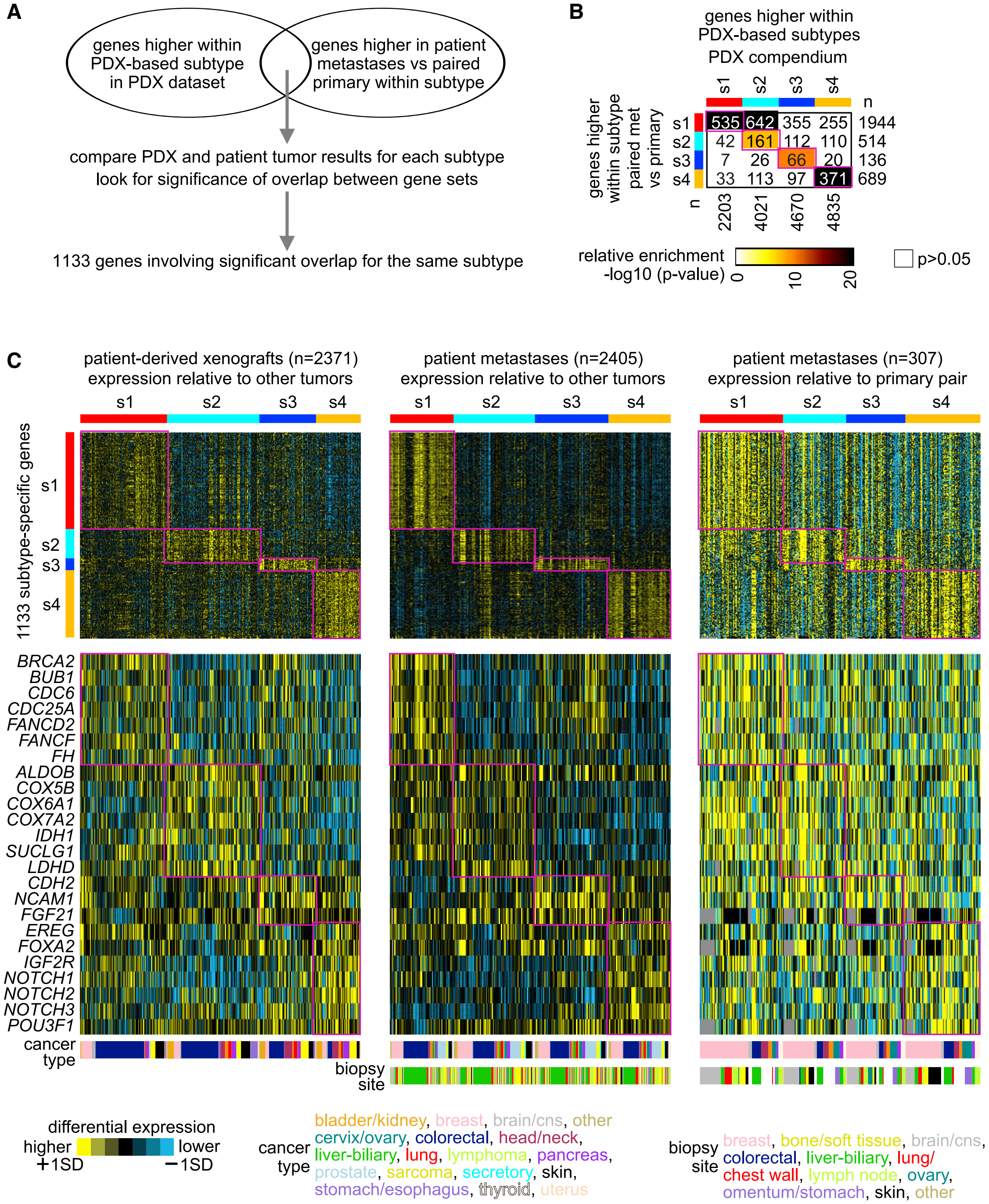

	s1	s2	s3	s4	n
s1	535	642	355	255	1944
s2	42	161	112	110	514
s3	7	26	66	20	136
s4	33	113	97	371	689
n	2203	4021	4670	4835	

Figure 3. Subtype-specific gene expression differences overlap highly with patient metastasis versus paired primary differences

(A) Schematic of gene set comparisons. For each PDX-based subtype, the set of genes high within that subtype versus the rest of the PDX tumors was overlapped with the set of genes high in patient metastases of the same PDX-based subtype versus the corresponding paired primaries. A top set of 1,133 genes involves significant gene set overlaps between PDX comparisons and paired patient metastasis comparisons for the same subtype, involving each of the four subtypes.

(B) Significance of overlap between the genes high within each of the PDX-based subtypes (using t test, $p < 0.01$, based on analysis of PDX compendium) and the genes high within paired patient metastasis versus primary within each subtype ($p < 0.01$, paired t test, based on analysis of the patient tumor metastasis compendium). Overlap p values were found by one-sided Fisher's exact test or chi-square test. From these results, a set of 1,133 genes involves significant gene set overlap ($p < 1E-6$) for the same subtypes (e.g., 535 overlapping s1–s1 genes, 161 s2–s2 genes, etc.).

(legend continued on next page)

neuroendocrine tumors—including *CHGA* (chromogranin A), *SYP* (synaptophysin), *NCAM1* (CD56), and *ENO2* (neuron-specific enolase)—were all differentially higher in s3 tumors (Figures 4A and S6 and Tables S3 and S4).

We surveyed TF binding upstream of each gene for the genes associated with higher subtype-specific expression (Table S5). Of 158 TFs with available data,[21] 87 were significantly enriched ($p < 1E-6$, one-sided Fisher's exact or chi-square test) with one or more of the subtype-specific gene sets, for both PDX and patient tumor metastases compendium datasets. Of the 87 TFs, 35 involved differential expression of the TF gene in the same subtype in both compendium datasets (Figure 4B). Most of these significant TF associations involved the s1 subtype, although notably, the s3 subtype showed both higher expression of the EZH2 gene and a strong enrichment for EZH2 transcriptional targets within genes higher in s3 versus other tumors. When integrating the above results with a limited set of gene expression signatures of TF knockdown by siRNA,[22] three TFs—E2F1, HDAC2, and MYC— had siRNA knockdown signature scoring significantly negative for the s1 subtype, along with the corresponding higher TF gene expression and TF gene target enrichment patterns (Figure 4C). The above MYC association is consistent with previous observations in primary tumors for the s1 subtype analogs.[8,16,17] As the compendium expression datasets involved our first removing cancer type-specific differences to arrive at our pan-cancer subtypes, this removed lineage-specific TF expression patterns, although such patterns could be observed in the original datasets before normalization (Figure S7). In addition to *trans*-acting TFs, *cis*-regulatory alterations, e.g., involving enhancer hijacking, would also be at work within metastatic tumors.[23]

Somatic mutation and copy gain events underlie metastasis subtypes

We explored subtype-specific expression differences involving gene copy number alteration (CNA) patterns. Of the 2,405 tumors in our patient metastasis compendium expression dataset, 934 had corresponding gene copy and somatic mutation information, as did 1,238 of the 2,371 tumors in the PDX expression compendium dataset. Consistent with previous observations involving s1 subtype analogs,[16] s1 tumors showed higher levels of CNA burden relative to s2 and s4 subtypes, as observed across the PDX compendium, the patient tumor metastasis compendium, and TCGA pan-cancer datasets (Figure 5A). In PDX compendium and TCGA datasets, s3 tumors also showed higher overall CNA burden. We also examined small somatic mutation events (single-nucleotide variants and insertions/deletions) for 102 cancer-associated genes in core oncogenic and tumor-suppressive pathways (Table S6).[8,16] For all three datasets surveyed (PDX compendium, patient tumor metastasis compendium, TCGA), one gene, *APC*, was consistently enriched for mutation events ($p < 0.01$, one-sided Fisher's exact test) in the s1 subtype, due in part to the relative enrichment of s1 for colorectal cancers (Figure S2F). *TP53* was also enriched for mutation events in the s1 subtype ($p < 0.01$) but for only the patient tumor metastasis and TCGA datasets. For the s2 subtype, six genes—*MTOR*, *PIK3CA*, *PTEN*, *BRAF*, *HRAS*, *KRAS*—were enriched for mutation events ($p < 0.01$) for exactly two of the three datasets surveyed.

For each gene represented in our compendium datasets, we assessed the significance of the enrichment of copy gain events within each molecular subtype. For the s1 and s3 subtypes, we identified significant gene set overlaps between genes with enriched copy gain events within a given subtype and genes highly expressed within the same subtype (based on the PDX compendium dataset). This pattern was consistent for all three datasets surveyed (PDX compendium, patient tumor metastasis compendium, TCGA; Figure 5B and Table S6). A set of 1,670 genes involved significant gene overlap between expression differences and copy gain enrichment patterns for either s1 or s3 subtypes for at least two of the three copy number datasets examined (Figure 5C). These 1,670 genes involved well-established cancer-related genes,[24] including *MYC*, *MYB*, *BRCA2*, *ERCC4*, and *MET* for s1 subtype and *TERT*, *BCL2*, and *SUZ12* for s3 subtype. As observed elsewhere,[7] copy alteration patterns may involve single-copy gains for known oncogenes and single-copy losses for tumor suppressor genes. Here, the above enrichment patterns mostly involve gene copy gain as opposed to gene amplification, and none of the genes in the patterns of gene set overlap exhibited high-level amplification akin to the HER2 gene in breast cancer.

Pathways represented by metastasis subtypes

Some of the above findings suggested the involvement of key pathways of interest underlying each metastasis subtype (Figures 6 and S6). The enrichment for DNA repair genes within genes higher in s1 tumors (Figure 1D and Table S3) and the higher CNA burden in s1 tumors (Figure 5A) suggested associations involving the DNA double-strand-break repair pathway and Fanconi anemia. These associations were evident when examining key individual genes, including *BRCA1*, *BRCA2*, *FANCD2*, *FANCI*, and *RAD51* (Figure 6A). Many of the genes in this pathway were also higher in s3 tumors, which subtype similarly involved enrichment patterns for DNA repair genes (Figures 1D and S6). The observed association of EZH2 transcription targets with s3 tumors (Figure 4B) and of *SUZ12* copy gain events in s3 tumors (Figure 5C) suggested processes of histone methylation and DNA methylation, with genes higher in s3 also including *DNMT1*, *DNMT3B*, and *DNMT3A* (Figure 6B).

Regarding s2 tumors, when taking the set of genes higher ($p < 0.01$, t test) in s2 versus other tumors for both PDX and patient metastases compendium expression datasets, enriched wikiPathways[26] ($p < 0.00005$, one-sided Fisher's exact test) included the prostaglandin synthesis and regulation pathway (Figure 6C). This association included annexin genes, *S100A10*, *S100A6*, *PTGER1*, *PTGER2*, *PTGS2*, *TBXA2R*, *AKR1C3*, and *HPGD*. Prostaglandin

(C) Differential expression patterns for the top set of 1,133 genes involving significant gene set overlaps for any of the four PDX-based subtypes are shown across the PDX compendium dataset (differential expression relative to other tumors), patient tumor metastases compendium dataset (relative to other tumor metastases), and patient tumor metastasis versus paired primary compendium dataset (relative to primary pair). Subtype-specific expression patterns are highlighted. Selected genes of interest from the 1,133 genes are also represented individually by differential patterns.

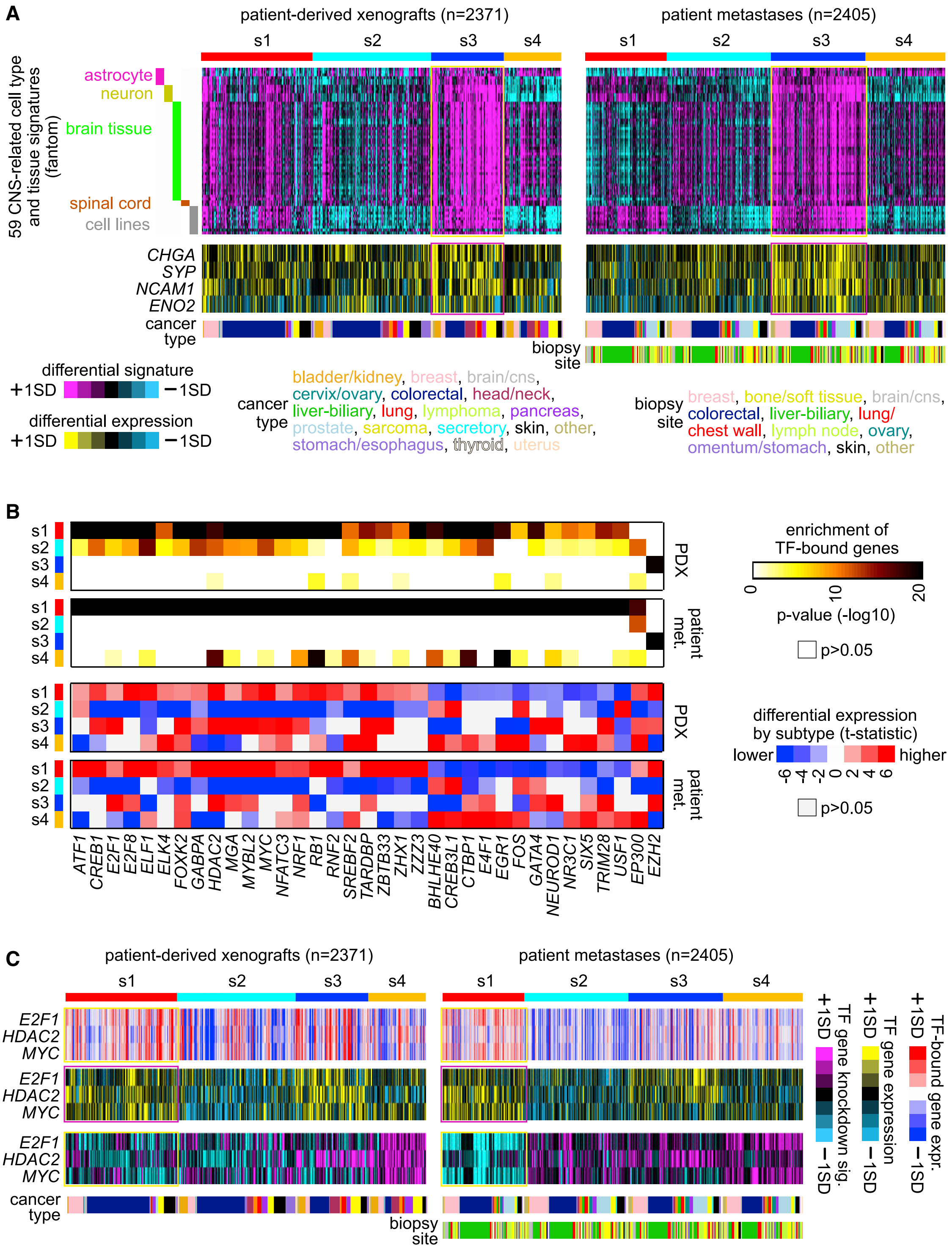

Figure 4. Central nervous system (CNS) and transcription factor (TF) associations by subtype

(A) CNS associations by subtype. Heatmaps showing intersample correlations (purple, positive; cyan, negative) between mRNA profiles of tumors in PDX and patient metastasis compendium datasets (columns) and mRNA profiles of Fantom[20] cell types or tissues related to the CNS (rows).

(B) Top TF associations by subtype. Encode[21] data on TF binding was extracted for 158 TFs, with gene associations defined by binding within 2 kb upstream of the gene start. For the set of 35 TFs represented, there was both significant overlap ($p < 1E-6$, one-sided Fisher's exact test or chi-square test) between the TF-bound genes and the genes overexpressed in the expression subtype and significantly higher or lower levels of the TF gene in that same subtype ($p < 0.05$ by t test), for both PDX and patient metastasis compendium datasets.

(legend continued on next page)

E2 (PGE2) can promote tumor growth by binding to its receptors and activating signaling pathways that control cell proliferation, migration, apoptosis, or angiogenesis.[27] Regarding s4 tumors, immune system-related genes were enriched in genes higher in s4 versus other tumors (Figure 1D). Along these lines, the s4 subtype had higher expression of several genes in the immune checkpoint pathway, representing potential targets for immunotherapy,[15,28] including *PDCD1* (PD1), *CD274* (PDL1), and *PDCD1LG2* (PDL2) (Figure 6D). Consistent with our understanding of the PDX model, genes in the immune checkpoint pathway with specific roles in the T cells interacting with the antigen-presenting cells were significantly higher in s4 tumors from patient metastases but not from PDXs (Figures 6D and S6). Several genes—including *CD247* (CD3), *CTLA4* (CD152), *TNFRSF4* (CD134), *LAG3*, and T cell marker *LCK*—were significantly higher ($p < 0.001$, t test) in s4 tumors from patient metastases but not significantly ($p > 0.05$) in s4 PDX tumors. These differential expression patterns would be consistent with the scenario of tumor cells with antigen presentation interacting with T cells in human tumors, where PDX tumors would not represent the T cell component.

Associations of metastasis subtypes with drug responses in cancer cell lines

Integrating molecular data on cancer cell lines with their responses to anticancer drugs can identify therapeutic options for cancer subsets.[29] Similar to the above external expression datasets, we assigned transcriptomic profiles for each of 958 cell lines in the Genomics of Drug Sensitivity in Cancer (GDSC) dataset to a metastasis subtype (Figure 7A). PDX-based subtypes were reflected in cancer cell lines at both the mRNA and the protein levels (Figures S4E and S4F). For each of 544 drug compound treatments with half-maximal inhibitory concentration (IC_{50}) measurements, we compared IC_{50} values for cell lines of a given subtype with the rest of the cell lines. Widespread associations of molecular subtype with drug response, well exceeding chance expected, were found for s1, s3, and s4 subtypes (Figure 7B and Table S7). At $p < 0.001$ significance level (t test on natural log values), 45, 199, and 9 drug treatments showed greater sensitivity levels in s1, s3, and s4 cell lines, respectively. These drug response associations aligned with the above molecular observations involving the subtypes. For example, s1 subtype associated with response to several bromodomain inhibitors (Figures 7B and 7C), conceivably related to this subtype's association with MYC.[30] Several drugs targeting chromatin histone acetylation or methylation associated with response in s3 cell lines (Figure 7B). The one drug in GDSC targeting EZH2 showed sensitivity in s3 as well as s1 cell lines (Figure 7C). With s3 involving both *BCL2* overexpression and copy gain (Figure 5C), s3 also associated with greater sensitivity ($p < 0.01$) to all six BCL2 inhibitors represented in GDSC (Figure 7C and Table S7). With s3 also involving both *TERT* overexpression and copy gain (Figure 5C), s3 associated here with greater sensitivity to TERT inhibition (Figure 5C). Previous studies involving tumor xenografts demonstrate how GDSC IC_{50} values would translate into substantial anticancer effects *in vivo*. For example, BRD4 inhibitors impact tumor growth of s1 cell lines MDA-MB-231 and MDA-MB-468,[31] BCL2 inhibitors impact tumor growth of s3 cell line OVCAR8,[32] and telomerase inhibitor impacts tumor growth of s3 cell line HeLa.[33]

DISCUSSION

By transcriptomics, our study uncovered four major pan-cancer molecular subtypes of metastases. The s1 subtype had extensive copy alterations, higher expression of genes involved in DNA double-strand-break repair, higher expression of TF genes such as *MYC* with corresponding higher expression of their transcriptional target genes, and associations with bromodomain inhibitor response. The s2 subtype had higher expression of genes involved in metabolism and prostaglandin synthesis and regulation. The s3 subtype had higher expression of DNA and histone methylation genes, higher expression of EZH2 and associated transcriptional targets, higher expression of neuroendocrine marker genes and evidence of a type of neuronal differentiation, and higher expression and copy gain of *BCL2* coupled with BCL2 inhibitor response associations. The s4 subtype had higher expression of immune checkpoint and Notch pathway genes. These subtypes were manifested in primary and metastatic tumors, consistent with the notion that metastasis-associated transcriptional programs may be encoded within primary tumors.[34] By our analytical approach, the molecular subtypes spanned tumors of diverse lineages and tissues of origin and multiple datasets from independent laboratories. The idea that cancer metastases can be categorized into a handful of distinct groups would have important implications for understanding the biology of metastasis. For example, different processes and pathways that appear coordinately manifested in a cancer subtype might suggest a degree of cooperation between these that could be explored further.

The metastasis subtypes reflected expression differences from paired primaries, with subtype switching being common. Metastatic cells that escape from the primary tumor may develop into tumors of a different molecular subtype from that of the primary, while still falling within one of a discrete set of subtypes. When considering the subset of patient tumor metastasis according to a particular subtype, we could observe widespread paired differences between metastases and paired primary, these differences spanning multiple cancer types. Many previous studies (e.g., those from which we incorporated data into the present study) have sought to define global expression differences between metastases and paired primaries for a given cancer type. These previous studies may not have considered the site of metastasis biopsy

(C) Of the 35 TFs represented in (B), 10 were represented in an expression profiling dataset of siRNA knockdown of specific genes.[22] For three TFs—E2F1, HDAC2, and MYC—the genes were highly expressed in the associated s1 subtype, with the corresponding siRNA knockdown signature scoring significantly negative ($p < 1E-6$, t test) for both PDX and patient tumor metastases datasets. For these three TFs, the associated patterns are represented across PDX and patient tumor metastasis compendium datasets: average differential expression of the TF-bound genes (red-blue heat maps), differential expression of the TF genes (yellow-blue heat maps), and differential levels of the TF gene siRNA signature (purple-cyan heat maps). A negative siRNA signature association indicates that knocking down the TF gene would result in a global pattern opposite TF gene overexpression.

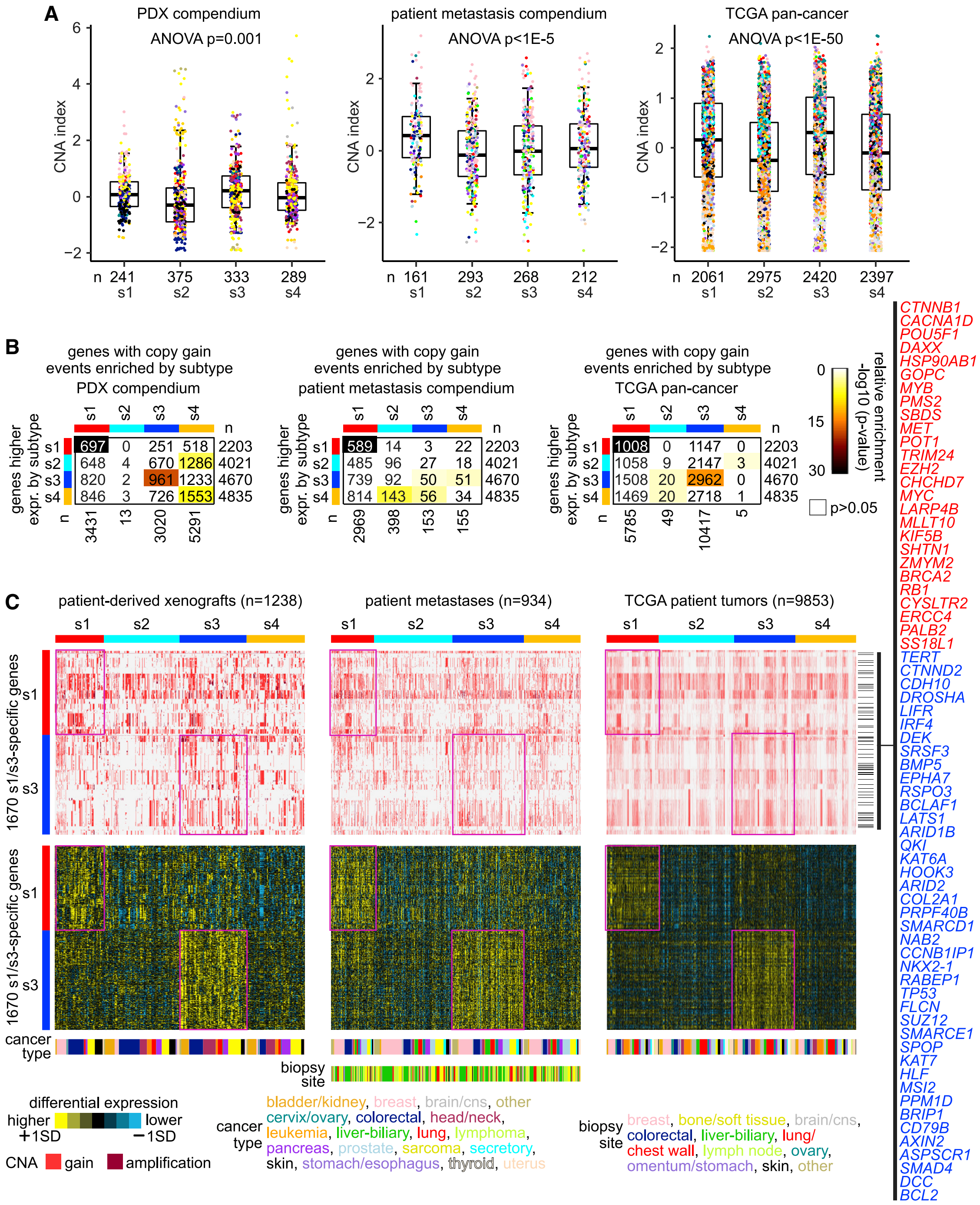

Figure 5. Copy number alteration (CNA) events underlying the molecular subtypes of metastasis

(A) For PDX compendium, patient metastasis compendium, and TCGA datasets, overall CNA burden index (standard deviation of CNA values across all genes, centered within each dataset to standard deviations from the median across samples) by PDX-based molecular subtype. Boxplots represent 5% (lower whisker), 25% (lower box), 50% (median), 75% (upper box), and 95% (upper whisker).

(B) For PDX compendium, patient metastasis compendium, and TCGA datasets, significance of overlap between the genes high within each of the PDX-based subtypes (using t test, $p < 0.01$, based on analysis of PDX compendium) and the genes with copy gain events more frequent within each subtype ($p < 0.01$, one-sided Fisher's exact test, PDX and patient metastasis datasets; $p < 0.001$, chi-square test, TCGA dataset). Overlap p values were found by chi-square test. A set of 1,670 genes involves significant gene set overlaps between expression differences and copy gain enrichment patterns for the same subtype, for at least two of the three copy number datasets examined, these significant overlaps involving s1 and s3 subtypes.

(legend continued on next page)

as a confounding variable, with expression differences largely reflecting differences between tissues of the primary site versus other tissues. Our PDX-based approaches to molecular subtyping circumvented this issue. In addition to global expression differences observed across all metastases versus primary tumors, consistent differences involving only a subset of patients may be considered, as done in our study. For molecular subtypes associated with higher overall levels of CNA, gene copy gain events that increase expression of genes underlying the subtype may be evolutionarily favored. These events might involve a gain of just one or two copies versus the high-level amplification events favoring strong cancer driver genes.

Our molecular subtypes could have important implications for applying existing therapies or developing alternate therapeutic approaches. Therapies potentially targeting subtypes would be represented in our results utilizing cell-line drug responses. MYC oncogene represents a candidate driver of the s1 subtype. While MYC had been traditionally regarded as undruggable, in recent years compounds directly or indirectly inhibiting MYC have shown anticancer activity preclinically, with some of these being developed for clinical trial evaluation.[35] Consistent with our study's drug response associations, therapeutic approaches for bromodomain inhibition in cancers characterized by MYC activation are being explored.[30] Our s2 subtype showed coordinate expression of several genes in the prostaglandin synthesis pathway. COX-2-derived PGE2 supports epithelial tumor aggressiveness by several mechanisms,[36] and COX-2 selective inhibitors have been explored as a drug for cancer prevention and treatment and found to decrease the incidence of certain malignancies.[27] Regarding the s3 subtype, there is high interest in targeting EZH2 for cancer therapy. Different types of EZH2 inhibitors are under evaluation in ongoing clinical trials involving different cancer types.[37] Based on our results, BCL2 inhibitors may also target the s3 subtype. Regarding the s4 subtype, the recent clinical success of immune checkpoint inhibitors created a class of anticancer drugs to treat various malignancies.[38] One of the current challenges in cancer immunotherapy is developing biomarker panels that distinguish likely responders from non-responders, as markers such as PD-L1 represent continuous rather than discrete variables.[39] Our findings suggest that no single therapeutic approach would be effective for all cancers but that the gene expression profile and associated molecular subtype of the tumor could help maximize precision medicine approaches.

Limitations of the study

The number of discoverable pan-cancer subtypes would depend in part on the datasets examined and the analytical approaches used. Future studies examining additional datasets and cancer types might uncover additional subtypes. Subtypes of metastases within a given tissue-based cancer type could also be explored, where subtypes uniquely applicable to a given tissue of origin might be found. This study does not establish our pan-cancer subtypes as directly related to the actual processes of metastasis. The metastatic cascade represents a multistep process, with each step being explored in other studies using suitably tailored experimental model systems. In isolation, our PDX-based pan-cancer subtypes would not fully capture the influence of the tumor microenvironment on cancer metastases. The results of our study represent subtype-specific associations made across different datasets, and reported associations could be considered robust when spanning multiple molecular modalities and datasets. Still, additional directed functional experiments might be needed to establish a particular association of interest more firmly or to gain more insight.

STAR★METHODS

Detailed methods are provided in the online version of this paper and include the following:

- KEY RESOURCES TABLE
- RESOURCE AVAILABILITY
  - Lead contact
  - Materials availability
  - Data and code availability
- EXPERIMENTAL MODEL AND SUBJECT DETAILS
  - Human data
  - Cell line data
  - PDX data
- METHOD DETAILS
  - Patient tumor metastasis compendium datasets
  - PDX expression datasets
  - Pan-cancer molecular subtype discovery
  - Differential expression analyses
  - Gene signature analyses
  - Enrichment analyses for TF bound genes
  - Comparisons of orthogonal subtype-associated gene sets
  - Drug response associations
- QUANTIFICATION AND STATISTICAL ANALYSIS

SUPPLEMENTAL INFORMATION

Supplemental information can be found online at https://doi.org/10.1016/j.xcrm.2023.100932.

ACKNOWLEDGMENTS

This work was supported by National Institutes of Health (NIH) grant P30CA125123 (C.J.C.). This article was prepared using a limited access dataset obtained from BC Cancer. We thank Erin Pleasance, Laura Williamson, and Emma Titmuss for reviewing and commenting on the manuscript.

AUTHOR CONTRIBUTIONS

Conceptualization, C.J.C.; methodology, C.J.C., Y.Z., and F.C.; formal analysis, C.J.C., Y.Z., and F.C.; data curation, Y.Z. and C.J.C.; visualization, C.J.C.; writing, C.J.C.; manuscript review, Y.Z. and F.C.; supervision, C.J.C.

(C) Taking the set of 1,670 genes noted in (B), involving significant gene set overlaps between expression differences and copy gain enrichment patterns for s1 and s3 subtypes, copy gain patterns and differential expression patterns are represented in PDX compendium, patient metastasis compendium, and TCGA datasets. CNA, copy number alteration; gain, estimated gene copy number between 3 and 5; amplification, estimated gene copy number >5. Example genes listed (red, s1 genes; blue, s3 genes) are well-established cancer-associated genes by COSMIC.[24]

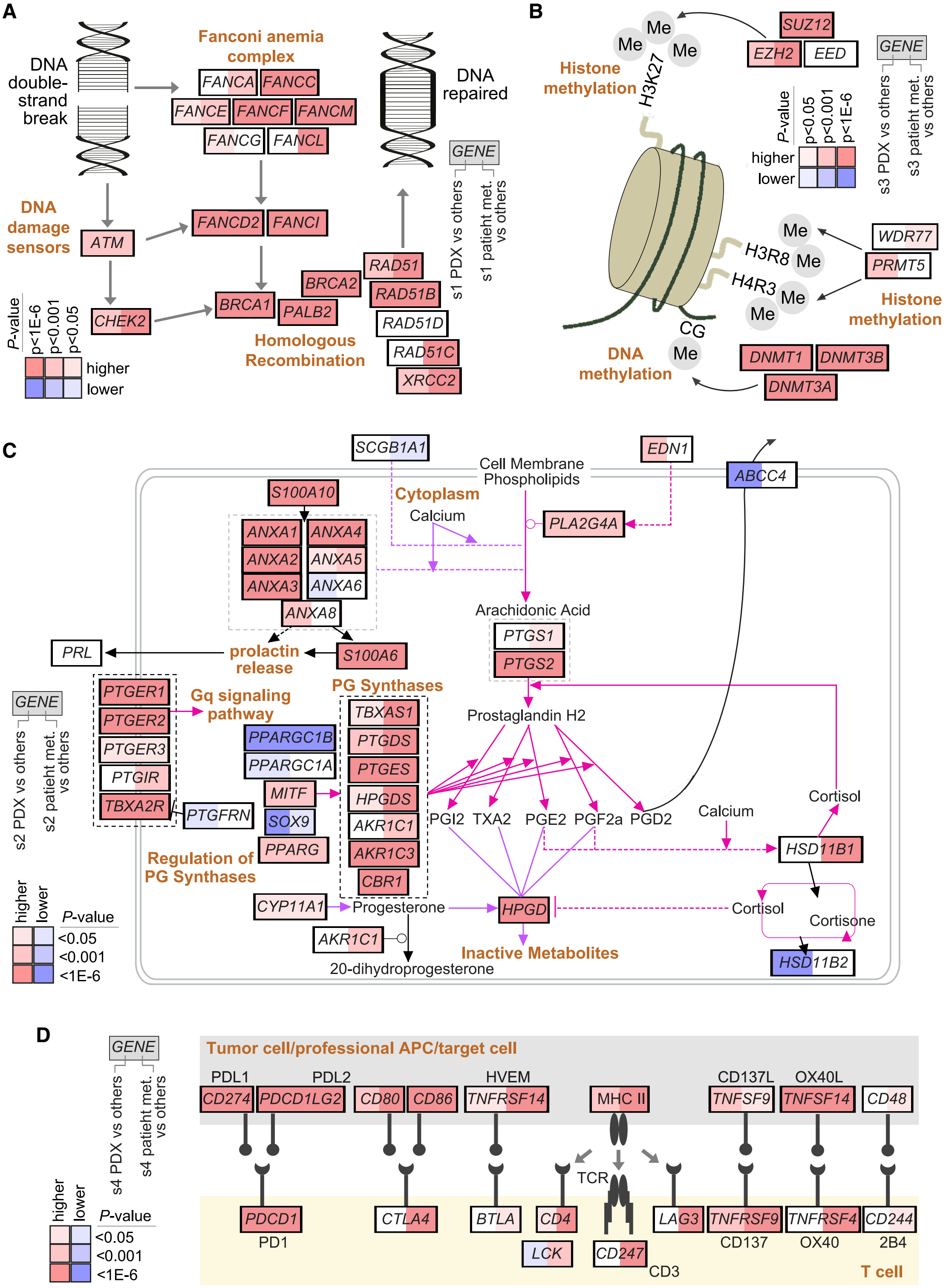

Figure 6. Pathways associated with the molecular subtypes of metastasis

(A) Diagram of key genes involved in the DNA double-strand-break repair pathway,[25] with differential expression patterns represented in both PDX and patient tumor metastasis compendiums, comparing s1 with the rest of the tumors (red, significantly higher in s1).

(B) Diagram of key genes involved in methylation of DNA and histones,[25] with differential expression patterns represented in both PDX and patient tumor metastasis compendiums, comparing s3 with the rest of the tumors (red, significantly higher in s3).

(legend continued on next page)

DECLARATION OF INTERESTS

C.J.C. recently served on a scientific advisory board/consultancy for Roche Diagnostics.

Received: October 7, 2022
Revised: November 22, 2022
Accepted: January 12, 2023
Published: February 1, 2023

REFERENCES

1. Gupta, G.P., and Massagué, J. (2006). Cancer metastasis: building a framework. Cell *127*, 679–695.
2. Dillekås, H., Rogers, M.S., and Straume, O. (2019). Are 90% of deaths from cancer caused by metastases? Cancer Med. *8*, 5574–5576.
3. Hanahan, D., and Weinberg, R.A. (2000). The hallmarks of cancer. Cell *100*, 57–70.
4. Chaffer, C.L., and Weinberg, R.A. (2011). A perspective on cancer cell metastasis. Science *331*, 1559–1564.
5. Robinson, D.R., Wu, Y.M., Lonigro, R.J., Vats, P., Cobain, E., Everett, J., Cao, X., Rabban, E., Kumar-Sinha, C., Raymond, V., et al. (2017). Integrative clinical genomics of metastatic cancer. Nature *548*, 297–303.
6. Pleasance, E., Titmuss, E., Williamson, L., Kwan, H., Culibrk, L., Zhao, E.Y., Dixon, K., Fan, K., Bowlby, R., Jones, M.R., et al. (2020). Pan-cancer analysis of advanced patient tumors reveals interactions between therapy and genomic landscapes. Nat. Cancer *1*, 452–468.
7. Cancer Genome Atlas Network (2012). Comprehensive molecular portraits of human breast tumours. Nature *490*, 61–70. https://doi.org/10.1038/nature11412.
8. Chen, F., Zhang, Y., Gibbons, D.L., Deneen, B., Kwiatkowski, D.J., Ittmann, M., and Creighton, C.J. (2018). Pan-cancer molecular classes transcending tumor lineage across 32 cancer types, multiple data platforms, and over 10,000 cases. Clin. Cancer Res. *24*, 2182–2193.
9. Chen, F., Zhang, Y., Varambally, S., and Creighton, C.J. (2019). Molecular correlates of metastasis by systematic pan-cancer analysis across the cancer genome atlas. Mol. Cancer Res. *17*, 476–487.
10. Aran, D., Sirota, M., and Butte, A.J. (2015). Systematic pan-cancer analysis of tumour purity. Nat. Commun. *6*, 8971.
11. Isella, C., Brundu, F., Bellomo, S.E., Galimi, F., Zanella, E., Porporato, R., Petti, C., Fiori, A., Orzan, F., Senetta, R., et al. (2017). Selective analysis of cancer-cell intrinsic transcriptional traits defines novel clinically relevant subtypes of colorectal cancer. Nat. Commun. *8*, 15107.
12. Alzubi, M.A., Turner, T.H., Olex, A.L., Sohal, S.S., Tobin, N.P., Recio, S.G., Bergh, J., Hatschek, T., Parker, J.S., Sartorius, C.A., et al. (2019). Separation of breast cancer and organ microenvironment transcriptomes in metastases. Breast Cancer Res. *21*, 36.
13. Creighton, C.J., Bromberg-White, J.L., Misek, D.E., Monsma, D.J., Brichory, F., Kuick, R., Giordano, T.J., Gao, W., Omenn, G.S., Webb, C.P., and Hanash, S.M. (2005). Analysis of tumor-host interactions by gene expression profiling of lung adenocarcinoma xenografts identifies genes involved in tumor formation. Mol. Cancer Res. *3*, 119–129.
14. Creighton, C., Kuick, R., Misek, D.E., Rickman, D.S., Brichory, F.M., Rouillard, J.-M., Omenn, G.S., and Hanash, S. (2003). Profiling of pathway-specific changes in gene expression following growth of human cancer cell lines transplanted into mice. Genome Biol. *4*, R46.
15. Chen, F., Zhang, Y., Bossé, D., Lalani, A.K.A., Hakimi, A.A., Hsieh, J.J., Choueiri, T.K., Gibbons, D.L., Ittmann, M., and Creighton, C.J. (2017). Pan-urologic cancer genomic subtypes that transcend tissue of origin. Nat. Commun. *8*, 199.
16. Zhang, Y., Chen, F., Chandrashekar, D.S., Varambally, S., and Creighton, C.J. (2022). Proteogenomic characterization of 2002 human cancers reveals pan-cancer molecular subtypes and associated pathways. Nat. Commun. *13*, 2669.
17. Chen, F., Chandrashekar, D.S., Varambally, S., and Creighton, C.J. (2019). Pan-cancer molecular subtypes revealed by mass-spectrometry-based proteomic characterization of more than 500 human cancers. Nat. Commun. *10*, 5679.
18. Klebe, M., Fremd, C., Kriegsmann, M., Kriegsmann, K., Albrecht, T., Thewes, V., Kirchner, M., Charoentong, P., Volk, N., Haag, J., et al. (2020). Frequent molecular subtype switching and gene expression alterations in lung and pleural metastasis from luminal A-type breast cancer. JCO Precis. Oncol. *4*. PO.19.00337.
19. Farach, A., Ding, Y., Lee, M., Creighton, C., Delk, N.A., Ittmann, M., Miles, B., Rowley, D., Farach-Carson, M.C., and Ayala, G.E. (2016). Neuronal trans-differentiation in prostate cancer cells. Prostate *76*, 1312–1325.
20. FANTOM Consortium and the RIKEN PMI and CLST DGT; Forrest, A.R.R., Kawaji, H., Rehli, M., Baillie, J.K., de Hoon, M.J.L., Haberle, V., Lassmann, T., Kulakovskiy, I.V., Lizio, M., et al. (2014). A promoter-level mammalian expression atlas. Nature *507*, 462–470.
21. ENCODE Project Consortium (2012). An integrated encyclopedia of DNA elements in the human genome. Nature *489*, 57–74.
22. Hurley, D., Araki, H., Tamada, Y., Dunmore, B., Sanders, D., Humphreys, S., Affara, M., Imoto, S., Yasuda, K., Tomiyasu, Y., et al. (2012). Gene network inference and visualization tools for biologists: application to new human transcriptome datasets. Nucleic Acids Res. *40*, 2377–2398.
23. Zhang, Y., Chen, F., Pleasance, E., Williamson, L., Grisdale, C.J., Titmuss, E., Laskin, J., Jones, S.J.M., Cortes-Ciriano, I., Marra, M.A., and Creighton, C.J. (2021). Rearrangement-mediated cis-regulatory alterations in advanced patient tumors reveal interactions with therapy. Cell Rep. *37*, 110023.
24. Forbes, S.A., Beare, D., Boutselakis, H., Bamford, S., Bindal, N., Tate, J., Cole, C.G., Ward, S., Dawson, E., Ponting, L., et al. (2017). COSMIC: somatic cancer genetics at high-resolution. Nucleic Acids Res. *45*, D777–D783.
25. Zhang, Y., Yang, L., Kucherlapati, M., Hadjipanayis, A., Pantazi, A., Bristow, C.A., Lee, E.A., Mahadeshwar, H.S., Tang, J., Zhang, J., et al. (2019). Global impact of somatic structural variation on the DNA methylome of human cancers. Genome Biol. *20*, 209.
26. Slenter, D.N., Kutmon, M., Hanspers, K., Riutta, A., Windsor, J., Nunes, N., Mélius, J., Cirillo, E., Coort, S.L., Digles, D., et al. (2018). WikiPathways: a multifaceted pathway database bridging metabolomics to other omics research. Nucleic Acids Res. *46*, D661–D667.
27. Wang, D., and Dubois, R.N. (2006). Prostaglandins and cancer. Gut *55*, 115–122.
28. Chen, F., Zhang, Y., Şenbabaoğlu, Y., Ciriello, G., Yang, L., Reznik, E., Shuch, B., Micevic, G., De Velasco, G., Shinbrot, E., et al. (2016). Multilevel genomics-based taxonomy of renal cell carcinoma. Cell Rep. *14*, 2476–2489.
29. Iorio, F., Knijnenburg, T.A., Vis, D.J., Bignell, G.R., Menden, M.P., Schubert, M., Aben, N., Gonçalves, E., Barthorpe, S., Lightfoot, H., et al. (2016). A landscape of pharmacogenomic interactions in cancer. Cell *166*, 740–754.

(C) Diagram of prostaglandin synthesis and regulation pathway,[26] with differential expression patterns represented in both PDX and patient tumor metastasis compendiums, comparing s2 with the rest of the tumors (red, significantly higher in s2).
(D) Diagram of immune checkpoint pathway (featuring interactions between T cells and antigen-presenting cells, including tumor cells),[8] with differential expression patterns represented in both PDX and patient tumor metastasis compendiums, comparing s4 with the rest of the tumors (red, significantly higher in s4). The p values in (A)–(D) were found by t test.

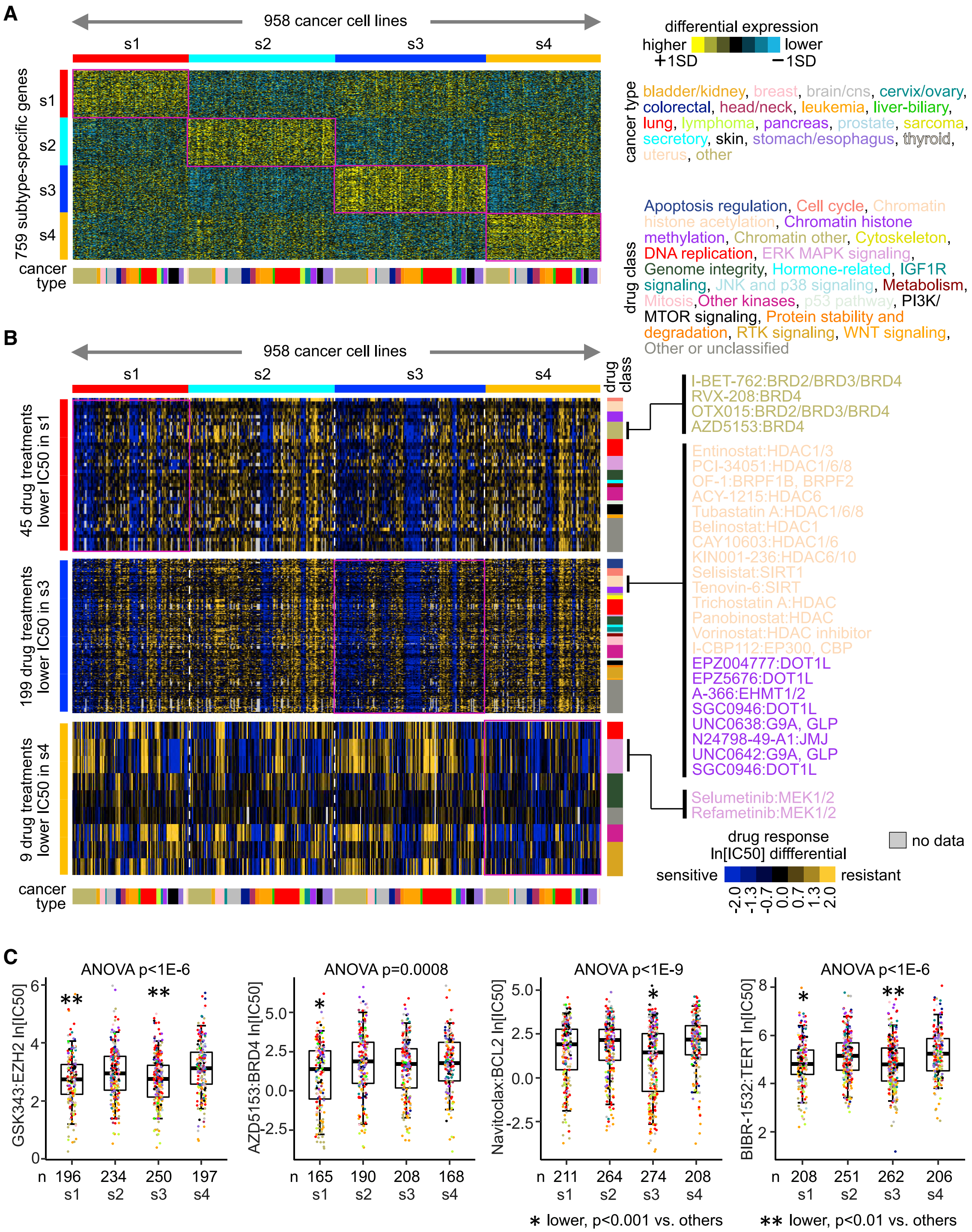

Figure 7. Molecular subtype associations with drug response in cancer cell lines

(A) Transcriptional profiles of 958 cancer cell lines represented in the Genomics of Drug Sensitivity in Cancer (GDSC)[29] dataset (profiles being normalized within their respective cancer type) were classified according to PDX-based molecular subtype. Expression patterns in cell lines for the top set of 800 mRNAs distinguishing between the four PDX-based subtypes (from Figure 1A) are shown (759 of the 800 genes being represented in GDSC).

(B) From the GDSC cell lines classified according to metastasis subtype, drug compound treatments with decreases in half-maximal inhibitory concentration (IC_{50}) associated with s1, s3, or s4 subtypes ($p < 0.001$, comparing cell lines of the given subtype with the rest of the cell lines, t test on natural log-transformed IC_{50} values). Selected drug compounds are listed by name.

(C) For selected drug compounds, natural log IC_{50}s by molecular subtype. Boxplots represent 5% (lower whisker), 25% (lower box), 50% (median), 75% (upper box), and 95% (upper whisker). Data points are colored according to cancer type, according to color coding in (A).

CellPress
OPEN ACCESS

30. Delmore, J.E., Issa, G.C., Lemieux, M.E., Rahl, P.B., Shi, J., Jacobs, H.M., Kastritis, E., Gilpatrick, T., Paranal, R.M., Qi, J., et al. (2011). BET bromodomain inhibition as a therapeutic strategy to target c-Myc. Cell *146*, 904–917.

31. Zhang, J., Tang, P., Zou, L., Zhang, J., Chen, J., Yang, C., He, G., Liu, B., Liu, J., Chiang, C.M., et al. (2021). Discovery of novel dual-target inhibitor of bromodomain-containing protein 4/casein kinase 2 inducing apoptosis and autophagy-associated cell death for triple-negative breast cancer therapy. J. Med. Chem. *64*, 18025–18053.

32. Stewart, M.L., Tamayo, P., Wilson, A.J., Wang, S., Chang, Y.M., Kim, J.W., Khabele, D., Shamji, A.F., and Schreiber, S.L. (2015). KRAS genomic status predicts the sensitivity of ovarian cancer cells to decitabine. Cancer Res. *75*, 2897–2906.

33. Li, Y., Li, H., Yao, G., Li, W., Wang, F., Jiang, Z., and Li, M. (2007). Inhibition of telomerase RNA (hTR) in cervical cancer by adenovirus-delivered siRNA. Cancer Gene Ther. *14*, 748–755.

34. van 't Veer, L.J., Dai, H., van de Vijver, M.J., He, Y.D., Hart, A.A.M., Mao, M., Peterse, H.L., van der Kooy, K., Marton, M.J., Witteveen, A.T., et al. (2002). Gene expression profiling predicts clinical outcome of breast cancer. Nature *415*, 530–536.

35. Duffy, M.J., O'Grady, S., Tang, M., and Crown, J. (2021). MYC as a target for cancer treatment. Cancer Treat Rev. *94*, 102154.

36. Finetti, F., Travelli, C., Ercoli, J., Colombo, G., Buoso, E., and Trabalzini, L. (2020). Prostaglandin E2 and cancer: insight into tumor progression and immunity. Biology *9*, 434.

37. Duan, R., Du, W., and Guo, W. (2020). EZH2: a novel target for cancer treatment. J. Hematol. Oncol. *13*, 104.

38. Ottaviano, M., De Placido, S., and Ascierto, P.A. (2019). Recent success and limitations of immune checkpoint inhibitors for cancer: a lesson from melanoma. Virchows Arch. *474*, 421–432.

39. Hegde, P.S., and Chen, D.S. (2020). Top 10 challenges in cancer immunotherapy. Immunity *52*, 17–35.

40. Monzon, F.A., Lyons-Weiler, M., Buturovic, L.J., Rigl, C.T., Henner, W.D., Sciulli, C., Dumur, C.I., Medeiros, F., and Anderson, G.G. (2009). Multicenter validation of a 1,550-gene expression profile for identification of tumor tissue of origin. J. Clin. Oncol. *27*, 2503–2508.

41. Wuttig, D., Baier, B., Fuessel, S., Meinhardt, M., Herr, A., Hoefling, C., Toma, M., Grimm, M.O., Meye, A., Rolle, A., and Wirth, M.P. (2009). Gene signatures of pulmonary metastases of renal cell carcinoma reflect the disease-free interval and the number of metastases per patient. Int. J. Cancer *125*, 474–482.

42. López-Lago, M.A., Thodima, V.J., Guttapalli, A., Chan, T., Heguy, A., Molina, A.M., Reuter, V.E., Motzer, R.J., and Chaganti, R.S.K. (2010). Genomic deregulation during metastasis of renal cell carcinoma implements a myofibroblast-like program of gene expression. Cancer Res. *70*, 9682–9692.

43. Hörnberg, E., Ylitalo, E.B., Crnalic, S., Antti, H., Stattin, P., Widmark, A., Bergh, A., and Wikström, P. (2011). Expression of androgen receptor splice variants in prostate cancer bone metastases is associated with castration-resistance and short survival. PLoS One *6*, e19059.

44. Chen, G., Chakravarti, N., Aardalen, K., Lazar, A.J., Tetzlaff, M.T., Wubbenhorst, B., Kim, S.B., Kopetz, S., Ledoux, A.A., Gopal, Y.N.V., et al. (2014). Molecular profiling of patient-matched brain and extracranial melanoma metastases implicates the PI3K pathway as a therapeutic target. Clin. Cancer Res. *20*, 5537–5546.

45. Kim, S.K., Kim, S.Y., Kim, J.H., Roh, S.A., Cho, D.H., Kim, Y.S., and Kim, J.C. (2014). A nineteen gene-based risk score classifier predicts prognosis of colorectal cancer patients. Mol. Oncol. *8*, 1653–1666.

46. Jilaveanu, L.B., Parisi, F., Barr, M.L., Zito, C.R., Cruz-Munoz, W., Kerbel, R.S., Rimm, D.L., Bosenberg, M.W., Halaban, R., Kluger, Y., and Kluger, H.M. (2015). PLEKHA5 as a biomarker and potential mediator of melanoma brain metastasis. Clin. Cancer Res. *21*, 2138–2147.

47. Tarabichi, M., Saiselet, M., Trésallet, C., Hoang, C., Larsimont, D., Andry, G., Maenhaut, C., and Detours, V. (2015). Revisiting the transcriptional analysis of primary tumours and associated nodal metastases with enhanced biological and statistical controls: application to thyroid cancer. Br. J. Cancer *112*, 1665–1674.

48. Wang, X., Dubuc, A.M., Ramaswamy, V., Mack, S., Gendoo, D.M.A., Remke, M., Wu, X., Garzia, L., Luu, B., Cavalli, F., et al. (2015). Medulloblastoma subgroups remain stable across primary and metastatic compartments. Acta Neuropathol. *129*, 449–457.

49. Haider, M., Zhang, X., Coleman, I., Ericson, N., True, L.D., Lam, H.M., Brown, L.G., Ketchanji, M., Nghiem, B., Lakely, B., et al. (2016). Epithelial mesenchymal-like transition occurs in a subset of cells in castration resistant prostate cancer bone metastases. Clin. Exp. Metastasis *33*, 239–248.

50. Iwamoto, T., Niikura, N., Ogiya, R., Yasojima, H., Watanabe, K.I., Kanbayashi, C., Tsuneizumi, M., Matsui, A., Fujisawa, T., Iwasa, T., et al. (2019). Distinct gene expression profiles between primary breast cancers and brain metastases from pair-matched samples. Sci. Rep. *9*, 13343.

51. Labrecque, M.P., Coleman, I.M., Brown, L.G., True, L.D., Kollath, L., Lakely, B., Nguyen, H.M., Yang, Y.C., da Costa, R.M.G., Kaipainen, A., et al. (2019). Molecular profiling stratifies diverse phenotypes of treatment-refractory metastatic castration-resistant prostate cancer. J. Clin. Invest. *129*, 4492–4505.

52. Kamal, Y., Schmit, S.L., Hoehn, H.J., Amos, C.I., and Frost, H.R. (2019). Transcriptomic differences between primary colorectal adenocarcinomas and distant metastases reveal metastatic colorectal cancer subtypes. Cancer Res. *79*, 4227–4241.

53. Hu, Y., Taylor-Harding, B., Raz, Y., Haro, M., Recouvreux, M.S., Taylan, E., Lester, J., Millstein, J., Walts, A.E., Karlan, B.Y., and Orsulic, S. (2020). Are epithelial ovarian cancers of the mesenchymal subtype actually intraperitoneal metastases to the ovary? Front. Cell Dev. Biol. *8*, 647.

54. Alfieri, S., Carenzo, A., Platini, F., Serafini, M.S., Perrone, F., Galbiati, D., Sponghini, A.P., Depenni, R., Vingiani, A., Quattrone, P., et al. (2020). Tumor biomarkers for the prediction of distant metastasis in head and neck squamous cell carcinoma. Cancers *12*, 922.

55. Mitra, S., Tiwari, K., Podicheti, R., Pandhiri, T., Rusch, D.B., Bonetto, A., Zhang, C., and Mitra, A.K. (2019). Transcriptome profiling reveals matrisome alteration as a key feature of ovarian cancer progression. Cancers *11*, 1513.

56. Garcia-Recio, S., Thennavan, A., East, M.P., Parker, J.S., Cejalvo, J.M., Garay, J.P., Hollern, D.P., He, X., Mott, K.R., Galván, P., et al. (2020). FGFR4 regulates tumor subtype differentiation in luminal breast cancer and metastatic disease. J. Clin. Invest. *130*, 4871–4887.

57. Yang, J., Lin, P., Yang, M., Liu, W., Fu, X., Liu, D., Tao, L., Huo, Y., Zhang, J., Hua, R., et al. (2021). Integrated genomic and transcriptomic analysis reveals unique characteristics of hepatic metastases and pro-metastatic role of complement C1q in pancreatic ductal adenocarcinoma. Genome Biol. *22*, 4.

58. Eide, P.W., Moosavi, S.H., Eilertsen, I.A., Brunsell, T.H., Langerud, J., Berg, K.C.G., Røsok, B.I., Bjørnbeth, B.A., Nesbakken, A., Lothe, R.A., and Sveen, A. (2021). Metastatic heterogeneity of the consensus molecular subtypes of colorectal cancer. NPJ Genom. Med. *6*, 59.

59. Cosgrove, N., Varešlija, D., Keelan, S., Elangovan, A., Atkinson, J.M., Cocchiglia, S., Bane, F.T., Singh, V., Furney, S., Hu, C., et al. (2022). Mapping molecular subtype specific alterations in breast cancer brain metastases identifies clinically relevant vulnerabilities. Nat. Commun. *13*, 514.

60. Bolstad, B.M., Irizarry, R.A., Astrand, M., and Speed, T.P. (2003). A comparison of normalization methods for high density oligonucleotide array data based on variance and bias. Bioinformatics *19*, 185–193.

61. Corso, S., Isella, C., Bellomo, S.E., Apicella, M., Durando, S., Migliore, C., Ughetto, S., D'Errico, L., Menegon, S., Moya-Rull, D., et al. (2019). A comprehensive PDX gastric cancer collection captures cancer cell-intrinsic transcriptional MSI traits. Cancer Res. *79*, 5884–5896.

62. Prasetyanti, P.R., van Hooff, S.R., van Herwaarden, T., de Vries, N., Kalloe, K., Rodermond, H., van Leersum, R., de Jong, J.H., Franitza, M., Nürnberg, P., et al. (2019). Capturing colorectal cancer inter-tumor heterogeneity in patient-derived xenograft (PDX) models. Int. J. Cancer *144*, 366–371.

63. Coppé, J.P., Mori, M., Pan, B., Yau, C., Wolf, D.M., Ruiz-Saenz, A., Brunen, D., Prahallad, A., Cornelissen-Steijger, P., Kemper, K., et al. (2019). Mapping phospho-catalytic dependencies of therapy-resistant tumours reveals actionable vulnerabilities. Nat. Cell Biol. *21*, 778–790.

64. Kita, K., Fukuda, K., Takahashi, H., Tanimoto, A., Nishiyama, A., Arai, S., Takeuchi, S., Yamashita, K., Ohtsubo, K., Otani, S., et al. (2019). Patient-derived xenograft models of non-small cell lung cancer for evaluating targeted drug sensitivity and resistance. Cancer Sci. *110*, 3215–3224.

65. Montaudon, E., Nikitorowicz-Buniak, J., Sourd, L., Morisset, L., El Botty, R., Huguet, L., Dahmani, A., Painsec, P., Nemati, F., Vacher, S., et al. (2020). PLK1 inhibition exhibits strong anti-tumoral activity in CCND1-driven breast cancer metastases with acquired palbociclib resistance. Nat. Commun. *11*, 4053.

66. Rusert, J.M., Juarez, E.F., Brabetz, S., Jensen, J., Garancher, A., Chau, L.Q., Tacheva-Grigorova, S.K., Wahab, S., Udaka, Y.T., Finlay, D., et al. (2020). Functional precision medicine identifies new therapeutic candidates for medulloblastoma. Cancer Res. *80*, 5393–5407.

67. Yanagihara, K., Iino, Y., Yokozaki, H., Kubo, T., Oda, T., Kubo, T., Komatsu, M., Sasaki, H., Ichikawa, H., Kuwata, T., et al. (2022). A comparative study of patient-derived tumor models of pancreatic ductal adenocarcinoma involving orthotopic implantation. Pathobiology *89*, 222–232.

68. Sueyoshi, K., Komura, D., Katoh, H., Yamamoto, A., Onoyama, T., Chijiwa, T., Isagawa, T., Tanaka, M., Suemizu, H., Nakamura, M., et al. (2021). Multi-tumor analysis of cancer-stroma interactomes of patient-derived xenografts unveils the unique homeostatic process in renal cell carcinomas. iScience *24*, 103322.

69. Dahlmann, M., Gambara, G., Brzezicha, B., Popp, O., Pachmayr, E., Wedeken, L., Pflaume, A., Mokritzkij, M., Gül-Klein, S., Brandl, A., et al. (2021). Peritoneal metastasis of colorectal cancer (pmCRC): identification of predictive molecular signatures by a novel preclinical platform of matching pmCRC PDX/PD3D models. Mol. Cancer *20*, 129.

70. Mao, N., Zhang, Z., Lee, Y.S., Choi, D., Rivera, A.A., Li, D., Lee, C., Haywood, S., Chen, X., Chang, Q., et al. (2021). Defining the therapeutic selective dependencies for distinct subtypes of PI3K pathway-altered prostate cancers. Nat. Commun. *12*, 5053.

71. Dong, X., Xue, H., Mo, F., Lin, Y.Y., Lin, D., Wong, N.K.Y., Sun, Y., Wilkinson, S., Ku, A.T., Hao, J., et al. (2022). Modeling androgen deprivation therapy-induced prostate cancer dormancy and its clinical implications. Mol. Cancer Res. *20*, 782–793.

72. Sun, H., Cao, S., Mashl, R.J., Mo, C.K., Zaccaria, S., Wendl, M.C., Davies, S.R., Bailey, M.H., Primeau, T.M., Hoog, J., et al. (2021). Comprehensive characterization of 536 patient-derived xenograft models prioritizes candidatesfor targeted treatment. Nat. Commun. *12*, 5086.

73. Chen, K., Ahmed, S., Adeyi, O., Dick, J.E., and Ghanekar, A. (2012). Human solid tumor xenografts in immunodeficient mice are vulnerable to lymphomagenesis associated with Epstein-Barr virus. PLoS One *7*, e39294.

74. Bindea, G., Mlecnik, B., Tosolini, M., Kirilovsky, A., Waldner, M., Obenauf, A.C., Angell, H., Fredriksen, T., Lafontaine, L., Berger, A., et al. (2013). Spatiotemporal dynamics of intratumoral immune cells reveal the immune landscape in human cancer. Immunity *39*, 782–795.

75. Wilkerson, M.D., and Hayes, D.N. (2010). ConsensusClusterPlus: a class discovery tool with confidence assessments and item tracking. Bioinformatics *26*, 1572–1573.

76. Storey, J.D., and Tibshirani, R. (2003). Statistical significance for genomewide studies. Proc. Natl. Acad. Sci. USA *100*, 9440–9445.

77. Ashburner, M., Ball, C.A., Blake, J.A., Botstein, D., Butler, H., Cherry, J.M., Davis, A.P., Dolinski, K., Dwight, S.S., Eppig, J.T., et al. (2000). Gene ontology: tool for the unification of biology. The Gene Ontology Consortium. Nat. Genet. *25*, 25–29.

78. Creighton, C.J., Nagaraja, A.K., Hanash, S.M., Matzuk, M.M., and Gunaratne, P.H. (2008). A bioinformatics tool for linking gene expression profiling results with public databases of microRNA target predictions. RNA *14*, 2290–2296.

79. Saldanha, A.J. (2004). Java Treeview–extensible visualization of microarray data. Bioinformatics *20*, 3246–3248.

80. Pavlidis, P., and Noble, W.S. (2003). Matrix2png: a utility for visualizing matrix data. Bioinformatics *19*, 295–296.

CellPress
OPEN ACCESS

STAR★METHODS

KEY RESOURCES TABLE

REAGENT or RESOURCE	SOURCE	IDENTIFIER
Deposited data		
POG570 mutation and expression datasets	Canada's Michael Smith Genome Sciences Center (GSC) at BC Cancer	http://bcgsc.ca/downloads/POG570/
MET500 mutation and expression datasets	University of Michigan	https://met500.med.umich.edu/datasets
Count Me In (CMI): The Metastatic Breast Cancer (MBC) Project expression datasets	Genome Data Commons (GDC)	https://portal.gdc.cancer.gov/projects/CMI-MBC
GEO patient metastases expression datasets	Gene Expression Omnibus (GEO)	GEO: GSE12630, GEO: GSE14378, GEO: GSE18549, GEO: GSE23629, GEO: GSE29650, GEO: GSE50493, GEO: GSE50760, GEO: GSE60464, GEO: GSE60542, GEO: GSE63668, GEO: GSE74685, GEO: GSE125989, GEO: GSE126078, GEO: GSE131418, GEO: GSE133296, GEO: GSE136037, GEO: GSE137237, GEO: GSE147322, GEO: GSE151580, GEO: GSE159216, GEO: GSE184869
NIH-NCI PDX Development and Trial Centers Research Network (PDXNet)/ NCI Patient-Derived Models Repository (PDMR) mutation and expression datasets	National Cancer Institute (NCI)	https://doi.org/10.6084/m9.figshare.14390408
GEO patient-derived xenograft (PDX) expression datasets	Gene Expression Omnibus (GEO)	GEO: GSE76402, GEO: GSE98708, GEO: GSE103340, GEO: GSE118942, GEO: GSE128459, GEO: GSE129127, GEO: GSE130160, GEO: GSE146661, GEO: GSE151343, GEO: GSE157494, GEO: GSE159702, GEO: GSE180790, GEO: GSE181374, GEO: GSE193500
The Cancer Genome Atlas (TCGA) mutation and expression datasets	Broad Institute	https://gdac.broadinstitute.org/
FANTOM5 cell and tissue expression dataset	RIKEN	http://fantom.gsc.riken.jp/5/data/
ENCODE Transcription Factor (TF) binding	Ensembl Biomart (GRCh37/hg19 build)	https://grch37.ensembl.org/info/data/biomart/index.html
Gene expression profiles of 400 siRNA knocked down on HUVEC	Gene Expression Omnibus (GEO)	GEO: GSE27869
Genomics of Drug Sensitivity in Cancer (GDSC) cell line datasets	Wellcome Sanger Institute	https://www.cancerrxgene.org/
Software and algorithms		
ConsensusClusterPlus (v3.16)	Bioconductor	https://www.bioconductor.org/packages/release/bioc/html/ConsensusClusterPlus.html
SigTerms (v1.0)	Baylor College of Medicine	https://sigterms.sourceforge.net/

RESOURCE AVAILABILITY

Lead contact

Further information and requests for resources and reagents should be directed to and will be fulfilled by the lead contact, Chad J. Creighton (creighto@bcm.edu).

Materials availability

This study did not generate new, unique reagents.

Data and code availability

- This paper analyzes existing, publicly available data. Details on accessing the datasets are listed in the key resources table. The compendium datasets of gene expression profiles for PDX, patient metastases, and paired patient metastasis with primary—compiled as part of our study—are available through GitHub [https://github.com/chadcreighton/metastasis-expression-compendium]. Each expression dataset is uploaded on GitHub as a series of separate files by individual study, using a common gene feature set with the same ordering across files. One can concatenate the individual matrices together to assemble the compendium datasets using in this study.
- This paper does not report original code. No custom computer code was used for data collection, which was performed using open-source software. Additional processing involved in-house scripts that are available upon request. All analyses used previously published software or methods.
- Any additional information required to reanalyze the data reported in this paper is available from the lead contact upon request.

EXPERIMENTAL MODEL AND SUBJECT DETAILS

Human data

Regarding human subjects, cancer molecular profiling data were generated through informed consent as part of previously published studies and analyzed in accordance with each original study's data use guidelines and restrictions.

Cell line data

Molecular and phenotypic data on cell lines was accessed from public repositories, as described below. Details on cell maintenance and care are described in the original studies generating these data.

PDX data

Molecular data on PDX models was accessed from previously published studies, as described below. Details on PDX tumor generation and molecular characterization are described in the original studies generating these data.

METHOD DETAILS

Patient tumor metastasis compendium datasets

We assembled a compendium dataset of gene expression profiling data of metastatic tumors from 26 major cancer types (based on tissue of origin) and 24 individual studies (Table S1).[5,6,40–59] The 2405 tumors in the compendium represented 2158 patients. The above studies analyzed the tumors using global mRNA profiling by RNA-sequencing (RNA-seq) or array platform. We obtained processed expression data tables from the Gene Expression Omnibus (GEO, accession numbers included in Table S1), from websites associated with the study publication (for MET500 and POG570 datasets), or from the Genome Data Commons (in the case of the Count Me In, or CMI dataset). Where multiple studies and associated datasets came from the same research team,[49,51] we ensured that tumors were not represented more than once in the compendium, removing duplicate profiles. For RNA-seq data with raw counts data provided, we converted these to Transcripts per million (TPM) expression values. Where we observed considerable variability in the total expression values across genes among profiles, we applied quantile normalization to the individual dataset.[60] For a given dataset, in instances where genes were represented by more than one feature, the feature with the highest variability across profiles (by standard deviation applied to log2-transformed expression values) was selected to represent the gene in the compendium dataset. Without correction, widespread differences in relative gene levels observed between any two datasets would represent a combination of technical batch effects (e.g., stemming from different mRNA profiling platforms and different laboratories) and of biological differences involving tumor tissue of origin or metastasis biopsy site. To correct for both of the above, we normalized the genes within each dataset and within each cancer type (for those datasets with more than one cancer type represented) to standard deviations from the median, using log2-transformed values, similar to what we have done in previous studies.[8,15–17] This data transformation to unitless standard deviations from the median allowed for the values for a given gene to be comparable among the various datasets. As the set of genes represented in the patient tumor metastasis compendium datasets, we took the 18319 genes represented by Entrez identifier in both the MET500 and POG570 datasets (representing 934 of the 2405 tumor metastases).

Of the 2405 tumors in the above metastasis compendium expression dataset, 307 patient tumor metastases had a corresponding primary tumor pair from the same patient also being profiled. These 307 tumor metastases represented 291 patients, eight major cancer types (based on tissue of origin), and 13 studies. To carry out paired metastasis versus primary tumor comparisons, we compiled a separate metastasis compendium of the above 307 tumor metastases. To normalize the metastases profiles relative to the paired primary, we first centered log2-transformed expression values for each metastasis expression profile on its primary pair, setting the values for the primary pair to zero. Then, within each study dataset, the centered expression values were divided by the standard deviation across the centered metastasis and primary profiles. This normalization step rendered the differential expression values unitless, thereby correcting for inter-dataset differences.

Of the 2405 tumors in our patient tumor metastasis compendium dataset, 934—involving the MET500[5] and POG570[6] datasets—had DNA sequencing data yielding gene copy and small somatic mutation information. For the POG570 dataset, gene-level copy values were generated as integers representing the predicted copy number state,[23] from 0 to 5, 3–5 representing gene copy gain or amplification, correcting for tumor ploidy. For the MET500 dataset, gene-level copy values were generated as integers from 0 to 20, not correcting for tumor ploidy. We first applied a correction for ploidy to each metastatic tumor profile in the MET500 dataset, whereby each gene copy value was divided by the median gene copy value across genes (2 for most tumors) and multiplied by 2. Gene copy values greater than or equal to three for each tumor profile in the MET500 and POG570 datasets were called copy gain, while gene copy values less than two were called as copy loss.

PDX expression datasets

Analogous to the above involving the patient metastasis expression compendium dataset, we assembled a compendium dataset of PDX tumors representing over 18 major cancer types (based on tissue of origin) and 14 individual studies (Table S1).[11,12,61–71] We identified the above studies and associated expression datasets by searching the GEO database. In addition, we incorporated an expression profiling dataset from Sun et al.[72] representing 1551 PDX tumors and 536 patients and over 16 major cancer types by tissue of origin, involving the NIH-NCI PDX Development and Trial Centers Research Network (PDXNet) and the NIH-NCI Patient-Derived Models Repository (PDMR) repositories. The above studies analyzed the tumors using global mRNA profiling by RNA-sequencing (RNA-seq) or array platform. For RNA-seq data with raw counts data provided, we converted these to Transcripts per million (TPM) expression values. Where we observed considerable variability in the total expression values across genes among profiles, we applied quantile normalization to the individual dataset.[60] For a given dataset, in instances where genes were represented by more than one feature, the feature with the highest variability across profiles (by standard deviation applied to log2-transformed expression values) was selected to represent the gene in the compendium dataset. As carried out above for the 2405-patient tumor metastases compendium, we normalized the genes within each dataset and within each cancer type (for those datasets with more than one cancer type represented) to standard deviations from the median, using log2-transformed values. We obtained the PDXNet/PDMR expression data matrix (TPM values) from the Sun et al. publication. We transformed log2-transformed expression values within each cancer type to standard deviations from the median to remove tissue-dominant differences. The set of 18319 genes represented in the patient tumor compendium dataset was the set represented in the PDX compendium dataset.

PDXs may be vulnerable to lymphomagenesis.[73] Therefore, we took conservative measures to remove PDX tumor profiles from our compendium that manifested strong patterns associated with lymphocytes. In the original study involving the GSE76402 dataset,[11] 14 samples were found to be contaminated by murine or human lymphomas and were not further considered in the analysis. Based on our analysis of these 14 sample profiles, we removed sample profiles with either B cell marker CD19 elevated at three standard deviations from the dataset median or a gene signature of B cells from Bindea et al.[74] elevated at three standard deviations from the dataset median. Our present study does not make any definitive conclusions regarding the samples not included in the study. The final PDX compendium expression dataset consisted of 2371 tumors representing 1000 patients.

Pan-cancer molecular subtype discovery

We used the PDX compendium expression dataset to identify molecular subtypes, which we then examined in other expression datasets. ConsensusClusterPlus R-package[75] (using R version 4.1.1) was used to identify the structure and relationship of the samples. For unsupervised clustering analysis, we randomly selected 2000 genes represented in at least 2300 of the 2371 tumor profiles of the PDX compendium dataset. Consensus ward linkage hierarchical clustering identified k = 2 to k = 15 subtypes, with the stability of the clustering increasing with increasing k. We considered multiple subtype solutions, as described in Figure S2. Beyond a 7-subtype solution, additional subtypes identified involved relatively fewer samples and were not well represented in both GEO and PDXNet/PDMR compendium subsets. In exploring the 7-subtype solution further, three of these subtypes had samples represented almost entirely in either GEO datasets or the PDXNet/PDMR subsets but not both, where we sought robust subtype associations involving multiple datasets. Therefore, we reclassified the profiles in k subtypes 5–7 according to the best fit among subtypes 1–4 to arrive at the final 4-subtype solution (s1 through s4). For this reclassification, we determined the top 200 gene correlates for each of the four subtypes. For each subtype, we assigned either "1" if the gene was a top 200 gene for the given subtype and "0" if otherwise. We then computed the Pearson correlation between each PDX subtype classifier and the sample profiles to be reclassified. We assigned each reclassified tumor profile to one of the four subtypes, based on which subtype classifier showed the highest correlation with the given external dataset profile.

Based on the set of subtypes derived from our PDX compendium expression dataset, we examined expression profiling datasets external to the PDX compendium, classifying each external tumor profile by PDX-based subtype. We classified tumors in the patient tumor metastasis compendium and TCGA pan-cancer datasets by PDX-based subtype. Within each cancer type of TCGA dataset (by TCGA project), we normalized log-transformed mRNAs to standard deviations from the median. As a classifier, we used the top set of 800 genes distinguishing between the PDX-based subtypes based on analysis of the PDX compendium (200 genes for each of the four subtypes, based on all 2371 PDX tumors). To define the top over-expressed genes for each subtype, we first compared PDX tumors of the given subtype with the rest of the tumors by t-test. For a given subtype, a top gene had the highest differential expression by t-statistic compared to the other subtypes and a higher t-statistic than the other genes that did not make the top list. As the classifier for each subtype, we assigned the 800 genes "1" if gene was a top 200 gene for the given subtype and "0" if otherwise. We

then computed the Pearson correlation between each external profile and each PDX subtype classifier. We assigned each external tumor profile to a PDX-based subtype, based on which subtype classifier showed the highest correlation with the given external dataset profile. We similarly classified tumor profiles according to our previously identified TCGA-based pan-cancer subtypes8. Taking the previously defined 854 mRNAs distinguishing between TCGA-based subtypes as the subtype classifier, we assigned to each gene "1" or "-1" for up versus down, respectively, if the gene was a top 100 gene for the given subtype and "0" if otherwise. Tumor profiles that did not significantly align with TCGA-based c1 or c3-c10 subtypes with a significance of $p < 0.05$ (Pearson's correlation) were assigned to the nondescript "c2" subtype.

For TCGA and cell line datasets, proteomic data by mass spectrometry-based platform or by reverse-phase protein array (RPPA) platform were available. To compare with the results of the mRNA-based classification (these mRNA-based classifications being used for downstream analyses), we classified tumors and cell lines based on available proteomic data, as presented in Figure S4. Log2-transformed protein expression values (by either mass spectrometry-based or RPPA platform) were centered to standard deviations from the median within each cancer type. For the mass spectrometry-based datasets, we used as the classifier the top 800 subtype-specific genes from the PDX dataset (Figure 1A). For the TCGA RPPA dataset, we used as a classifier the set of represented total protein features from which a significant association with a particular subtype was observable in the PDX compendium dataset ($p < 0.001$ by t-test, based on logged and centered protein expression values).

Differential expression analyses

We assessed differential expression between comparison groups using t-tests on expression values log2-transformed and normalized within each dataset and cancer type as described above. Differential gene sets greatly exceeded the estimated chance expected by multiple testing of 18319 genes, using the method of Storey and Tibshirani.[76] We applied a nominal p-value cutoff to each gene when comparing subtype-specific patterns based on multiple criteria. We used this rather than a stringent false discovery rate cutoff to lower false negative results (while the multiple criteria would keep the false positive rate due to multiple gene testing low). In defining the gene expression signature of metastasis versus paired primary within a given cancer type (Figure S1), we used the compendium of 307 metastasis samples, representing eight cancer types.

Gene signature analyses

We surveyed global expression patterns associated with cells and tissues of the CNS, using the public fantom datasets.[20] We obtained gene expression profiles from various normal human cells and tissues from the FANTOM5 data repository (http://fantom.gsc.riken.jp/5/data/). We removed profiles from fetal or embryonic human specimens from the analysis for our study. We centered log2 expression values for each gene in the fantom dataset on the median of sample profiles. For each fantom differential expression profile (genes centered within the fantom dataset), we took the inter-profile correlation (Pearson's) with that of the differential expression profile for each PDX and patient tumor metastasis (with the genes in each compendium centered and normalized as described above).

For gene signatures of gene knockdown, we referred to the GSE27869 expression profile dataset of human umbilical vein endothelial cells (HUVECs) transfected with siRNAs for 400 different genes.[22] We normalized log2 gene expression values in GSE27869 to standard deviations from the median across the 400 profiles. Of the 400 genes represented in GSE27869, 44 involved the 158 TFs surveyed using Encode data (see below). For each siRNA differential expression profile, we took the inter-profile correlation (Pearson's) with that of the differential expression profile for each PDX and patient tumor metastasis. We then compared the siRNA signature scoring levels among the molecular subtypes.

Enrichment analyses for TF bound genes

We obtained TF binding site locations, based on ENCODE consortium data,[21] from Ensembl (GRCh37/hg19 build). We used TF sites as identified in the HeLa-S3, HepG2, and K562 cell lines (accessed April 2022), involving 158 TFs. We defined associations between TFs and genes as a TF binding site falling within 2kb upstream of the gene start. For each TF and each PDX-based subtype, we identified patterns of significant gene set overlap (by one-sided Fisher's exact test or chi-square test) between the TF-bound genes and the genes with higher relative expression in the PDX-based subtype relative to other tumors. We separately evaluated the top genes for PDX and patient tumor metastases dataset comparisons ($p < 0.01$ unpaired t-test, with levels also highest in the given subtype compared to all other subtypes).

Comparisons of orthogonal subtype-associated gene sets

We compared subtype-associated gene sets obtained from orthogonal comparisons to identify patterns of significant gene overlap of interest. For each PDX-based subtype, we overlapped the set of genes high within that subtype versus the rest of the PDX tumors with the set of genes high in patient metastases of the same PDX-based subtype versus the corresponding paired primaries. We used a statistic cutoff of $p < 0.01$ for each gene set (PDX dataset comparisons, unpaired t-test, with levels also being highest in the given subtype compared to all other subtypes; paired patient comparisons, paired t-test). One-sided Fisher's exact or chi-square tests evaluated the significance of the overlap between the orthogonal gene sets. Analogous comparisons were carried out for the sets of genes low in each PDX-based subtype and the sets of genes lower in patient metastasis of the same subtype versus the corresponding paired primaries.

We also compared differentially expressed genes associated with a given PDX-based subtype with genes with copy gain more frequent in that subtype. Of the 2405 tumors in our patient tumor metastasis compendium dataset, 934—involving the MET500[5] and POG570[6] datasets—had gene copy information. Of the 2371 tumors in the PDX expression compendium dataset, 1238—involving the PDXNet/PDMR dataset—had gene copy information. In addition, 9853 tumors in TCGA had both gene copy and expression information. For each dataset, we assessed the frequency of gene copy gain events (i.e., three or more copies) for each gene by subtype, with significance of enrichment by one-sided Fisher's exact or chi-square test (the latter in instances where large numbers were involved). Differentially expressed genes by subtype were based on comparisons of PDX-based subtype versus the rest of the tumors. For each of the three datasets with gene copy information, chi-square tests evaluated the significance of the overlap between genes with higher expression within a given PDX-based subtype (with levels also being highest in the given subtype compared to all other subtypes) and the genes with copy gain events more frequent within each subtype.

Drug response associations

Using the Genomics of Drug Sensitivity in Cancer (GDSC)[29] resource, we classified 962 cancer cell lines according to PDX-based subtype. Of the 962 cell lines, 214 were annotated as metastatic (Table S1). GDSC expression data and drug compound half maximal inhibitory concentration (IC50) data were downloaded in February 2020 (GDSC1-dataset) and in October 2022 (GDSC2-dataset). We merged the two GDSC IC50 datasets into one. if a drug treatment and cell line were represented in both datasets, we averaged the two values; otherwise, we used whichever IC50 dataset had available data. GDSC IC50 data represented 623 drug treatments involving 544 compounds. Within each cancer type of the GDSC expression array dataset, log base 2-transformed genes were normalized to standard deviations from the median. Using the top 200 genes for each subtype as defined using the PDX compendium expression dataset, we classified the cell lines as described above (based on mRNA data). We further evaluated the cell lines for differences in IC50 drug responses according to molecular subtypes, using t-test on natural log-transformed IC50 values, comparing cell lines of the given subtypes with the rest of the cell lines.

QUANTIFICATION AND STATISTICAL ANALYSIS

All p values were two-sided unless otherwise specified. Enrichment of GO annotation terms[77] within sets of differentially expressed genes was evaluated using SigTerms software[78] and one-sided Fisher's exact tests. Visualization using heat maps was performed using both JavaTreeview (version 1.1.6r4)[79] and matrix2png (version 1.2.1).[80] Figures indicate exact value of n (number of tumors or cell lines), and the statistical tests used are noted in the Figure legends and next to reported p-values in the results section. Boxplots represent 5%, 25%, 50%, 75%, and 95%. Figures represent biological and not technical replicates.

Cell Reports Medicine, Volume *4*

Supplemental information

Pan-cancer molecular subtypes of metastasis reveal distinct and evolving transcriptional programs

Yiqun Zhang, Fengju Chen, and Chad J. Creighton

Supplementary Figures

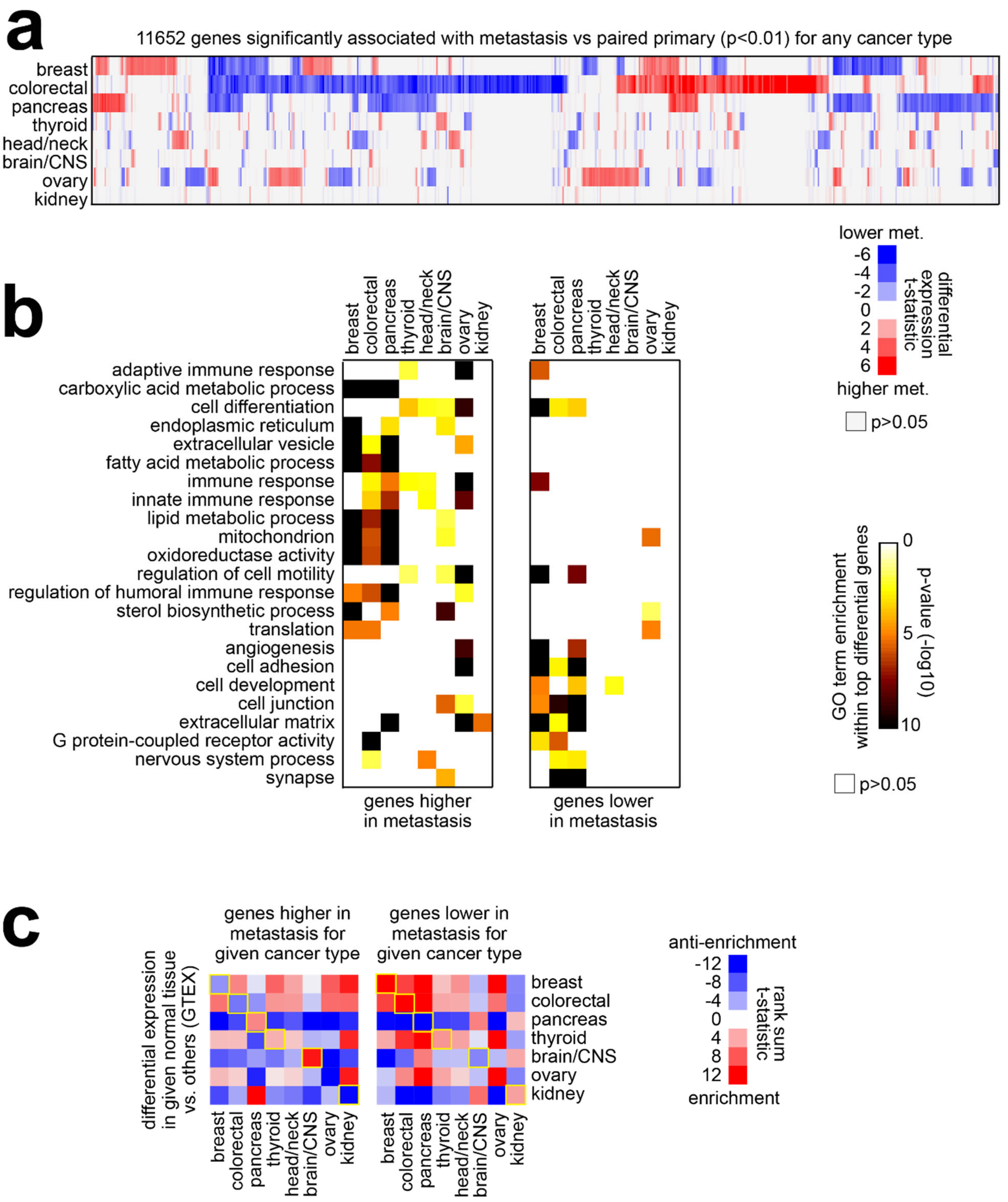

Figure S1. Gene expression signatures of metastasis versus paired primary, by cancer type. Related to STAR Methods. (a) Across eight cancer types represented in our paired metastasis and primary compendium dataset, heat map of differential t-statistics (paired t-test on log-transformed data), by cancer type, comparing metastasis versus paired primary (red, higher expression in metastasis; white, not significant with $p > 0.05$), for 11652 genes significant for any cancer type ($p<0.01$). **(b)** Significance of enrichment (by one-sided Fisher's exact test) for selected GO terms with the respective sets of genes higher or lower in metastasis versus paired primary ($p<0.01$, paired t-test) for each cancer type represented. **(c)** A likely confounder with the above metastasis versus primary expression patterns would involve differences in non-cancer cells between the primary site and the metastasis biopsy site (e.g., comparing breast versus non-breast tissues within the breast cancer dataset). Here, we used the GTEX dataset[1] to compare normal tissues of a given type with the rest of the normal tissue profiles, based on analysis of 11688 tissues. We then evaluated the overall enrichment pattern of each metastasis-associated gene set (from part a, using $p<0.05$) within each GTEX-based normal tissue differential profile (by rank sum statistic). Entries in the correlation matrix corresponding to the same tissue type between GTEX and patient metastasis compendium are highlighted in yellow. For three cancer types (breast, colorectal, and kidney), genes up in metastasis are anti-enriched within the corresponding normal tissues and genes down in metastasis are enriched within normal tissues. With breast cancer, for example, this can reflect that the metastasis sample biopsy has more non-breast cells and less breast cells as compared to the primary sample biopsy. The genes arising from the above metastasis versus primary paired comparisons were not used as the basis for defining our PDX-based molecular subtypes.

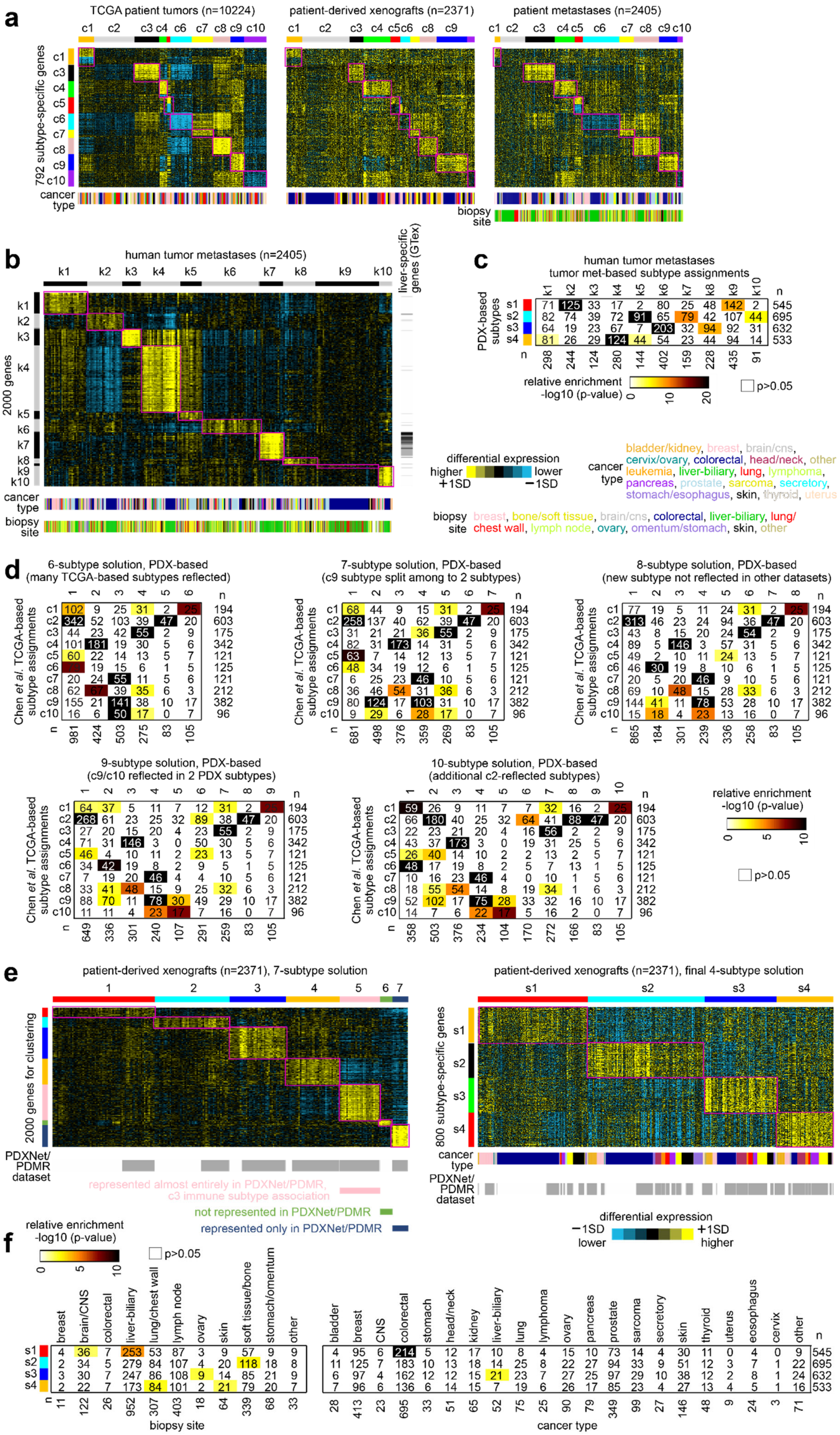

c

	k1	k2	k3	k4	k5	k6	k7	k8	k9	k10	n
s1	71	125	33	17	2	80	25	48	142	2	545
s2	82	74	39	72	91	65	79	42	107	44	695
s3	64	19	23	67	7	203	32	94	92	31	632
s4	81	26	29	124	44	54	23	44	94	14	533
n	298	244	124	280	144	402	159	228	435	91	

d — 6-subtype solution

	1	2	3	4	5	6	n
c1	102	9	25	31	2	25	194
c2	342	52	103	39	47	20	603
c3	44	23	42	55	2	9	175
c4	101	181	19	30	5	6	342
c5	60	22	14	13	5	7	121
c6	79	19	15	6	1	5	125
c7	20	24	55	11	5	6	121
c8	62	67	39	35	6	3	212
c9	155	21	141	38	10	17	382
c10	16	6	50	17	0	7	96
n	981	424	503	275	83	105	

d — 7-subtype solution

	1	2	3	4	5	6	7	n
c1	68	44	9	15	31	2	25	194
c2	258	137	40	62	39	47	20	603
c3	31	21	21	36	55	2	9	175
c4	82	31	173	14	31	5	6	342
c5	63	7	14	12	13	5	7	121
c6	48	34	19	12	6	1	5	125
c7	6	25	23	46	10	5	6	121
c8	36	46	54	31	36	6	3	212
c9	80	124	17	103	31	10	17	382
c10	9	29	6	28	17	0	7	96
n	681	498	376	359	269	83	105	

d — 8-subtype solution

	1	2	3	4	5	6	7	8	n
c1	77	19	5	11	24	31	2	25	194
c2	313	46	23	24	94	36	47	20	603
c3	43	8	15	20	24	54	2	9	175
c4	89	5	146	3	57	31	5	6	342
c5	49	2	10	11	24	13	5	7	121
c6	46	30	19	8	10	6	1	5	125
c7	20	5	20	46	9	10	5	6	121
c8	69	10	48	15	28	33	6	3	212
c9	144	41	11	78	53	28	10	17	382
c10	15	18	4	23	13	16	0	7	96
n	865	184	301	239	336	258	83	105	

d — 9-subtype solution

	1	2	3	4	5	6	7	8	9	n
c1	64	37	5	11	7	12	31	2	25	194
c2	268	61	23	25	32	89	38	47	20	603
c3	27	20	15	20	4	23	55	2	9	175
c4	71	31	146	3	0	50	30	5	6	342
c5	46	4	10	11	2	23	13	5	7	121
c6	34	42	19	8	2	9	5	1	5	125
c7	7	19	20	46	4	4	10	5	6	121
c8	33	41	48	15	9	25	32	6	3	212
c9	88	70	11	78	30	49	29	10	17	382
c10	11	11	4	23	17	7	16	0	7	96
n	649	336	301	240	107	291	259	83	105	

d — 10-subtype solution

	1	2	3	4	5	6	7	8	9	10	n
c1	59	26	9	11	7	7	32	16	2	25	194
c2	66	180	40	25	32	64	41	88	47	20	603
c3	22	23	21	20	4	16	56	2	2	9	175
c4	43	37	173	3	0	19	31	25	5	6	342
c5	26	40	14	10	2	2	13	2	5	7	121
c6	48	17	19	8	2	5	7	13	1	5	125
c7	10	16	23	46	4	0	10	1	5	6	121
c8	18	55	54	14	8	19	34	1	6	3	212
c9	52	102	17	75	28	33	32	16	10	17	382
c10	14	7	6	22	17	5	16	2	0	7	96
n	358	503	376	234	104	170	272	166	83	105	

f — biopsy site

	breast	brain/CNS	colorectal	liver-biliary	lung/chest wall	lymph node	ovary	skin	soft tissue/bone	stomach/omentum	other
s1	4	36	7	253	53	87	3	9	57	9	9
s2	2	34	5	279	84	107	4	20	118	18	8
s3	3	30	7	247	86	108	9	14	85	21	9
s4	2	22	7	173	84	101	2	21	79	20	7
n	11	122	26	952	307	403	18	64	339	68	33

f — cancer type

	bladder	breast	CNS	colorectal	stomach	head/neck	kidney	liver-biliary	lung	lymphoma	ovary	pancreas	prostate	sarcoma	secretory	skin	thyroid	uterus	eosophagus	cervix	other	n
s1	4	95	6	214	5	12	17	10	8	4	15	10	73	14	4	30	11	0	4	0	9	545
s2	11	125	7	183	10	13	18	14	25	8	22	27	94	33	9	51	12	3	7	1	22	695
s3	6	97	4	162	12	12	15	21	23	7	27	25	97	29	10	38	12	2	8	1	24	632
s4	7	96	6	136	6	14	15	7	19	6	26	17	85	23	4	27	13	4	5	1	16	533
n	28	413	23	695	33	51	65	52	75	25	90	79	349	99	27	146	48	9	24	3	71	

Figure S2. Determining the number of pan-cancer metastasis subtypes. (a) Pan-cancer molecular subtyping of tumor metastases based on TCGA-based subtypes. Pan-cancer molecular subtypes were previously defined using The Cancer Genome Atlas (TCGA) datasets[2]. Gene expression profiles of PDX tumors and patient tumor metastases in our compendium datasets were each classified according to TCGA-based subtype c1 through c10. Expression patterns for the previously-defined top set of 854 mRNAs distinguishing between the ten TCGA-based subtypes[2] are shown for TCGA, PDX, and patient tumor metastases datasets. TCGA subtype-specific expression patterns across the datasets are highlighted. SD, standard deviations from the median within a given dataset and within cancer type. See part b for color coding. **(b)** Expression-based subtyping of patient tumor metastases resulted in subtypes that would be confounded with tissue biopsy site. Consensus ward linkage hierarchical clustering identified k = 10 subtypes represented in our patient tumor metastasis compendium of 2405 metastasis samples. Differential expression patterns for the top 2000 most variable genes used in the clustering are represented. Also represented are genes higher ($p<0.05$, t-test on log2-transformed expression data) in normal liver versus other tissues, based on analysis of the GTex dataset[1]. As seen here, the k7 subtype was highly enriched for metastases sampled from the liver and was strongly associated with liver-specific genes. Also, the k9 subtype was enriched ($p<1E-6$, one-sided Fisher's exact test) for samples from lung, and the k3 subtype was enriched for samples from the lymph node. Problematic results such as the above led us to utilize expression data from PDX models in our final analysis, where the contribution of non-cancer cells would be much less of a factor. For expression heat map, SD represents standard deviations from the median within a given cancer type and within a given dataset. **(c)** For the patient tumor metastasis compendium dataset, the significance of overlap between the patient tumor metastasis-based subtype assignments (from part a) and the final PDX-based subtype assignments used in the final study (from main Figure 1) is indicated. P-values by one-sided Fisher's exact test. Some overlap between the respective assignments is observed, though the final PDX-based subtyping results could be considered the cleaner solution that avoided subtypes strongly associated with biopsy site. **(d)** Determination of pan-cancer subtypes using the PDX compendium dataset. Consensus ward linkage hierarchical clustering identified k = 2 to k = 15 subtypes, based on the 2371 tumors in our PDX compendium dataset. The significance of overlap of previously identified pan-cancer subtypes based on TCGA cohort predominantly representing primary cancer[2] (c1-c10, rows) with subtypes obtained from the PDX compendium dataset. Subtype solutions from 6 subtypes to 10 subtypes are represented here. P-values by one-sided Fisher's exact test. We expected perhaps ten or fewer subtypes, based on previous studies[2-4] (given that subtypes representing immune or stroma infiltration would not be represented in the PDX-based subtypes). Beyond a 7-subtype solution, additional subtypes identified did not encapsulate the previous subtypes and were not well represented in both GEO and PDXNet/PDMR compendium subsets. The 7-subtype solution was therefore selected

and explored further below. **(e)** Across the 2371 PDX tumor expression profiles, differential expression patterns for the set of 2000 genes used to define the original subtypes (left) and for another set of 800 genes (right) found to best distinguish between the respective subtypes in the final 4-subtype solution (top ~200 over-expressed mRNAs for each subtype). Expression values are normalized within each given cancer type and each given dataset (SD, standard deviation from the median within a given cancer type within a given dataset). On the left, the 7-subtype solution from consensus ward linkage hierarchical clustering (part c) is considered, where subtypes 5, 6, and 7 are almost entirely represented in either GEO datasets or the PDXNet/PDMR subsets but not both. In contrast, we believed that robust subtype associations should involve multiple datasets. Therefore, we reclassified the profiles in k subtypes 5-7 according to the best fit among subtypes 1-4 to arrive at the final 4-subtype solution (s1 through s4), represented on the right. **(f)** Left, association of molecular subtype (s1-s4) with metastasis biopsy site; right, association of molecular subtype with cancer type by tissue of origin, based on the patient metastasis compendium dataset. Overall, strong associations are not observed here, except for s1 subtype significantly associating with colorectal tumors (though not exclusive to this cancer type). Of the 695 colorectal metastasis samples in the patient metastasis compendium, 586 were biopsied from the liver (Data File S1). The enrichment of s1 for colorectal tumors would therefore largely explain the more moderate association of s1 with liver biopsy site. Related to Figure 1.

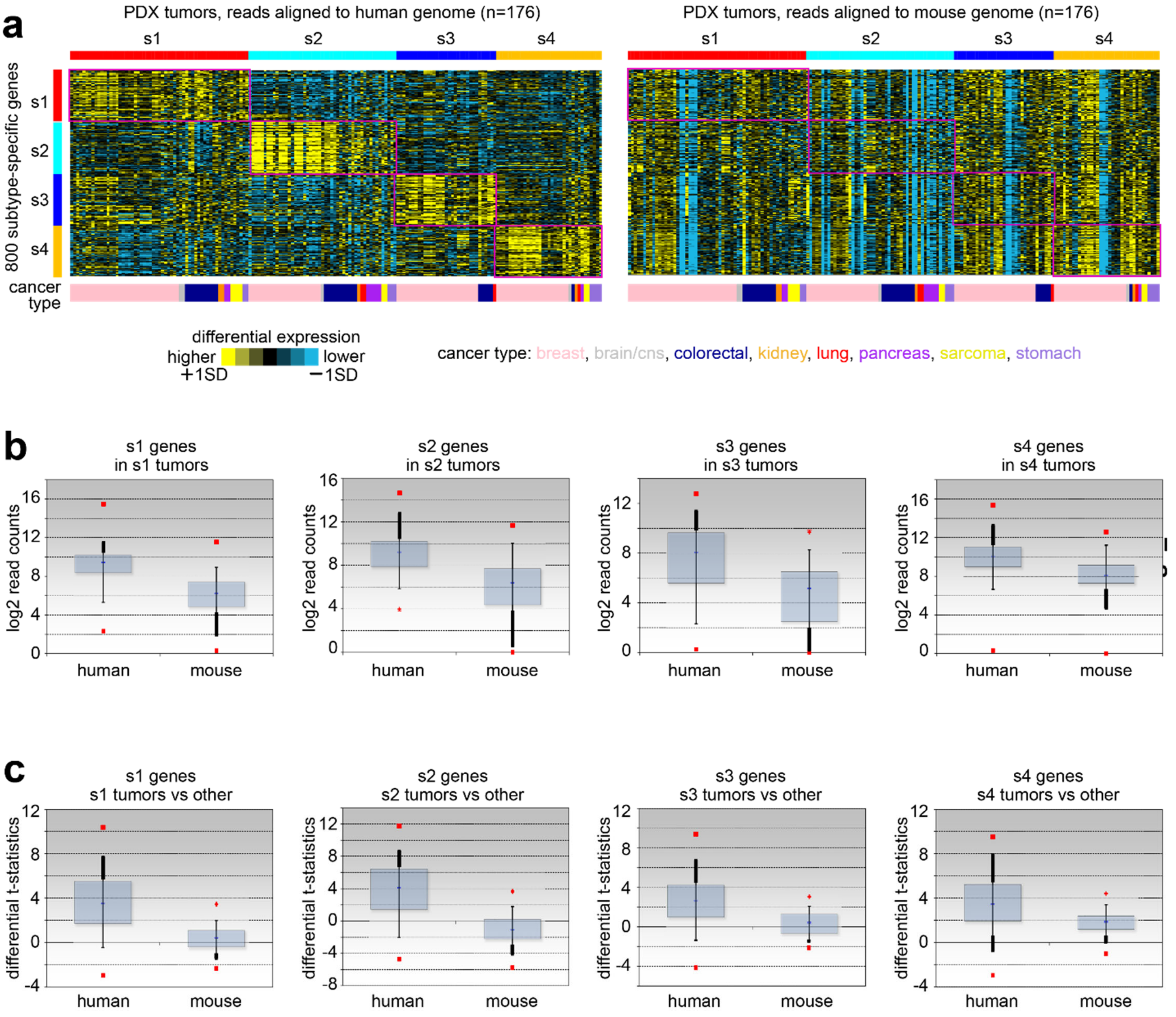

Figure S3. Differential expression patterns associated with PDX-based molecular subtypes are attributable to the cancer cells, not the host. (a) Sequencing reads were aligned to both human and mouse genomes for two of the RNA-seq datasets represented in our PDX compendium (GSE118942[5] and GSE159702[6]). For these two datasets, expression patterns for the top set of 800 subtype-specific mRNAs distinguishing between the four PDX-based subtypes (from Figure 1a) are shown for both the expression data from human genome alignments (left) and the expression data from mouse genome alignments (right). The subtype-specific differential expression patterns are observed here in the version of the dataset based on human genome alignments but not in the version based on mouse genome alignments, indicating that the patterns are specific to the human cells of the tumor and not the mouse cells of the host.

(b) For each of the four subtypes, boxplots of the log2 read counts for the top 100 associated genes (from Figure 1a) based on human versus mouse alignments. On average, the log2 read counts are much higher for human than for mouse. **(c)** For each of the four subtypes, boxplots of the differential t-statistics (comparing for each subtype the average expression levels versus the rest of the tumors by t-test) for the top 100 associated genes (from Figure 1a) based on human versus mouse alignments. Consistent with the heat map representation in part a, the results from the human alignments but not the results from the mouse alignments show statistical significance within the GSE118942/GSE159702 combined dataset. Box plots represent 5% (lower whisker), 25% (lower box), 50% (median), 75% (upper box), and 95% (upper whisker). The above results based on RNA-seq alignments are consistent with analogous experiments previously carried out using expression arrays[7,8], which would be relevant for the expression datasets in the PDX compendium based on array platform. Related to Figure 1.

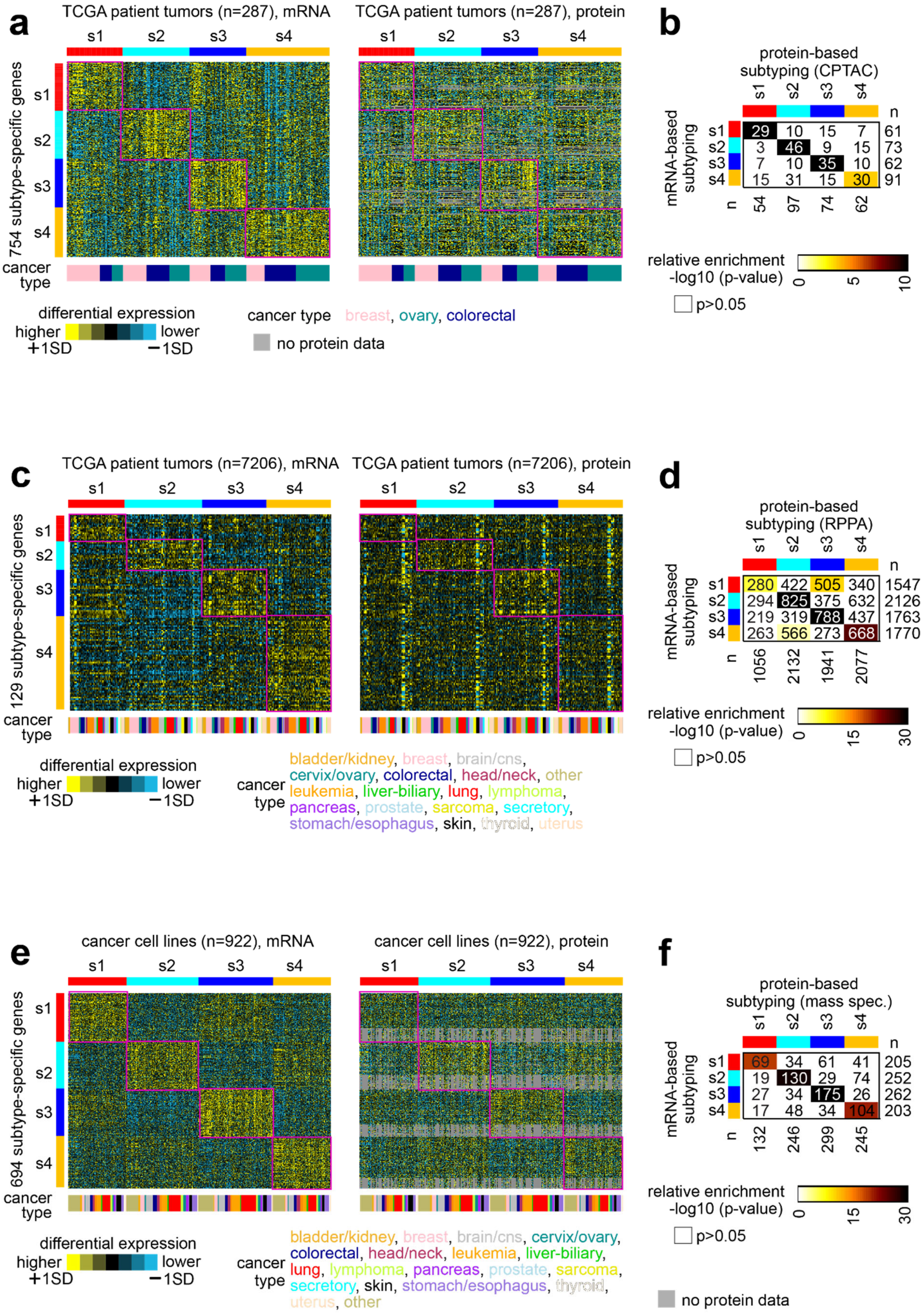
a
TCGA patient tumors (n=287), mRNA
TCGA patient tumors (n=287), protein
s1
s2
s3
s4
754 subtype-specific genes
cancer type
differential expression
higher
lower
+1SD
−1SD
cancer type breast, ovary, colorectal
no protein data
b
protein-based subtyping (CPTAC)
mRNA-based subtyping
n
29 10 15 7 61
3 46 9 15 73
7 10 35 10 62
15 31 15 30 91
54 97 74 62
relative enrichment -log10 (p-value)
0 5 10
p>0.05
c
TCGA patient tumors (n=7206), mRNA
TCGA patient tumors (n=7206), protein
129 subtype-specific genes
bladder/kidney, breast, brain/cns, cervix/ovary, colorectal, head/neck, other leukemia, liver-biliary, lung, lymphoma, pancreas, prostate, sarcoma, secretory, stomach/esophagus, skin, thyroid, uterus
d
protein-based subtyping (RPPA)
280 422 505 340 1547
294 825 375 632 2126
219 319 788 437 1763
263 566 273 668 1770
1056 2132 1941 2077
0 15 30
e
cancer cell lines (n=922), mRNA
cancer cell lines (n=922), protein
694 subtype-specific genes
bladder/kidney, breast, brain/cns, cervix/ovary, colorectal, head/neck, leukemia, liver-biliary, lung, lymphoma, pancreas, prostate, sarcoma, secretory, skin, stomach/esophagus, thyroid, uterus, other
f
protein-based subtyping (mass spec.)
69 34 61 41 205
19 130 29 74 252
27 34 175 26 262
17 48 34 104 203
132 246 299 245
no protein data

Figure S4. Observation of PDX-based molecular subtypes at the proteomic level. (a) The 287 TCGA tumors with mass spectrometry-based proteomic data from the Clinical Proteomic Tumor Analysis Consortium (CPTAC)[3] were classified according to PDX-based molecular subtype (Figure 1a). Expression patterns for a top set of 754 genes distinguishing between the four molecular subtypes based on the PDX transcriptomic compendium dataset (based on available proteomic data from the top 800 mRNAs from Figure 1a) are shown for both the TCGA mRNA dataset (left) and the TCGA proteomic dataset (right). Tumor profiles in both datasets are ordered by PDX-based subtype as assigned using mRNA data. Subtype-specific signature patterns high in each subtype by mRNA data are highlighted. **(b)** For the TCGA tumors with mass spectrometry-based proteomic data, the significance of overlap of the s1-s4 PDX-based subtypes as assigned based on mRNA data (rows) and on mass spectrometry-based proteomic data (columns). Enrichment p-values by one-sided Fisher's exact test. **(c)** The 7206 TCGA tumors with reverse-phase protein array (RPPA) data were classified according to PDX-based molecular subtype (from Figure 1a). Expression patterns for a top set of 129 genes distinguishing between the four molecular subtypes based on the PDX transcriptomic compendium dataset (see the section "Methods", based on available data) are shown for both the TCGA mRNA dataset (left) and the TCGA RPPA proteomic dataset (right). Tumor profiles in both datasets are ordered by PDX-based subtype as assigned using mRNA data. Subtype-specific signature patterns high in each subtype by mRNA data are highlighted. **(d)** For the TCGA tumors with RPPA data, the significance of overlap of the s1-s4 PDX-based subtypes as assigned based on mRNA data (rows) and on RPPA data (columns). Enrichment p-values by one-sided Fisher's exact test. The limitations of the proteomics-based analyses would include more limited proteomic data availability on tumors compared to mRNA and the fact that mRNA and protein levels do not highly correlate across tumors[3]. Despite these limitations, significant levels of concordance are observed here between the mRNA-based subtyping and the protein-based subtyping (parts b and d). **(e)** Observation of PDX-based molecular subtypes at the proteomic level in cancer cell lines. The 922 cell lines with both mRNA data and mass spectrometry-based proteomic data [9] were classified according to PDX-based molecular subtype (from Figure 1a). Expression patterns for a top set of 694 mRNA distinguishing between the four molecular subtypes based on the PDX transcriptomic compendium dataset (based on available proteomic data from the top 800 mRNAs from Figure 1a) are shown for both the mRNA dataset (left) and the proteomic dataset (right). Cell line profiles in both datasets are ordered by PDX-based subtype as assigned using mRNA data. Subtype-specific signature patterns high in each subtype by mRNA data are highlighted. **(f)** For the 922 cell lines, the significance of overlap of the s1-s4 PDX-based subtypes as assigned based on mRNA data (rows) and on mass spectrometry-based proteomic data (columns). Enrichment p-values by one-sided Fisher's exact test. Related to Figure 1.

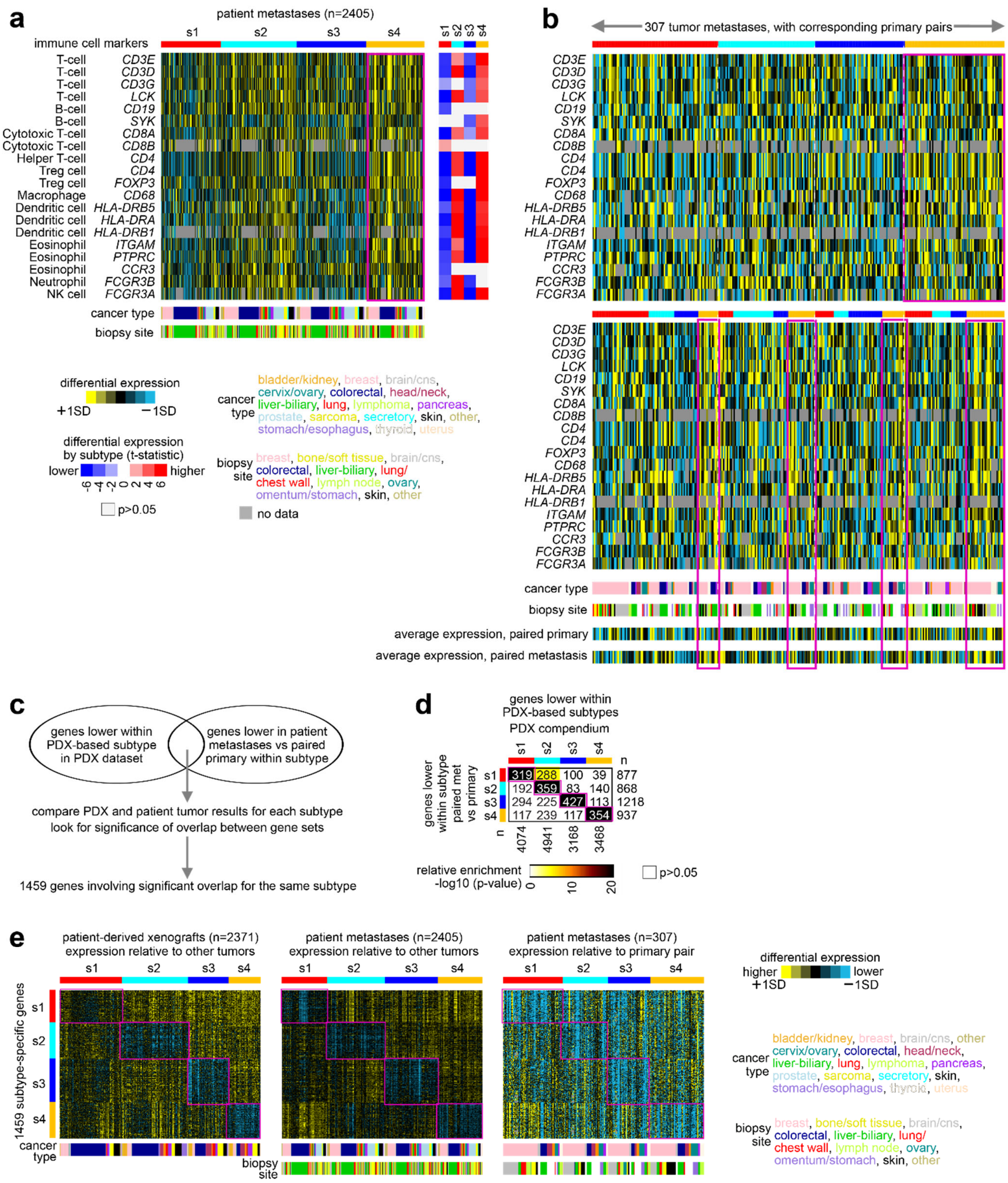
a
patient metastases (n=2405)
s1 s2 s3 s4
immune cell markers
T-cell CD3E
T-cell CD3D
T-cell CD3G
T-cell LCK
B-cell CD19
B-cell SYK
Cytotoxic T-cell CD8A
Cytotoxic T-cell CD8B
Helper T-cell CD4
Treg cell CD4
Treg cell FOXP3
Macrophage CD68
Dendritic cell HLA-DRB5
Dendritic cell HLA-DRA
Dendritic cell HLA-DRB1
Eosinophil ITGAM
Eosinophil PTPRC
Eosinophil CCR3
Neutrophil FCGR3B
NK cell FCGR3A
cancer type
biopsy site
differential expression
+1SD −1SD
differential expression by subtype (t-statistic)
lower higher
-6 -4 -2 0 2 4 6
p>0.05
cancer type
bladder/kidney, breast, brain/cns, cervix/ovary, colorectal, head/neck, liver-biliary, lung, lymphoma, pancreas, prostate, sarcoma, secretory, skin, other, stomach/esophagus, thyroid, uterus
biopsy site
breast, bone/soft tissue, brain/cns, colorectal, liver-biliary, lung/ chest wall, lymph node, ovary, omentum/stomach, skin, other
no data
b
307 tumor metastases, with corresponding primary pairs
paired primary tumors
tumor metastases
cancer type
biopsy site
average expression, paired primary
average expression, paired metastasis
c
genes lower within PDX-based subtype in PDX dataset
genes lower in patient metastases vs paired primary within subtype
compare PDX and patient tumor results for each subtype
look for significance of overlap between gene sets
1459 genes involving significant overlap for the same subtype
d
genes lower within PDX-based subtypes
PDX compendium
genes lower within subtype paired met vs primary
s1 s2 s3 s4 n
s1 319 288 100 39 877
s2 192 359 83 140 868
s3 294 225 427 113 1218
s4 117 239 117 354 937
n 4074 4941 3168 3468
relative enrichment -log10 (p-value)
0 10 20
p>0.05
e
patient-derived xenografts (n=2371)
expression relative to other tumors
patient metastases (n=2405)
expression relative to other tumors
patient metastases (n=307)
expression relative to primary pair
1459 subtype-specific genes
cancer type
biopsy site
differential expression
higher lower
+1SD −1SD

Figure S5. Additional information involving paired metastasis-primary comparisons (a) Expression changes in immune cell markers involving the s4 subtype. For the patient tumor metastases compendium dataset, the heat map shows differential patterns for key genes representing immune cell markers[4]. On the right are the corresponding gene-level t-statistics (by t-test), comparing tumors in the given subtype with the rest of the tumors. Most of these markers tend to be highest in the s4 subtype. **(b)** For the 307 patient tumor metastasis expression profiles for which expression profiles for the paired primary were available, both the primary and the metastasis were classified for the PDX-based subtypes (Figure 2c). The expression heat map represents the immune cell marker expression patterns of the tumor metastases in relation to the patterns for the corresponding paired primary tumors. In many instances, subtype switching events in patient metastases could be associated with expression changes in immune cell markers involving the s4 subtype. These patterns are highlighted, for example, in the heat maps in part b showing the average differential expression patterns in the paired primary versus paired metastasis samples. **(c)** Subtype-specific gene expression differences overlap highly with metastasis versus paired primary differences, focusing on the genes under-expressed by subtype. Schematic of gene set comparisons. For each PDX-based subtype, the set of genes low within that subtype versus the rest of the PDX tumors were overlapped with the set of genes low in patient metastases of the same PDX-based subtype versus the corresponding paired primaries. A set of 1459 genes involve significant gene set overlaps ($p<1E-10$, one-sided Fisher's exact test) between PDX comparisons and paired patient metastasis comparisons for the same subtype, involving all four subtypes. **(d)** Significance of overlap between the genes low within each of the PDX-based subtypes (using t-test $p<0.01$, based on analysis of PDX compendium) and the genes low within paired patient metastasis versus primary within each subtype ($p<0.01$, paired t-test, based on analysis of the patient tumor metastasis compendium). P-values by one-sided Fisher's exact test. From these results, a set of 1459 genes involve significant gene set overlap ($p<0.0001$) for the same subtypes (e.g., 319 overlapping s1-s1 genes, 359 s2-s2 genes, etc.). **(e)** Differential expression patterns for the top set of 1459 genes involving significant gene set overlaps for any of the four PDX-based subtypes are shown across the PDX compendium dataset (differential expression relative to other tumors), patient tumor metastases compendium dataset (relative to other tumor metastases), and patient tumor metastasis versus paired primary compendium dataset (relative to primary pair). Subtype-specific expression patterns are highlighted. Related to Figure 3.

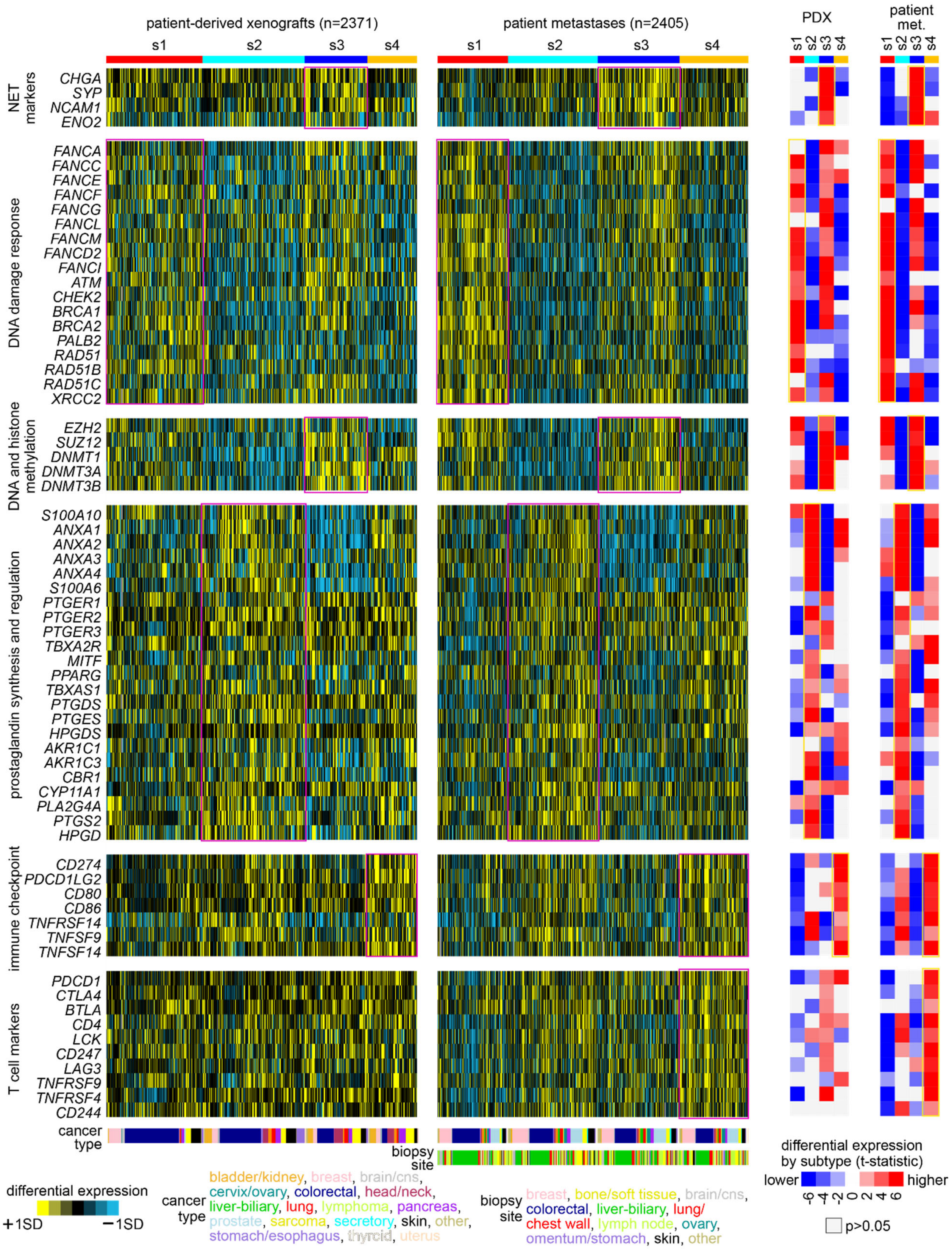

patient-derived xenografts (n=2371)
patient metastases (n=2405)
s1
s2
s3
s4
PDX
patient met.
NET markers
CHGA
SYP
NCAM1
ENO2
DNA damage response
FANCA
FANCC
FANCE
FANCF
FANCG
FANCL
FANCM
FANCD2
FANCI
ATM
CHEK2
BRCA1
BRCA2
PALB2
RAD51
RAD51B
RAD51C
XRCC2
DNA and histone methylation
EZH2
SUZ12
DNMT1
DNMT3A
DNMT3B
prostaglandin synthesis and regulation
S100A10
ANXA1
ANXA2
ANXA3
ANXA4
S100A6
PTGER1
PTGER2
PTGER3
TBXA2R
MITF
PPARG
TBXAS1
PTGDS
PTGES
HPGDS
AKR1C1
AKR1C3
CBR1
CYP11A1
PLA2G4A
PTGS2
HPGD
immune checkpoint
CD274
PDCD1LG2
CD80
CD86
TNFRSF14
TNFSF9
TNFSF14
T cell markers
PDCD1
CTLA4
BTLA
CD4
LCK
CD247
LAG3
TNFRSF9
TNFRSF4
CD244
cancer type
biopsy site
differential expression
+1SD
−1SD
cancer type
bladder/kidney, breast, brain/cns, cervix/ovary, colorectal, head/neck, liver-biliary, lung, lymphoma, pancreas, prostate, sarcoma, secretory, skin, other, stomach/esophagus, thyroid, uterus
biopsy site
breast, bone/soft tissue, brain/cns, colorectal, liver-biliary, lung/ chest wall, lymph node, ovary, omentum/stomach, skin, other
differential expression by subtype (t-statistic)
lower
higher
-6 -4 -2 0 2 4 6
p>0.05

Figure S6. Differential expression patterns involving key genes in specific pathways. For PDX and patient tumor metastases compendium datasets, heat maps show differential patterns for key genes of interest highlighted elsewhere (e.g., genes in pathways highlighted in Figure 6). On the right are the corresponding gene-level t-statistics (by t-test), comparing tumors in the given subtype with the rest of the tumors. Related to Figure 6.

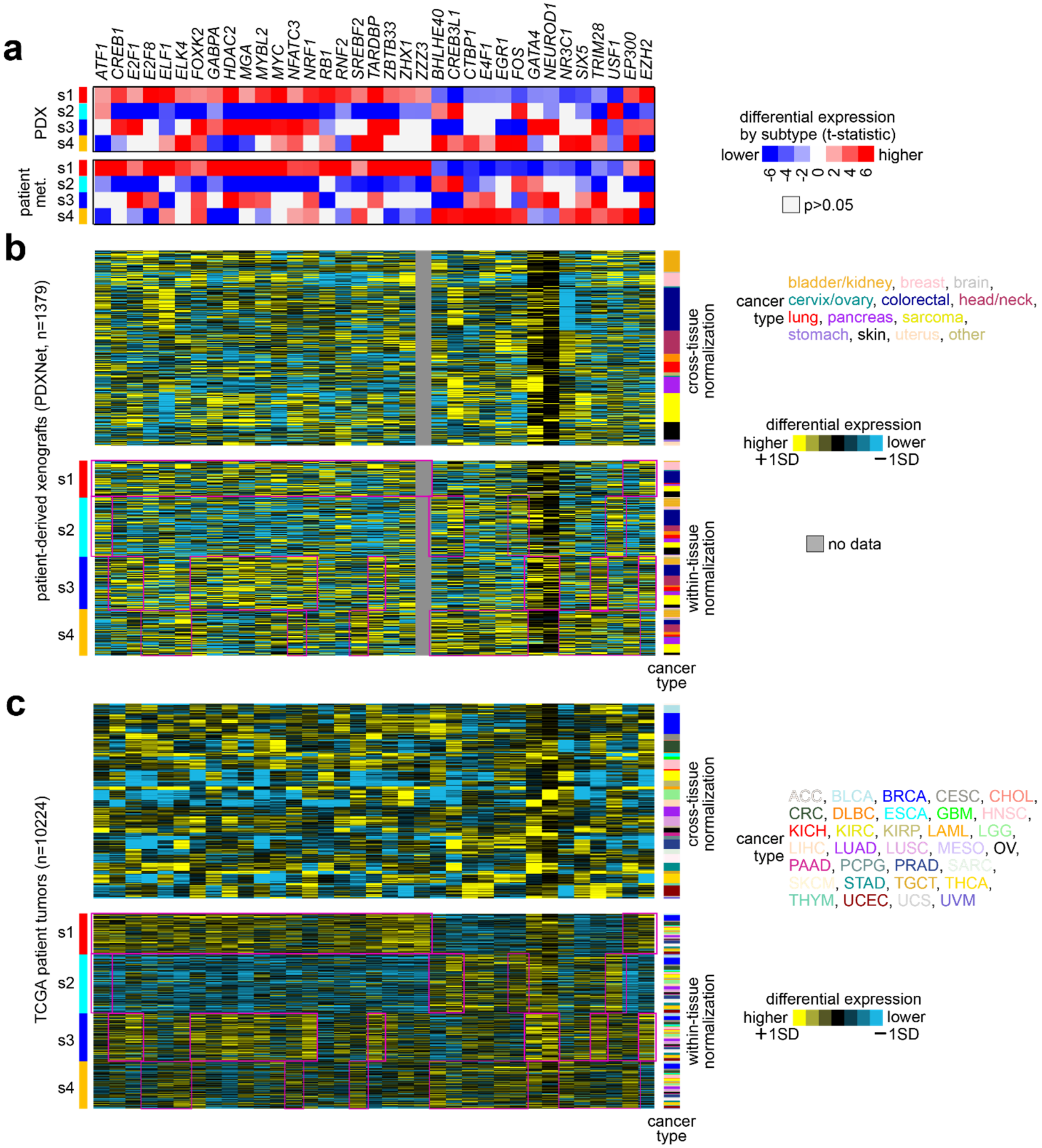

Figure S7. Lineage-specific gene expression patterns involving TFs with associations by subtype. (a) Top TF associations by subtype, taken from Figure 4b. The differential expression statistics by PDX-based subtype are represented for the top set of 35 TFs with both significant overlap between the TF-bound genes and genes over-expressed in the expression subtype and significantly higher or lower levels of the TF gene in that same subtype (both PDX and patient metastasis compendium datasets). **(b)** For the TF genes in part a, the corresponding differential expression patterns in the PDXNet dataset. The PDXNet dataset is normalized in two

ways: by cross-tissue normalization (top, values normalized to standard deviations from the median across all tumors) and within-tissue normalization (bottom, values normalized within each cancer type to standard deviations from the median). **(c)** Similar to part b, representing the TCGA pan-cancer dataset as normalized in the two different ways. We used the within-tissue normalization approach to compile our compendium expression datasets and the downstream analyses. By design, tissue-specific or lineage-specific expression differences are removed by within-tissue normalization, allowing us to identify pan-cancer subtypes that would transcend tissue- or cell-of-origin. The PDXNet and TCGA datasets feature cancers of various types profiled uniformly on a common platform, allowing us to examine expression patterns by cross-tissue normalization. With cross-tissue normalization, lineage-specific TF gene expression patterns can be observed. As intended, within-tissue normalization allows us to identify uniform differential patterns within a given pan-cancer subtype. Related to Figure 4.

References

1. Lek, M., Karczewski, K.J., Minikel, E.V., Samocha, K.E., Banks, E., Fennell, T., O'Donnell-Luria, A.H., Ware, J.S., Hill, A.J., Cummings, B.B., et al. (2016). Analysis of protein-coding genetic variation in 60,706 humans. Nature *536*, 285-291. 10.1038/nature19057.
2. Chen, F., Zhang, Y., Gibbons, D., Deneen, B., Kwiatkowski, D., Ittmann, M., and Creighton, C. (2018). Pan-cancer molecular classes transcending tumor lineage across 32 cancer types, multiple data platforms, and over 10,000 cases. Clin Cancer Res. *24*, 2182-2193.
3. Zhang, Y., Chen, F., Chandrashekar, D., Varambally, S., and Creighton, C. (2022). Proteogenomic characterization of 2002 human cancers reveals pan-cancer molecular subtypes and associated pathways. Nat Commun *13*, 2669.
4. Chen, F., Chandrashekar, D., Varambally, S., and Creighton, C. (2019). Pan-cancer molecular subtypes revealed by mass-spectrometry-based proteomic characterization of more than 500 human cancers. Nat Commun *10*, 5679.
5. Alzubi, M., Turner, T., Olex, A., Sohal, S., Tobin, N., Recio, S., Bergh, J., Hatschek, T., Parker, J., Sartorius, C., et al. (2019). Separation of breast cancer and organ microenvironment transcriptomes in metastases. Breast Cancer Res *21*, 36.
6. Sueyoshi, K., Komura, D., Katoh, H., Yamamoto, A., Onoyama, T., Chijiwa, T., Isagawa, T., Tanaka, M., Suemizu, H., Nakamura, M., et al. (2021). Multi-tumor analysis of cancer-stroma interactomes of patient-derived xenografts unveils the unique homeostatic process in renal cell carcinomas. iScience *24*, 10332.
7. Creighton, C.J., Bromberg-White, J.L., Misek, D.E., Monsma, D.J., Brichory, F., Kuick, R., Giordano, T.J., Gao, W., Omenn, G.S., Webb, C.P., and Hanash, S.M. (2005). Analysis of tumor-host interactions by gene expression profiling of lung adenocarcinoma xenografts identifies genes involved in tumor formation. Mol Cancer Res *3*, 119-129.
8. Creighton, C., Kuick, R., Misek, D., Rickman, D., Brichory, F., Rouillard, J.-M., Omenn, G., and Hanash, S. (2003). Profiling of pathway-specific changes in gene expression following growth of human cancer cell lines transplanted into mice. Genome biology *4*, R46.
9. Gonçalves, E., Poulos, R., Cai, Z., Barthorpe, S., Manda, S., Lucas, N., Beck, A., Bucio-Noble, D., Dausmann, M., Hall, C., et al. (2022). Pan-cancer proteomic map of 949 human cell lines. Cancer Cell *40*, 835-849.
